# Singly and Triply Linked Magnetic Porphyrin Lanthanide Arrays

**DOI:** 10.1021/jacs.2c02084

**Published:** 2022-05-03

**Authors:** Jeff M. Van Raden, Dimitris I. Alexandropoulos, Michael Slota, Simen Sopp, Taisuke Matsuno, Amber L. Thompson, Hiroyuki Isobe, Harry L. Anderson, Lapo Bogani

**Affiliations:** † Department of Chemistry, University of Oxford, Chemistry Research Laboratory, Oxford OX1 3TA, U.K.; ‡ Department of Materials, University of Oxford, Oxford OX1 3PH, U.K.; § Department of Chemistry, The University of Tokyo, Tokyo 113-0033, Japan

## Abstract

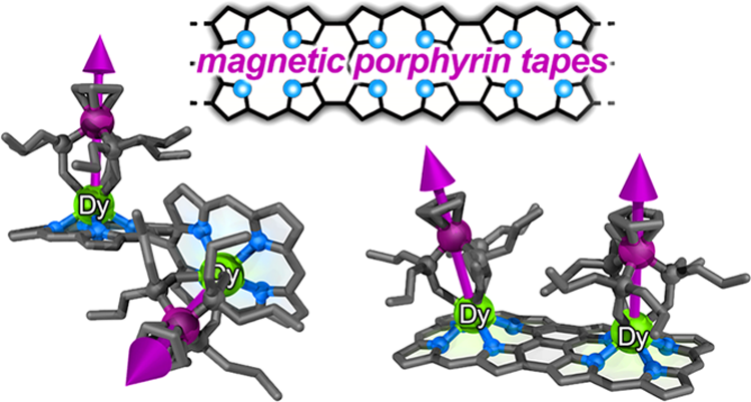

The introduction of paramagnetic metal centers into a conjugated π-system is a promising approach toward engineering spintronic materials. Here, we report an investigation of two types of spin-bearing dysprosium(III) and gadolinium(III) porphyrin dimers: singly *meso–meso*-linked dimers with twisted conformations and planar edge-fused β,*meso*,β-linked tapes. The rare-earth spin centers sit out of the plane of the porphyrin, so that the singly linked dimers are chiral, and their enantiomers can be resolved, whereas the edge-fused tape complexes can be separated into *syn* and *anti* stereoisomers. We compare the crystal structures, UV–vis–NIR absorption spectra, electrochemistry, EPR spectroscopy, and magnetic behavior of these complexes. Low-temperature SQUID magnetometry measurements reveal intramolecular antiferromagnetic exchange coupling between the Gd^III^ centers in the edge-fused dimers (*syn* isomer: *J* = −51 ± 2 MHz; *anti* isomer: *J* = −19 ± 3 MHz), whereas no exchange coupling is detected in the singly linked twisted complex. The phase-memory times, *T*_m_, are in the range of 8–10 μs at 3 K, which is long enough to test quantum computational schemes using microwave pulses. Both the *syn* and *anti* Dy_2_ edge-fused tapes exhibit single-molecule magnetic hysteresis cycles at temperatures below 0.5 K with slow magnetization dynamics.

## Introduction

Graphene-like materials with extensive π-delocalization exhibit remarkable electronic and physical properties.^1^ One poorly explored aspect is the injection of spin into the delocalized states of a π-conjugated backbone.^2^ The deposition of single-molecule magnets (SMMs) on graphene revealed not only sizable spin-electron interaction but also the possibility of driving the spin dynamics into fully quantum regimes, such as Villain’s tunneling region.^3^ On the other hand, we lack fundamental information about how to engineer such interactions: How are spin interactions transmitted along a π-conjugated plane? How do they behave when spins are on the same or opposite sides of the plane? What happens when a twist blocks π-conjugation? And how does conjugation influence the spin dynamics?

Previously, attempts have been made to address these questions by depositing metals and magnetic molecules on graphene,^4^ but this results in random molecular placement. The chemical doping of graphene yields structures that are poorly defined at the atomic level, hampering the elucidation of structure–property relationships. For example, although the edges of graphene nanoribbons had long been proposed to exhibit ferromagnetism, spin-filtering capabilities,^5^ and quantum coherence features,^6^ only the advent of molecular graphene nanoribbons with atomically precise structures enabled the experimental investigation of magnetic edge states.^7^ Molecular metal coordination complexes with π-conjugated backbones and a few spins offer unexplored opportunities to address these issues by providing spin-functionalized conjugated frameworks,^8^ with atomic-level control, enabling spin–spin interactions to be rationalized, and the best frameworks selected.

**Figure 1:**
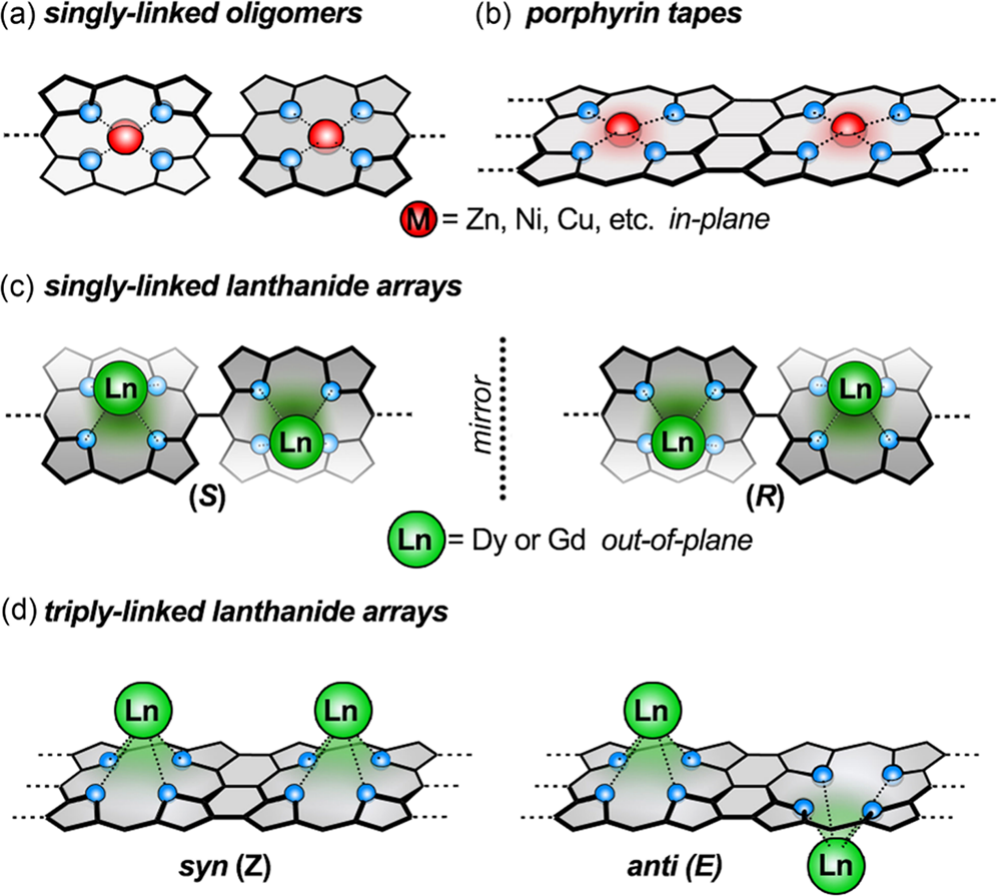
Cartoon representations of (a) singly and (b) triply linked porphyrin oligomers; (c) Ln^III^-derived axially chiral singly linked porphyrin arrays; and (d) triply linked porphyrin arrays.

Ln^III^-based single-molecule magnet (SMM)^9^ can offer high blocking temperatures and ultrahard magnetic behavior.^10^ Moreover, Ln^III^ complexes afford an extreme level of tuning of the magnetic properties by changing the rare-earth, without altering the chemistry or the structural features. Changing the Ln^III^ cation provides control over the spin–orbit coupling and thus the interplay of electronic and spin degrees of freedom in the conjugated backbone and facilitates the elucidation of both the SMM behavior and coherent states. Metalation of porphyrins with Ln^III^ cations is thus an excellent strategy to introduce spin into π-conjugated materials and to investigate magnetic coupling through large aromatic π-systems. Previously, we have shown that butadiyne-linked lanthanide porphyrin dimers exhibit slow magnetic relaxation below 10 K under a static magnetic field and that they provide the necessary elements for the construction of a single-molecule spin valve.^11^*Meso-*singly linked porphyrin oligomers^12^ (Figure 1a) and β,*meso*,β-edge-fused porphyrin tapes^13^ (Figure 1b) can be regarded as yin and yang structures: their connectivity is similar but they display opposite types of electronic coupling. Single-linked chains are highly twisted, with neighboring porphyrins almost orthogonal, preventing orbital overlap, but there is a strong through-space exciton coupling between the porphyrins, and the chains behave as photonic wires.^14^ In contrast, the fused tapes have flat π-systems with strong π-conjugation; their π–π* energy gaps fall steeply with increasing oligomer length,^13,15^ and their single-molecule conductances are almost independent of length.^16^ Diamagnetic porphyrin oligomers, containing zinc(II) or nickel(II) cations, have been thoroughly investigated, but there have been a few studies of the magnetic properties of singly linked oligomers and triply linked tapes hosting paramagnetic metal centers.^17,18^ Here, we investigate both singly linked and fused porphyrin dimers with dysprosium(III) or gadolinium(III) centers as models for longer oligomer with many lanthanide metal cations. In contrast to metals such as Zn^II^, Cu^II^, and Ni^II^, which sit in the plane of the porphyrin, Dy^III^ and Gd^III^ sit out of plane, leading to interesting issues of stereochemistry. We investigate axially chiral singly linked dinuclear complexes (Figure 1c) and triply linked stereoisomeric dinuclear metal complexes: the *syn* (*Z*) isomer, in which both lanthanide metal centers are on the same face of the π-system, and the *anti* (*E*) isomer, in which the metals are on opposite faces (Figure 1d).

**Scheme 1:**
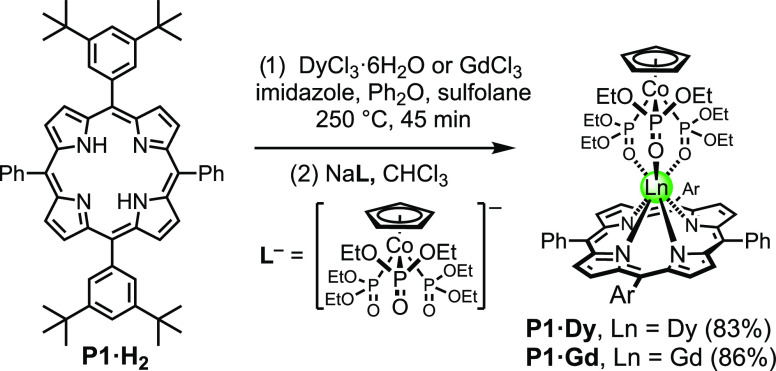
Synthesis of Metalloporphyrin Monomer **P1·M_2_** Ar = 3,5-di(*t*-butyl)phenyl.

**Scheme 2:**
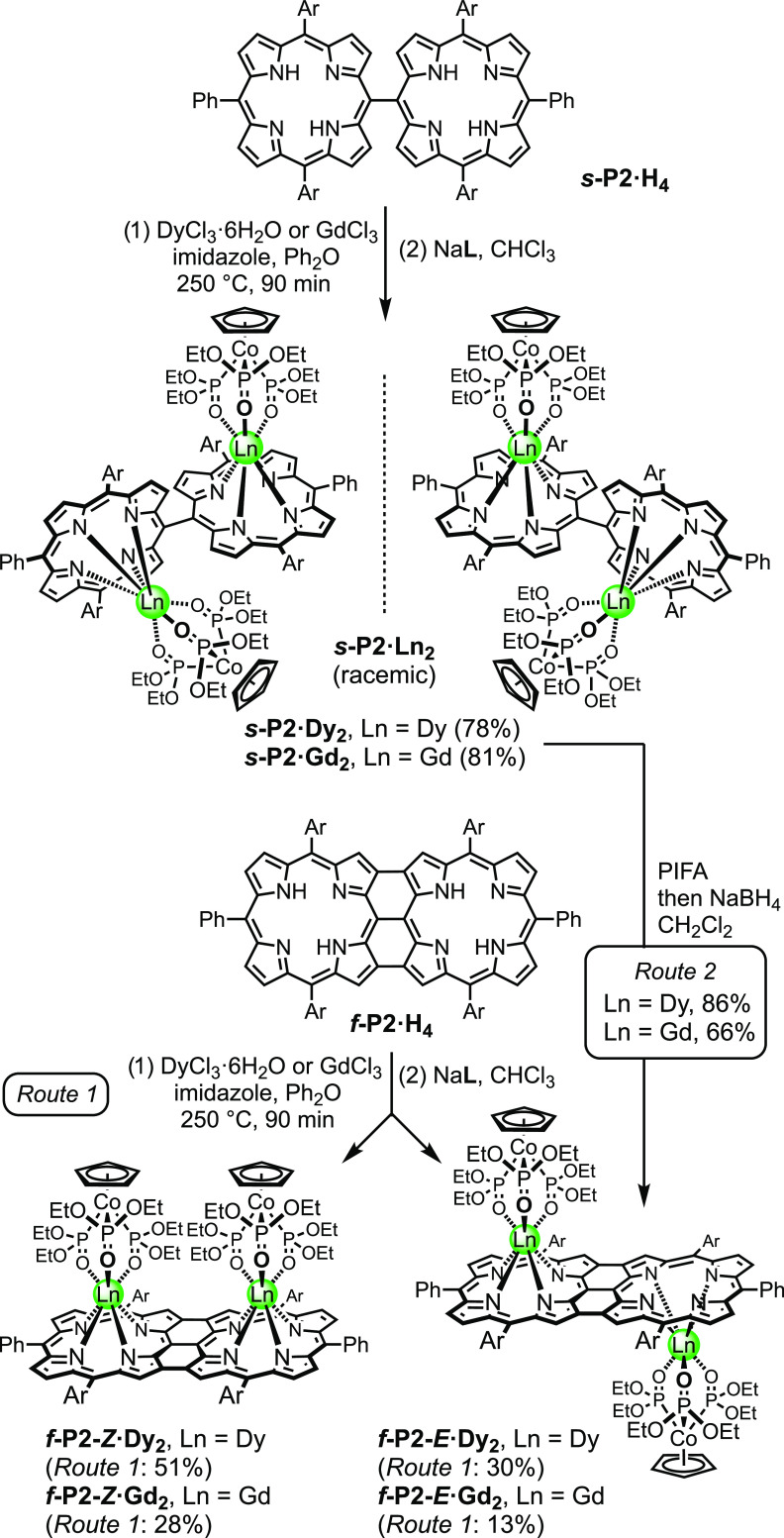
Synthesis of the Metalloporphyrin Dimers: (a) Racemic ***s*-P2·M_2_** and (b) ***f-*P2-*Z*·M_2_** and ***f*-P2-*E*·M_2_** Ar = 3,5-di(*t*-butyl)phenyl and L = Kläui ligand.

In this study, we investigate the structure–property relations in porphyrin oligomers coordinating Ln^III^ centers. We test the SMM behavior and the quantum coherence times while varying the Ln^III^ metal cation and the stereochemistry; for example, metal ions sitting on the same or opposite sides of the π-conjugated plane give distinctively different coherence and hysteresis. We compare the properties of a lanthanide porphyrin monomer, **P1·Ln** (Scheme 1; Ln = Dy or Gd), with two types of dimers: ***s*****-P2·Ln**_**2**_ (as two enantiomers) and ***f*****-P2·Ln**_**2**_ (as two diastereomers, *E* and *Z*; Scheme 2). In all of these complexes, the lanthanide metal centers are protected by the Kläui capping ligand.^11,19,20^ This anionic cap is an important part of the molecular design because it results in neutral complexes that are soluble in nonpolar organic solvents, kinetically stable, and easy to purify by chromatography on silica.^11,19^ The diamagnetic Co^III^ cation of this capping group does not significantly influence the magnetic properties. The crystal structures of ***s*****-P2·Dy**_**2**_, ***f*****-P2-*****Z*****·Dy**_**2**_, and ***f*****-P2-*****E*****·Dy**_**2**_ confirm their identities, while UV–vis–NIR absorption spectra and electrochemical measurements reveal differences in the electronic structure. The impact of the stereochemistry and connectivities on the static and dynamic magnetic properties has been tested, including the coherence properties of porphyrin dimers bearing Gd^III^ centers, ***s*****-P2·Gd**_**2**_, ***f*****-P2-*****Z*****·Gd**_**2**_, and ***f*****-P2**-***E*****·Gd**_**2**_.

## Results and Discussion

### Synthesis

The reference porphyrin monomer dysprosium and gadolinium complexes, **P1·Dy** and **P1·Gd**, and the dimers ***s*****-P2·Dy**_**2**_, ***f*****-P2*****-Z*****·Dy**_**2**_, ***f*****-P2*****-E*****·Dy**_**2**_, ***s*****-P2·Gd**_**2**_, ***f*****-P2-*****Z*****·Gd**_**2**_, and ***f*****-P2-*****E*****·Gd**_**2**_ were synthesized from the corresponding free-base porphyrins by treatment with the lanthanide(III) chlorides in diphenyl ether at 250 °C, as shown in Schemes 1 and 2.^11,20^ Use of sulfolane as a cosolvent was found to accelerate metalation of the porphyrin monomer, **P1·H**_**2**_,^21^ reducing the reaction time to 45 min, which is beneficial because long reaction times at this temperature result in decomposition. After insertion of the metal, Kläui’s anionic capping ligand (**L^–^**) was coordinated to the metalloporphyrins at room temperature.^19^

In contrast to **P1·H**_**2**_, the use of sulfolane significantly reduced the yield for metalation of the dimers ***s*****-P2·H**_**4**_ and ***f*****-P2·H**_**4**_. Consequently, sulfolane was omitted (Scheme 2) and these dimers required longer reaction times. As for **P1·Ln**, the singly linked products, ***s*****-P2·Ln**_**2**_, were purified by silica gel chromatography. The enantiomers were then resolved by chiral HPLC using a SUMICHIRAL column as discussed below. Separation of the *syn* and *anti* isomers (***f-*****P2-*****Z*****·Ln**_**2**_ and ***f*****-P2-*****E*****·Ln**_**2**_, respectively) was accomplished by silica gel chromatography, followed by crystallization. As expected, the *syn* isomers are more polar than the *anti* isomers, leading to a lower chromatographic mobility on silica. Analysis of the crude reaction mixture of the Dy^III^ reaction via gel-permeation chromatography (GPC) revealed an approximate 2:1 ratio of *Z*/*E* isomers, and this observation is reflected in the isolated yields. It is surprising that the *syn* isomers predominate in these reactions, and it suggests an attractive interaction between the two metal centers.

We also synthesized ***f*****-P2-*****E*****·Dy**_**2**_ and ***f*****-P2-*****E*****·Gd**_**2**_ in high yield by the Scholl reaction of ***s*****-P2·Dy**_**2**_ and ***s*****-P2·Dy**_**2**_, respectively, using phenyliodine(III) bis(trifluoroacetate) (PIFA), followed by workup with sodium borohydride (Scheme 2; route 2).^22^ It is surprising that the organometallic Kläui ligand survives these strongly oxidizing conditions and that this reaction proceeds so efficiently. It is also surprising that the oxidation of ***s*****-P2·Ln**_**2**_ gives exclusively ***f*****-P2-*****E*****·Ln**_**2**_, without forming detectable amounts of the *Z-*isomer, whereas metalation of ***f*****-P2·H**_**4**_ gives predominantly the *Z*-isomer. The explanation for this difference in stereochemical outcome is probably that metalation occurs before the bulky Kläui ligand has been installed when there is no steric clash between the metal centers, whereas the Scholl reaction (route 2) is carried out with the bulky capping ligands in place. The efficient and highly stereoselective formation of ***f*****-P2-*****E*****·Dy**_**2**_ from ***s*****-P2·Dy**_**2**_ suggests that this route could be extended to prepare lanthanide complexes of long porphyrin oligomer tapes without forming mixtures of stereoisomers.

### X-ray Crystallography^23^

**Figure 2:**
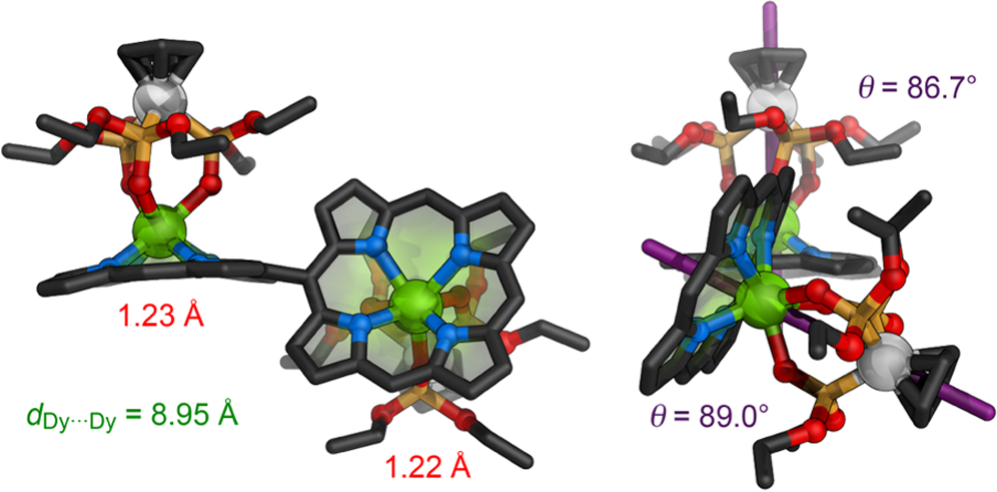
Crystal structure of ***s*****-P2·Dy**_**2**_ from X-ray diffraction studies showing side (left) and axial views (right). Solvent molecules, hydrogen atoms, aryl groups, and minor components of disorder are omitted for clarity. The violet lines indicate the Co–Dy vectors. The distances of the Dy atoms from the mean planes of the four nitrogen atoms are shown in red, and the angle θ of the Co–Dy vectors to the planes for the four nitrogen atoms are shown in purple.

Single crystals of ***s*****-P2·Dy**_**2**_ and ***s*****-P2·Gd**_**2**_ suitable for X-ray diffraction were grown via liquid–liquid diffusion of methanol into chloroform solutions. The structures of these Dy and Gd complexes were found to be isomorphous and isostructural in the crystalline solid state, so we only discuss ***s*****-P2·Dy**_**2**_ here (Figure 2). The structure of ***s*****-P2·Gd**_**2**_ is included in the Supporting Information (CIF). The capped Ln^III^ metal cation coordinates to one face of the porphyrin, making the singly linked porphyrin dimers axially chiral, and they crystallize as racemates. Both the Kläui capping groups in this structure are rotationally disordered, reflecting a shallow energy profile for rotation about the Dy–Co axis. The angle between the planes defined by the four nitrogen atoms of the two porphyrins is 62.77(17)°, which is smaller than might be expected. For example, the corresponding angles in the crystal structures of two similar singly linked zinc porphyrin dimer units are 69 and 72°.^12c,24^ The smaller torsional angle in ***s*****-P2·Dy**_**2**_ may be a consequence of the steric bulk of the lanthanide capping group, which would clash with the aryl group of the neighboring porphyrin if the two porphyrins were orthogonal. The distances of the Dy^III^ centers from the mean planes of the porphyrins (defined by the four nitrogen atoms) are 1.22(1) and 1.23(1) Å, similar to those reported in related Dy^III^ porphyrin complexes.^11,20^ The intramolecular Dy···Dy distance is 8.9451(4) Å, compared with 8.30 and 8.41 Å in the analogous zinc complexes.^12c,24^ The Co–Dy vectors are almost perpendicular to the mean planes of nitrogen atoms of each porphyrin (θ = 86.68(12) and 89.01(14)°; Figure 2).

**Figure 3:**
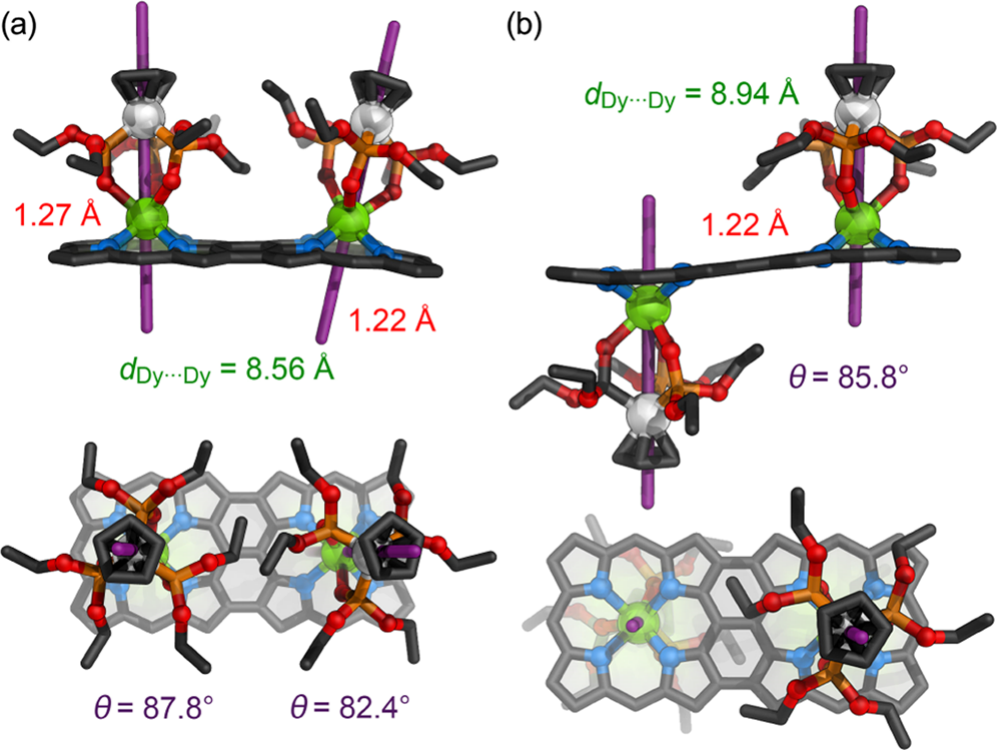
Crystal structure of (a) ***f*****-P2-*****Z*****·Dy**_**2**_ and (b) ***f*****-P2-*****E*****·Dy**_**2**_ from X-ray diffraction studies showing side and top views. Solvent molecules, hydrogen atoms, aryl groups, and minor components of disorder are omitted for clarity. The violet lines indicate the Co–Dy vectors. The distances of the Dy atoms from the mean planes of the four nitrogen atoms are shown in red, and the angle θ of the Co–Dy vectors to the planes for the four nitrogen atoms is shown in purple.

Single crystals of the edge-fused lanthanide porphyrin dimers were grown via liquid–liquid diffusion by layering ethyl acetate or methanol over chloroform solutions of ***f*****-P2-*****Z*****·Dy**_**2**_ or ***f*****-P2-*****E*****·Dy**_**2**_, respectively. Both structures (Figure 3) have half a porphyrin dimer molecule in the asymmetric unit: the two halves of the ***f*****-P2-*****Z*****·Dy**_**2**_ molecule are related by a crystallographic mirror plane, which lies in the Dy_2_Co_2_ plane, whereas the ***f*****-P2-*****E*****·Dy**_**2**_ molecule occupies a position on an inversion center. The intramolecular Dy···Dy distances are 8.5561(9) Å in ***f*****-P2-*****Z*****·Dy**_**2**_ and 8.9371(9) Å in ***f*****-P2-*****E*****·Dy**_**2**_ (Figure 3a,b). This compares with a Zn···Zn distance of 8.45 Å in a closely related complex of the type ***f*****-P2·Zn**_**2**_.^25^ The distances of the Dy^III^ centers from the mean planes of the porphyrins (defined by the four nitrogen atoms) are similar to those in ***s*****-P2·Dy**_**2**_ (1.2169(6) and 1.2671(5) Å in ***f*****-P2-*****Z*****·Dy**_**2**_ and 1.2164(6) Å in ***f*****-P2-*****E*****·Dy**_**2**_). In the *syn* isomer ***f-*****P2-*****Z*****·Dy**_**2**_, the steric clashes between the two adjacent Kläui ligands result in a tilting of the magnetic centers (Figure 3a). These steric interactions are absent in ***f-*****P2-*****E*****·Dy**_**2**_, and the magnetic centers have an exactly antiparallel alignment. The angles between the Co–Dy vector and the mean plane or the four porphyrin nitrogen atoms are 87.79(15) and 82.42(15)° for the *syn* isomer and 85.8(2)° for the *anti* isomer.

### Absorption Spectra

**Figure 4:**
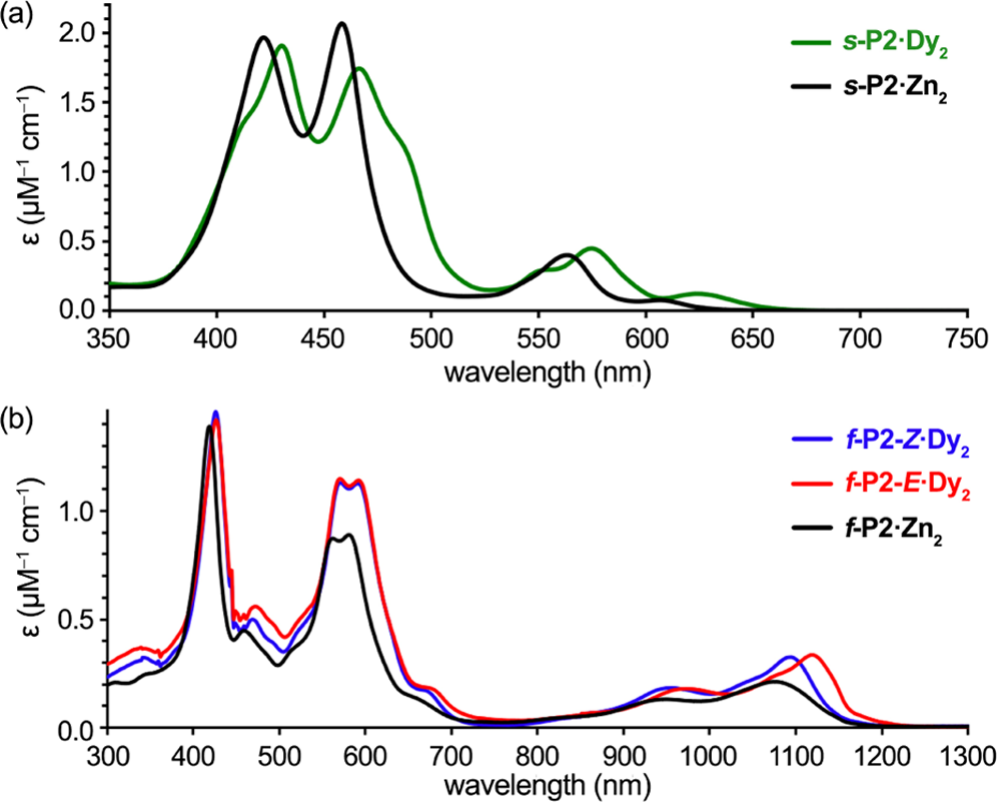
Absorption spectra of (a) the singly linked dimers ***s*****-P2·Dy**_**2**_ and ***s*****-P2·Zn**_**2**_ and (b) the edge-fused dimers ***f*****-P2*****-Z*****·Dy**_**2**_, ***f*****-P2*****-E*****·Dy**_**2**_, and ***f*****-P2·Zn**_**2**_. Spectra recorded in CHCl_3_ at 20 °C.

The absorption spectrum of ***s*****-P2·Dy**_**2**_ is compared with that of its Zn^II^ analogue, ***s*****-P2·Zn**_**2**_, in Figure 4a. The spectra are similar but that of ***s*****-P2·Dy**_**2**_ is bathochromically shifted, with the lowest energy band maximum of ***s*****-P2·Dy**_**2**_ at 625 vs 607 nm in ***s*****-P2·Zn**_**2**_. This shift may be attributed to the smaller porphyrin–porphyrin torsion angle in the Dy^III^ complex, as observed in the crystal structure, which allows more orbital overlap between the porphyrin π-systems.^26,27^

Edge-fused porphyrin dimers generally exhibit π–π* absorption bands extending to wavelengths of 1100 nm, reflecting strong π-conjugation.^13^ The absorption spectra of the *syn* and *anti* isomers ***f*****-P2-*****Z*****·Dy**_**2**_ and ***f-*****P2-*****E*****·Dy**_**2**_ are similar to that of the corresponding Zn^II^ complex (Figure 4b). The longest wavelength absorption band of the *anti* isomer, ***f*****-P2-*****E*****·Dy**_**2**_ (λ_max_ 1121 nm), is red-shifted relative to the *syn* isomer, ***f*****-P2-*****Z*****·Dy**_**2**_ (λ_max_ 1095 nm). In structurally related triply linked corrole dimers bearing Ga^III^, no difference in absorption behavior was observed between *syn* and *anti* isomers.^28^

### Redox Potentials

**Figure 5:**
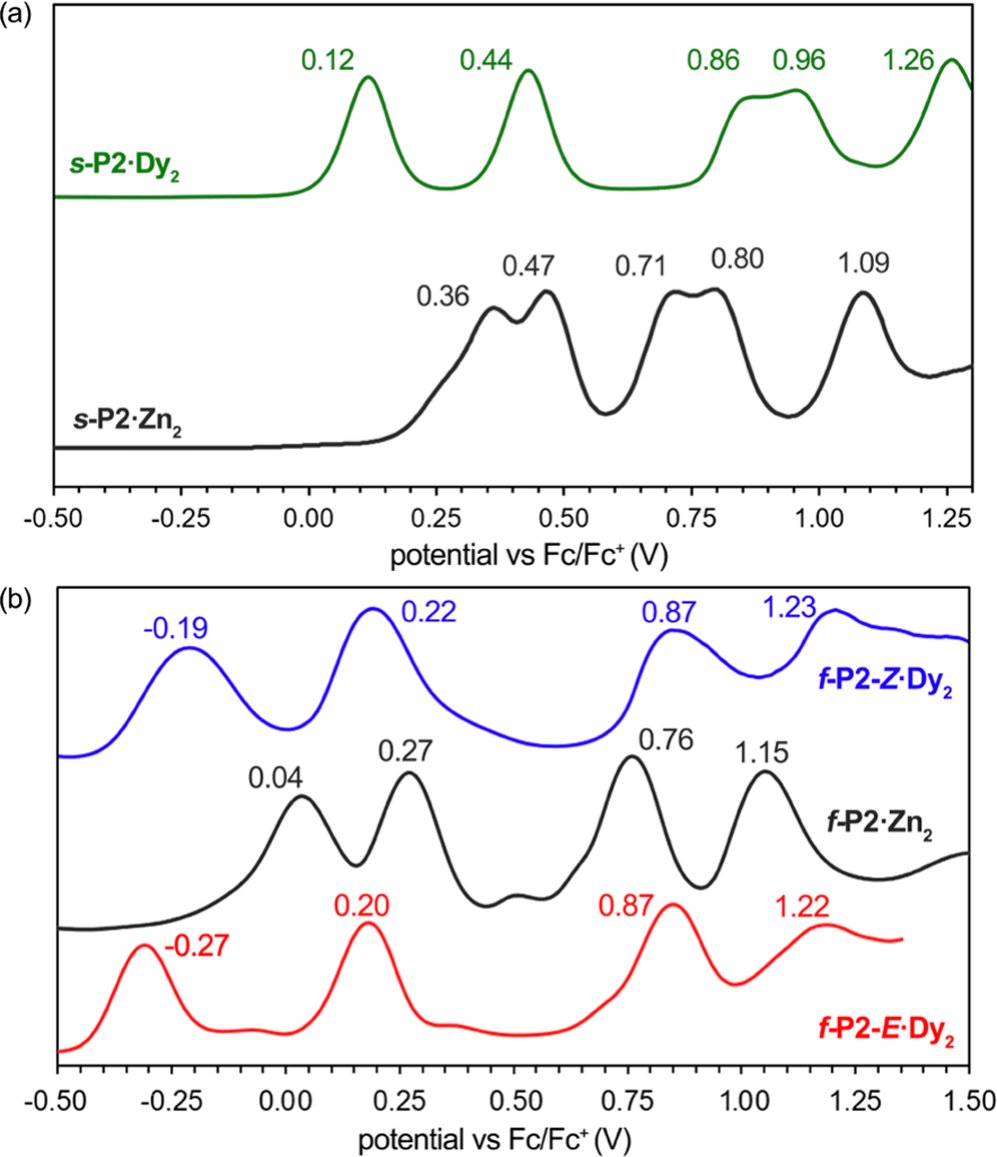
Differential pulse voltammograms of (a) ***s*****-P2·Dy**_**2**_ compared with those of ***s*****-P2·Zn**_**2**_, and (b) ***f*****-P2*****-Z*****·Dy**_**2**_ and ***f*****-P2**-***E*****·Dy**_**2**_ compared with those of ***f*****-P2·Zn**_**2**_. Solvent: CH_2_Cl_2_ containing 0.10 M NBu_4_PF_6_.

The differential pulse voltammograms of ***s*****-P2·Dy**_**2**_, ***f*****-P2*****-Z*****·Dy**_**2**_, and ***f*****-P2**-***E*****·Dy**_**2**_ are compared with those of ***s*****-P2·Zn**_**2**_ and ***f*****-P2·Zn**_**2**_ in Figure 5. The first oxidation of ***s-*****P2·Dy**_**2**_ (*E*_ox_ = 0.12 V vs Fc/Fc^+^) is noticeably easier than that of ***s-*****P2·Zn**_**2**_ (*E*_ox_ = 0.36 V), reflecting the fact that Dy^III^ is more electropositive than Zn^II^.^29^ Both ***s-*****P2·Dy**_**2**_ and ***s-*****P2·Zn**_**2**_ exhibit a total of five oxidation waves, and the potentials for the Dy^III^ complex are more widely spaced, so that the fifth oxidation potential of ***s-*****P2·Dy**_**2**_ (*E*_ox_ = 1.26 V) is substantially higher than that of ***s-*****P2·Zn**_**2**_ (*E*_ox_ = 1.09 V). Almost identical behavior was observed for ***s-*****P2·Gd**_**2**_ (see the Supporting Information, Figure S3).

The fused metalloporphyrin dimers ***f*****-P2·Ln**_**2**_ and ***f*****-P2·Zn**_**2**_ are easier to oxidize than the singly linked dimers, reflecting their smaller HOMO–LUMO gaps. Four oxidation processes are observed in the window of accessible potentials. As for ***s*****-P2·Dy**_**2**_, the lanthanide complexes are substantially easier to oxidize than ***f*****-P2·Zn**_**2**_. There is a significant shift in the first oxidation potential of ***f*****-P2*****-E*****·Dy**_**2**_ (−0.27 V) relative to that of ***f*****-P2*****-Z*****·Dy**_**2**_ (−0.19 V), indicating that the HOMO is higher in energy in the *anti* isomers. The other three oxidation potentials are almost the same for the *syn* and *anti* isomers. These data show that the inclusion of Ln^III^ metal centers does not disrupt the strong electronic coupling between adjoined porphyrins and that the metal geometry fine-tunes the underlying electronic structure.

The slightly higher HOMO and smaller optical HOMO–LUMO gap of the *anti* isomer could be attributed to the more regular molecular geometry of this isomer due to the absence of any clash between the capped metal centers, as observed in the crystal structure.

### Chiral Resolution and Circular Dichroism

**Figure 6:**
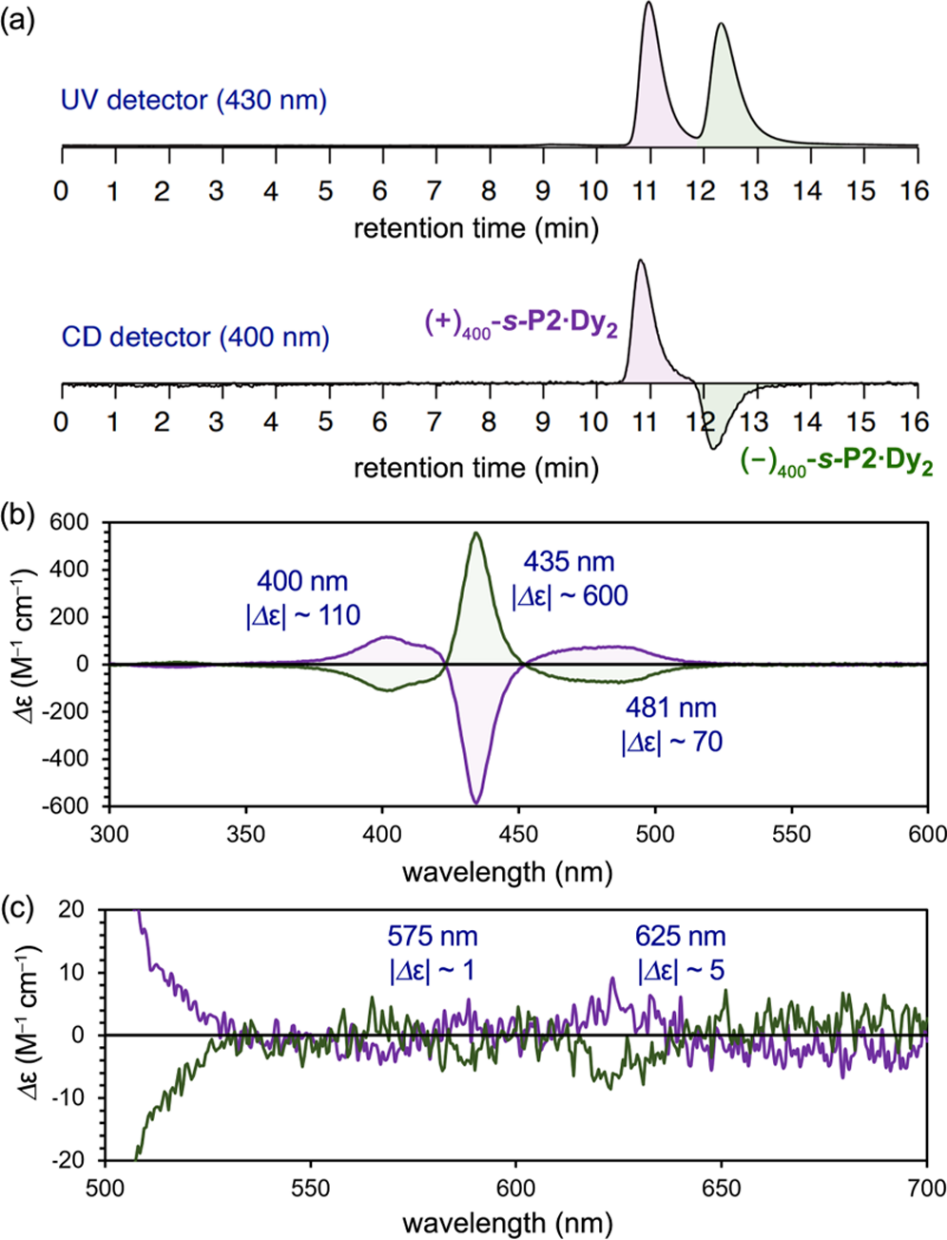
(a) Chiral HPLC trace of racemic ***s*****-P2·Dy**_**2**_ (stationary phase: SUMICHIRAL OA-2500; mobile phase: hexane/*i*-PrOH (99:1 v/v); flow rate: 1.0 mL min^–1^; temperature: 40 °C; and detector: 430 nm (top) and CD (bottom)). (b) Full and (c) expanded CD spectra (concentration 4 μM; solvent CHCl_3_; temperature: 25 °C).

Inserting Ln^III^ into singly linked porphyrin oligomers, with the capped Ln^III^ metal center outside the porphyrin plane, generates two enantiomers. The chirality of these compounds opens up possibilities for unusual magnetic behavior such as magnetochiral dichroism.^30^ While chiral singly linked porphyrin oligomers are well known, these structures are typically accessed through modification of pendant aryl groups or by preparing “strapped” porphyrins.^26,31−33^ To the best of our knowledge, this is the first example of metals acting as stereogenic elements in *meso*-linked porphyrin oligomers. We were pleased to find that enantiomers of ***s*****-P2·Dy**_**2**_ can be resolved via chiral HPLC using a SUMICHIRAL OA-2500 stationary phase, which is functionalized with (*R*)-1-naphthylglycine (Figure 6a). With each enantiomer in hand, circular dichroism (CD) spectra were recorded (Figure 6b). These complexes possess three major Cotton effects of opposite signs. The most intense signal is located at 435 nm (Δε ∼600 M^–1^ cm^–1^), with two less intense signals at 400 nm (Δε ∼110 M^–1^ cm^–1^) and 481 nm (Δε ∼70 M^–1^ cm^–1^). Very weak signals were also found at 575 nm (Δε ∼1 M^–1^ cm^–1^) and 625 nm (Δε ∼5 M^–1^ cm^–1^) (Figure 6c). Comparison of the CD spectra in Figure 6b with that of a closely related *meso–meso*-linked zinc porphyrin dimer^32^ suggests that (+)_400_-***s*****-P2·Dy**_**2**_ has the *S* configuration. This very tentative assignment assumes that the electronic transition dipole moments of ***s*****-P2·Dy**_**2**_ are similar to those of the zinc porphyrin derivative. A more definitive assignment of the absolute configurations would require a time-dependent density-functional theory (TD-DFT) analysis, which is difficult with dysprosium complexes or crystallographic analysis of the resolved material.

We tested whether it is possible to thermally racemize these enantiomers. The chiral HPLC trace of a solution of (+)_400_-***s*****-P2·Dy**_**2**_ remained unchanged after heating to 200 °C for 24 h in O_2_-free toluene in a sealed tube. There was no detectable racemization or decomposition, indicating that there is a high barrier to rotation about the central *meso–meso* single bond, as concluded previously for analogous zinc complexes.^31^

### EPR Spectroscopy and Quantum Coherence

We evaluated the quantum coherence properties of the Gd^III^ complexes by pulsed electron paramagnetic resonance (EPR) techniques to explore whether the compounds could be suitable for quantum information processing at low temperatures. These experiments yield key parameters such as the spin–lattice relaxation time, *T*_1_, and the spin–spin dephasing (or phase-memory) time, *T*_m_. Pulsed EPR techniques can also provide valuable information on weak spin–spin dipolar and exchange interactions in dimeric systems. We restrict our analysis to the Gd^III^ complexes because of the extreme broadening and zero-field splitting of Dy^III^ complexes. The orbital momentum is zero (*L* = 0) for ground-state Gd^III^ systems, so they can be treated as pure spin systems with a total spin of *S* = 7/2. The electrostatic crystal-field environment splits the ground state into four Kramers doublets with |*m*_s_⟩ = |±1/2⟩, |±3/2⟩, |±5/2⟩, and |±7/2⟩. Typically, for Gd^III^, these splittings are relatively small (∼100 GHz), so that all of these states are populated at liquid helium temperatures. Furthermore, for Gd^III^ complexes, mixing between these states due to the crystal field can be neglected and we can consider them as pure doublets. For these reasons, the quantum coherence properties are much more pronounced in Gd^III^ ions, rather than in Dy^III^.^11,34^

**Figure 7:**
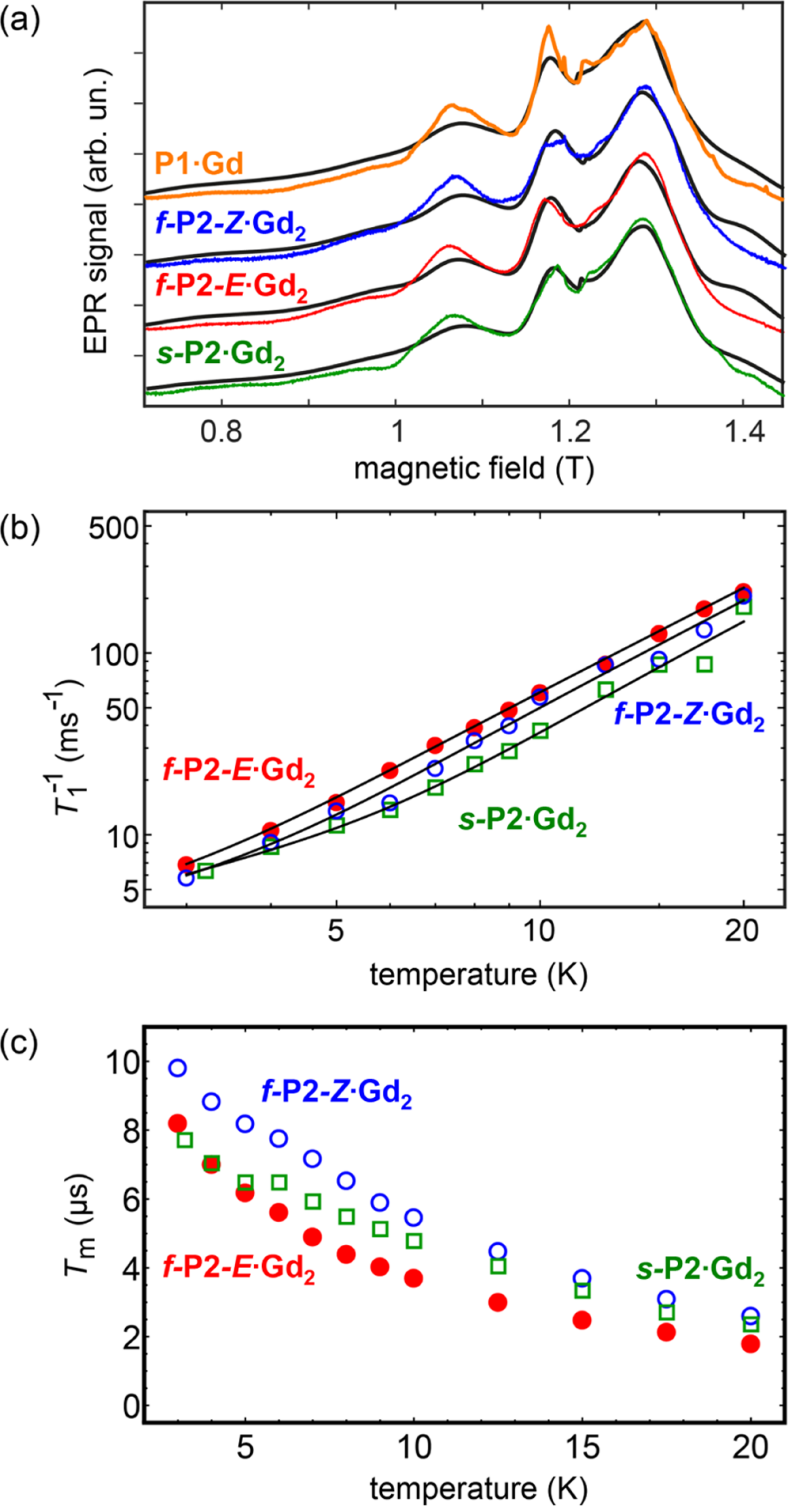
(a) Echo-detected field-swept Q-band EPR spectra for **P1·Gd** (orange), ***f*****-P2-*****Z*****·Gd**_**2**_ (blue), ***f*****-P2-*****E*****·Gd**_**2**_ (red), and ***s*****-P2·Gd**_**2**_ (green) acquired at 5 K. Black lines are fits to the data based on the spin Hamiltonian (see the SI). (b) Temperature dependence of the spin-phonon relaxation rate *T*_1_^–1^ for ***f*****-P2-*****E*****·Gd**_**2**_ (red, full dots), ***f*****-P2-*****Z*****·Gd**_**2**_ (blue, open dots), and ***s*****-P2·Gd**_**2**_ (green, open rectangles), acquired at *B* = 1.289, 1.287, and 1.285 T, respectively. Black lines are fits to the data to eq 1. (c) Temperature dependence of the phase-memory time *T*_m_ for ***f*****-P2-*****E*****·Gd**_**2**_ (red, full dots), ***f*****-P2-*****Z*****·Gd**_**2**_ (blue, open dots), and ***s*****-P2·Gd**_**2**_ (green, open rectangles), acquired at *B* = 1.289, 1.287, and 1.285 T, respectively.

The EPR spectra of **P1·Gd**, ***f*****-P2-*****Z*****·Gd**_**2**_, ***f*****-P2-*****E*****·Gd**_**2**_, and ***s*****-P2·Gd**_**2**_ were recorded as 1 mM solutions in CS_2_, at temperatures of 3–20 K, using an echo-detection technique. This solvent forms a glassy matrix below 160 K. Spectra recorded at 5 K are shown in Figure 7a. Normally, Gd^III^ complexes show a narrow, intense spectral feature originating from the |−1/2⟩ → |+1/2⟩ transition.^35^ However, the Kläui ligands seem to induce an unusually strong crystal field, and we found a strong zero-field splitting. Spectral calculations (see Figure S42) show that, for randomly oriented molecules as in a frozen solution, the different peaks cannot be clearly assigned to the magnetic states. For example, the |−1/2⟩ → |+1/2⟩ transition varies between 1.16 and 1.33 T due to its orientation dependence, giving rise to two large peaks. However, the field-orientation dependence of other allowed transitions is significantly stronger due to their larger magnetic state, thus overlapping with those transitions. However, the |−1/2⟩ → |+1/2⟩ is known to display a narrow line width when the molecular quantization axis is aligned with the magnetic field. This shows as a kink in the data around 1.22 T for a given frequency of 33.85 GHz. Differences observed in the spectra of ***f*****-P2-*****Z*****·Gd**_**2**_, ***f*****-P2-*****E*****·Gd**_**2**_, and ***s*****-P2·Gd**_**2**_ can be explained by slight differences of the crystal-field parameters. All of the spectra could be fitted using a model that accounts for the crystal-field splitting parameters *D* and *E* and associated strains, as well as an isotropic *g*-factor and exchange coupling *J* mediated by the bridging ligands (see the SI, Table S7). The crystal-field splitting and the strong orientation dependence dominate the shapes of the spectra, and, consequently, the coupling strengths deduced from fitting these spectra have large error bars. *D* and *E* are similar for all three Gd complexes, with values between −3518 and −3590 MHz for *D* and between 249 and 304 MHz for *E* (see Table S7). In the fused dimers, the exchange coupling between the spins is very weak and antiferromagnetic. Fitting the spectra gives *J* = −21 and *J* = −24 MHz for ***f*****-P2-*****Z*****·Gd**_**2**_ and ***f*****-P2-*****E*****·Gd**_**2**_, respectively, but these values must be regarded as very approximate. More accurate values of the exchange coupling in these compounds were estimated from the low-temperature static magnetic susceptibility data, as discussed below. For the singly linked dimer ***s*****-P2·Gd**_**2**_, exchange coupling was not detected, as expected, owing to the negligible orbital overlap of the two porphyrin conjugated systems.

The spin–lattice relaxation rate, 1/*T*_1_ shows a rather linear increase with temperature below 6 K (Figure 7b). At temperatures above 6 K, 1/*T*_1_ follows a polynomial law *T*^*n*^ with *n* ≫ 1, typical for Raman processes. Fitting with a combination of a direct and a Raman relaxation process could be performed,^36^ showing good agreement with eq 1 from 3 to 20 K (see the SI)
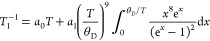
1Here, *a*_0_ corresponds to the direct relaxation rate constant, *a*_1_ corresponds to the Raman relaxation rate constant, θ_D_ corresponds to the Debye temperature, and *T* corresponds to the temperature. We found that *a*_0_ = 1.90(27) K^–1^ s^–1^, *a*_1_ = 1503(834) s^–1^, θ_D_ = 22.5(55) K for ***f-*****P2*****-Z·*****Gd**_**2**_; *a*_0_ = 2.07(19) s^–1^, *a*_1_ = 1118(353) s^–1^, θ_D_ = 18.0(27) K for ***f-*****P2*****-E·*****Gd**_**2**_; and *a*_0_ = 2.01(17) s^–1^, *a*_1_ = 2627(1423) s^–1^, θ_D_ = 33.9(71) K for ***s-*****P2*****·*****Gd**_**2**_. Below 6 K, a slight deviation is observed, particularly for ***f-*****P2*****-E·*****Gd**_**2**_, as is typical for direct processes, in agreement with the processes limiting the ac spin dynamics of the Dy^III^ analogues (see below).

The phase-memory times, *T*_m_, were measured using a Hahn-echo sequence (see the SI). Fitting the signal decay was performed with a monoexponential decay function. The *T*_m_ times increase sharply on lowering *T*, until, at 3 K, they reach up to 9.8 μs for ***f*****-P2-*****Z*****·Gd**_**2**_ and 8.2 μs for ***f*****-P2-*****E*****·Gd**_**2**_ (Figure 7c), which is sufficiently long to test the quantum computational schemes using microwave pulses. The *T*_m_ times for ***s*****-P2·Gd**_**2**_ lie in between those of the fused dimers. *T*_m_ slightly depends on the applied magnetic field *B* because different states and transitions can be selected and probed (see the SI). In the whole region, we are still far below the coherence time limit (i.e., *T*_2_ ≪ 2*T*_1_, where *T*_2_ is the quantum coherence time, a major contributing factor to *T*_m_), indicating that hyperfine interactions with ^155,157^Gd, ^14,15^N, and ^31^P probably dominate the decoherence process. Even in this limit, the complex would allow ca. 250 two-quantum-bit operations to be performed within the time constrictions imposed by the spin–spin interactions. Interestingly, although the spin–spin interactions of the *syn* and *anti* complexes are very similar, the different symmetries introduced by the two configurations have an effect on the quantum coherence. In the whole temperature range examined, ***f*****-P2-*****Z*****·Gd**_**2**_ always displays substantially longer coherence than ***f*****-P2-*****E*****·Gd**_**2**_ (up to 30% longer). Intermolecular interactions can be ruled out since the molecules are spaced far enough apart in a 1 mM frozen glassy solution. The effect is noteworthy, as several proposals rely on slightly tilted neighboring spins for two-qubit operations.^37^

### Static Magnetic Properties

**Figure 8:**
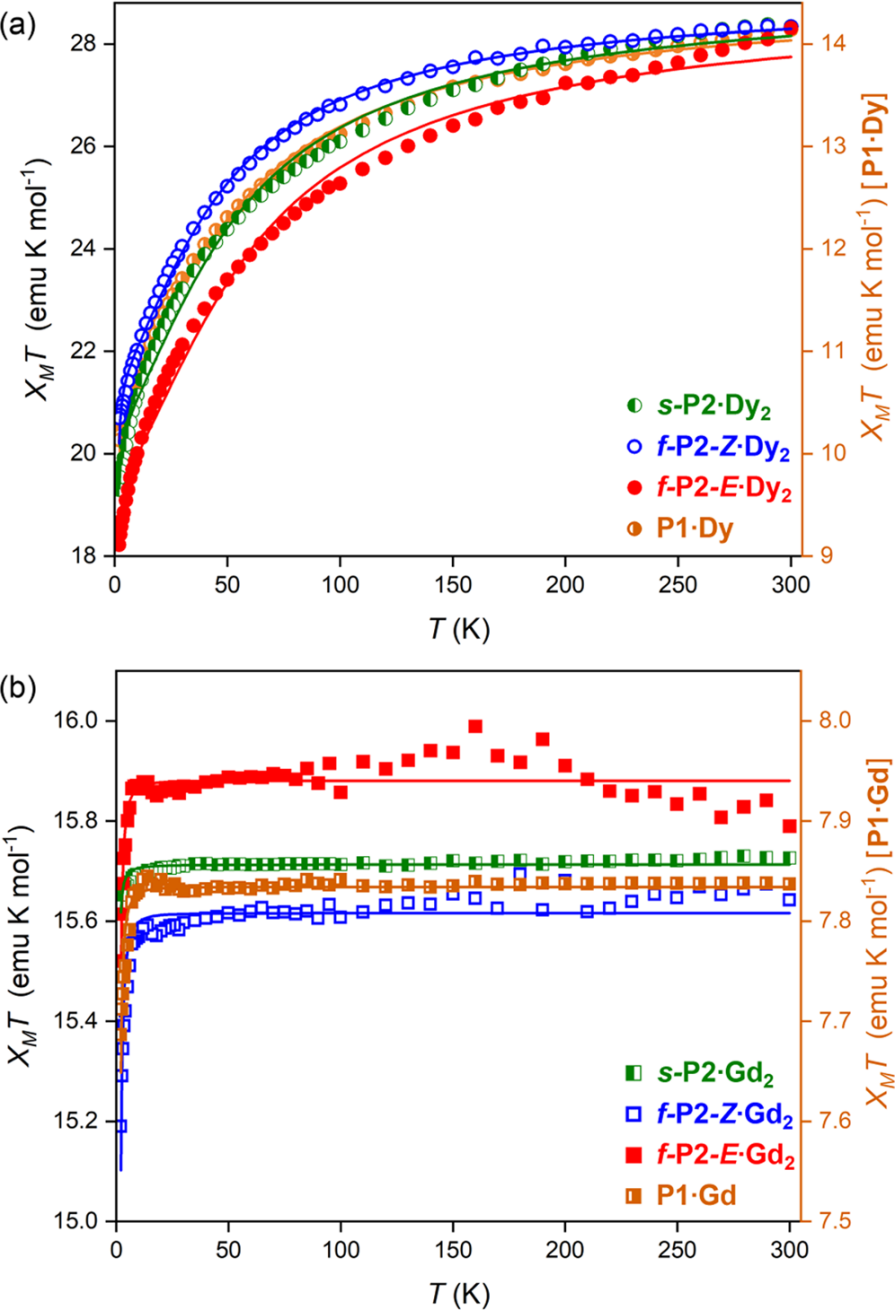
Temperature dependence of the static magnetic susceptibility for complexes ***s*****-P2·Dy**_**2**_, ***f*****-P2-*****Z*****·Dy**_**2**_, ***f*****-P2-*****E*****·Dy**_**2**_, ***s*****-P2·Gd**_**2**_, ***f-*****P2-*****Z*****·Gd**_**2**_, and ***f*****-P2-*****E*****·Gd**_**2**_ and for their respective monomers **P1·Dy** and **P1·Gd**. All curves acquired in a static field *B* = 0.1 T. Solid lines are fits.

The variable-temperature magnetic properties of all of the complexes were determined using an MPMS-XL SQUID magnetometer. The dependence of the static magnetic susceptibility, χ_M_, on temperature *T*, is shown in Figure 8 for all compounds: **P1·Dy**, ***s*****-P2·Dy**_**2**_, ***f*****-P2-*****Z*****·Dy**_**2**_, ***f*****-P2-*****E*****·Dy**_**2**_, **P1·Gd**, ***s*****-P2·Gd**_**2**_, ***f*****-P2-*****Z*****·Gd**_**2**_, and ***f*****-P2-*****E*****·Gd**_**2**_, where χ_M_ is the ratio between the magnetization *M* and the applied external magnetic field *B*. Dy^III^ ions have a ^6^H_15/2_ ground-state configuration, and very large spin–orbit coupling leads to the presence of several Kramers doublets that are split by anisotropy, while Gd^III^ ions are in the ^8^S_7/2_ configuration and thus lack any spin–orbit contribution. The χ_M_*T* values at 300 K for the monomers (Table 1) agree with the expected values for a single Dy^III^ or Gd^III^ ion (14.2 and 7.9 emu K mol^–1^, respectively),^38^ and the values for the dimers are close to twice those of the monomers, as expected for two noninteracting ions. In all of the Dy compounds, χ_M_*T* decreases slightly on cooling, with a steep decrease below 100 K (Figure 8a). This decrease is mainly linked to the depopulation of the excited Stark sublevels of the Dy^III^, as revealed by comparing the curves of the dimers to twice that of the monomer.^39^ For **P1·Gd**, χ_M_*T* remains constant from 300 to 16 K and then decreases slightly to 7.7(2) emu K mol^–1^ (Figure 8b). For the Gd_2_ dimers, χ_M_*T* also remains almost constant until 16 K; below this temperature, χ_M_*T* decreases to reach values of 15.6(4) emu K mol^–1^ for ***s*****-P2·Gd**_**2**_, 15.2(5) emu K mol^–1^ for ***f*****-P2-*****Z*****·Gd**_**2**_, and 15.5(5) emu K mol^–1^ for ***f*****-P2-*****E*****·Gd**_**2**_ at *T* = 2 K. This low-*T* decrease suggests the presence of weak intramolecular antiferromagnetic interactions. As the monomer curves always lie between the curves of the *syn* and *anti* isomers, it is tempting to attribute ferromagnetic interactions to ***f*****-P2-*****Z*****·Dy**_**2**_ and antiferromagnetic ones to ***f*****-P2-*****E*****·Dy**_**2**_. On the other hand, the EPR analysis indicates that the interactions are in the range of −21 to −24 MHz, and the effect should rather be attributed to changes in the anisotropy, as produced by the considerable distortion of the coordination environment in ***f*****-P2-*****Z*****·Dy**_**2**_.

**Table 1:** Static Magnetic Susceptibilities Measured at 300 K

complex	χ_M_*T*/emu K mol^–1^
**P1·Dy**	14.1(4)
***s-*****P2·Dy**_**2**_	28.3(4)
***f*****-P2-*****Z*****·Dy**_**2**_	28.3(8)
***f*****-P2-*****E*****·Dy**_**2**_	28.3(8)
**P1·Gd**	7.8(2)
***s-*****P2·Gd**_**2**_	15.7(4)
***f*****-P2-*****Z*****·Gd**_**2**_	15.8(5)
***f*****-P2-*****E*****·Gd**_**2**_	15.6(5)

Magnetization curves were recorded for all complexes at 2, 5, and 7 K up to 7 T (Figures S7–S14). The *M* vs *B* curves show a rapid increase at low fields (below 1 T) for all Dy^III^ complexes, followed by a slow, almost linear increase at high fields. For Dy^III^ complexes, the *M* vs *B* curves agree well with the simulations that include the presence of magnetic anisotropy. In the case of the isotropic complexes ***s*****-P2·Gd**_**2**_, ***f*****-P2-*****Z*****·Gd**_**2**_, and ***f*****-P2-*****E*****·Gd**_**2**_ (Figures S12–S14), even at 2 K, the magnetization shows a rapid increase and reaches a saturation value expected for noninteracting Gd^III^ ions, in agreement with the very weak interactions detected by EPR.^36^

For **P1·Gd**, ***s-*****P2·Gd**_**2**_, ***f*****-P2-*****Z*****·Gd**_**2**_, and ***f*****-P2-*****E*****·Gd**_**2**_, the magnetic data were fitted using a model that accounts for the isotropic *g*-factor, an isotropic exchange coupling parameter *J* for the dimers, and the zero-field splitting parameters *D* and *E*, as determined from EPR (Table S2). The inclusion of *J* improves the fit quality (Figure 8b). We found values of *g* = 1.9942(55) for **P1·Gd**, *g* = 1.9980(201) and *J* = 0 MHz for ***s-*****P2·Gd**_**2**_, *g* = 1.9919(127) and *J* = (−51 ± 2) MHz for ***f*****-P2-*****Z*****·Gd**_**2**_, and *g* = 2.0082(382) and *J* = (−19 ± 3) MHz for ***f*****-P2-*****E*****·Gd**_**2**_. The *J* values of the fused dimers come close to the EPR results and indicate a small antiferromagnetic exchange. These interactions fall in the useful range for two-quantum-bit operations, which for the values above could be performed at 40 ns. These values are thus encouraging for the perspective use of porphyrin scaffolds and would allow 30 times the operations of previously proposed bimetallic complexes.^40^ Best fits for the singly linked dimer are obtained when neglecting exchange interactions. Data of the Dy analogues were fitted using a simplified ligand-field model considering only second-order zero-field-splitting parameters, but overparameterization limits the reliability in determining the exchange. We found good agreement using *S* = 15/2 with *g* = 1.3449(63), *D* = (−510.0 ± 0.4) GHz, and *E* = (603.9 ± 0.5) MHz for **P1·Dy**; *g* = 1.3484(96), *D* = (−558.0 ± 46.2) GHz, and *E* = (645.2 ± 51.7) GHz for ***s*****-P2·Dy**_**2**_; *g* = 1.3457(233), *D* = (−531.2 ± 156.2) GHz, and *E* = (449.5 ± 30.7) GHz for ***f*****-P2-*****Z*****·Dy**_**2**_; and *g* = 1.3445(83), *D* = (−870.1 ± 145.4) GHz, and *E* = (779.7 ± 36.7) GHz for ***f*****-P2-*****E*****·Dy**_**2**_.

### Dynamic Susceptibilities

**Figure 9:**
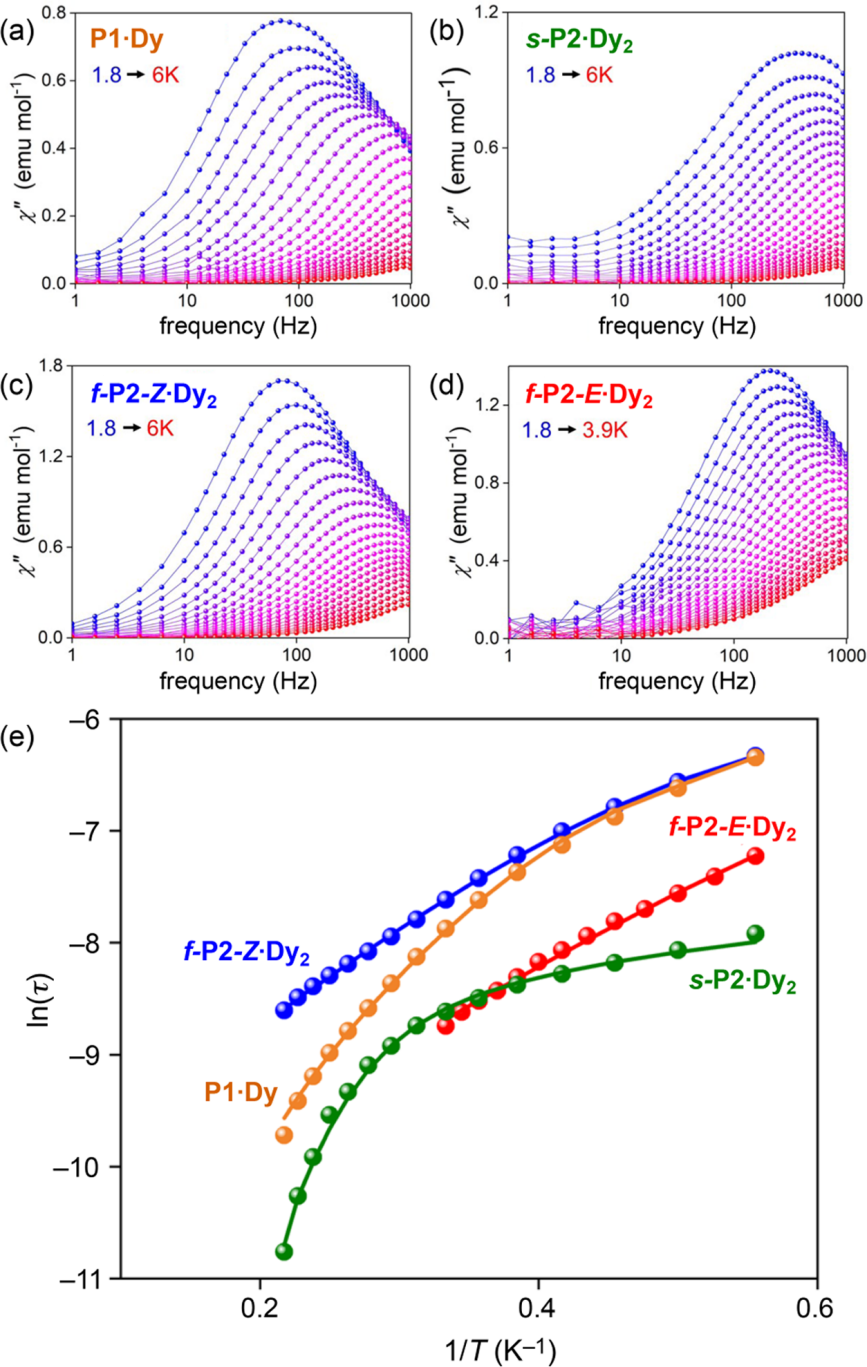
Frequency dependence of the out-of-phase signal of (a) **P1·Dy**, (b) ***s*****-P2·Dy**_**2**_, (c) ***f*****-P2-*****Z*****·Dy**_**2**_, and (d) ***f*****-P2-*****E*****·Dy**_**2**_; solid lines are guides for the eye. (e) Comparison of the Arrhenius plots of **P1·Dy**, ***s*****-P2·Dy**_**2**_, ***f*****-P2-*****Z*****·Dy**_**2**_, and ***f*****-P2-*****E*****·Dy**_**2**_. Solid lines highlight fits to a modified Arrhenius equation including quantum tunneling of the magnetization (see the text).

Alternating current (ac) magnetic susceptibility measurements were performed to probe the dynamics of the anisotropic compounds, **P1·Dy**, ***s*****-P2·Dy**_**2**_, ***f*****-P2-*****Z*****·Dy**_**2**_, and ***f*****-P2-*****E*****·Dy**_**2**_, and to check for slow relaxation of magnetization. We used a 0.2 mT oscillating field at variable frequencies ν = 1–1000 Hz. None of the complexes exhibit in-phase (χ′) or out-of-phase (χ″) susceptibility signals at zero static field between 2 and 20 K (Figures S15–S17). In the Dy^III^ complexes, this is typical of significant quantum tunneling (QT) of the magnetization. To suppress this QT behavior, ac susceptibility measurements were performed as a function of *B*, showing a well-resolved maximum in χ″ at *B* = 0.12 T (Figures S19–S34). At this field, all Dy complexes show the presence of peaks that shift to lower ν on decreasing *T*, as indicative of the slow relaxation of the magnetization, or single-molecule-magnet behavior, produced by the presence of a magnetic anisotropy barrier that hinders the reversal of the spin at the single-molecular level (Figure 9).

The Argand plots display semicircular shapes that fit to a generalized Debye model for **P1·Dy**, ***s*****-P2·Dy**_**2**_, ***f*****-P2-*****Z*****·Dy**_**2**_, and ***f*****-P2-*****E*****·Dy**_**2**_ (Figures S25, S28, S31, and S34).^41^ The model includes an α parameter that accounts for a possible distribution of relaxation times (τ) and which is found at all temperatures and, for all of the Dy complexes (Tables S3–S6), to be in the range of 0.1–0.3, as compatible with single molecule behavior. The Arrhenius plots could be fitted with an Arrhenius law modified to include QT rate τ_QT_^–1^, a Raman process *CT*^*n*^, in addition to the Orbach relaxation channel
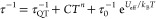
2where *U*_eff_ is the relaxation energy barrier, τ_0_ is a pre-exponential factor, *k*_B_ is the Boltzmann constant, *C* is a parameter containing the spin-phonon coupling matrix element, and *n* is the standard Raman exponent and is expected to be 9 for Kramers ions, or 5 in the presence of low-lying states.^42^ Best-fit parameters are shown in Table 2.

**Table 2:** Fitting Parameters for Compounds **P1·Dy**, ***s*-P2·Dy_2_**, ***f*-P2-*Z*·Dy_2_**, and ***f*-P2-*E*·Dy_2_** Relating to eq 2

	P1·Dy	*s-*P2·Dy_2_	*f-*P2-*Z*·Dy_2_	*f-*P2-*E*·Dy_2_
τ_QT_/s	3.9(33) × 10^–3^	3.5(5) × 10^–4^	3.4(7) × 10^–3^	1.1(5) × 10^–3^
*n*	9	9	9	9
*C*/s^–1^ K^–*n*^	9.7(5) × 10^–3^	2.23(24) × 10^–2^	2.5(15) × 10^–3^	8.9(7) × 10^–3^
τ_0_/s	1.7(5) × 10^–6^	1.44(1) × 10^–5^	3.0(3) × 10^–6^	6.6(34) × 10^–6^
*U*_eff_/K	9.8(12)	10.4(10)	8.9(4)	10.1(17)

This analysis reveals that bimetallic complexes ***s-*****P2·Dy**_**2**_, ***f-*****P2-*****Z*****·Dy**_**2**_, and ***f-*****P2-*****E*****·Dy**_**2**_ show similar activation dynamics. The relaxation barriers for ***f-*****P2-*****Z*****·Dy**_**2**_ and ***f-*****P2-*****E*****·Dy**_**2**_ are comparable with the monometallic complex **P1·Dy**, slightly higher than those of the butadiyne-linked Dy_2_ porphyrin dimers,^11^ and similar to that reported for the 10,15,20-tetraphenylporphyrin dysprosium complex.^20^ The τ_QT_ values suggest that quantum tunneling of the magnetization dominates in the low-temperature regime for all of the complexes, although it is reduced to some extent by the application of *B*. For all complexes, the fitted model agrees excellently with the prediction for Kramers ions.^43^

### Low-Temperature Magnetic Anisotropy

**Figure 10:**
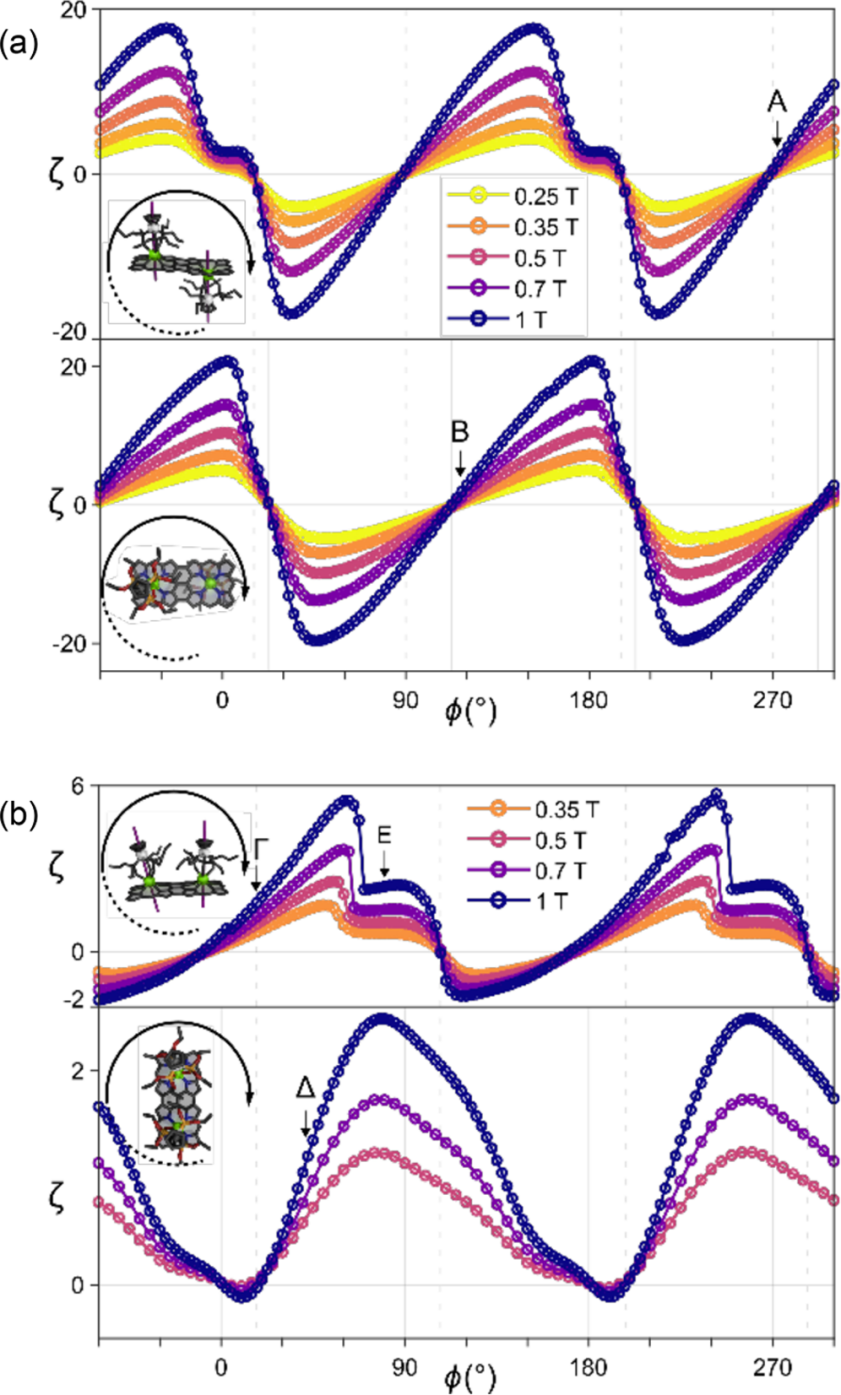
(a) Angular dependence of the magnetic cantilever-torque signal ζ of ***f*****-P2-*****E*****·Dy**_**2**_ measured at 50 mK at different fields (color scale common to both panels). The two panels depict two rotations acquired for two different orientations of the crystal. The directions of the rotation with respect to the molecular orientation are depicted in the insets. Symbols denote the main angle for which the hysteresis loops are shown in Figure 11. (b) Angular dependence of the magnetic torque signal ζ of ***f*****-P2-*****Z*****·Dy**_**2**_ measured at 50 mK at different fields (color scale common to both panels). The two panels depict two rotations acquired for two different orientations of the crystal. The direction of the rotation with respect to the molecular orientation is depicted in the insets. Symbols denote the main angle for which the hysteresis loops are shown in Figure 11.

The largest effects of the symmetry changes introduced by the aromatic plane are likely to arise in the magnetic anisotropy. The in-built magnetic anisotropy of the molecule will lead to a preferential orientation of the magnetization along the anisotropy axis and will thus give rise to a magnetic torque, **ζ** = **M** × **B**, which will tend to move the crystal to align **M** along **B**. A complete characterization of the torque response as a function of the orientation of **B** and *T* was thus performed on both ***f*****-P2-*****Z*****·Dy**_**2**_ and ***f*****-P2-*****E*****·Dy**_**2**_ at milli-kelvin temperatures, as shown in Figure 10. The torque signal is measured via the deflection of a 50 μm thick CuBe cantilever, induced by a magnetic field *B*, and measured as the variation of the capacitance with an underlying conductive plate (SI). Single crystals of both isomers were measured in two different crystal orientations each: in a plane approximately perpendicular and parallel to the porphyrin plane (Figure 10). Whatever the orientation, ***f*****-P2-*****E*****·Dy**_**2**_ displays the torque behavior characteristic of a paramagnet with 180° periodicity and approximately the same magnitude of positive and negative torques at extremal points. ***f*****-P2-*****Z*****·Dy**_**2**_ displays a torque signal that is almost always positive and is distinguished by a region of almost 180° without any torque inversion.

Both ***f*****-P2-*****Z*****·Dy**_**2**_ and ***f*****-P2-*****E*****·Dy**_**2**_ crystallize with two molecules per unit cell (*Z* = 2), but a crystallographic inversion center is present, so that the two molecules are equivalent. Moreover, the intramolecular inversion center in ***f*****-P2-*****E*****·Dy**_**2**_ makes the two Dy^III^ magnetic centers equivalent, and their anisotropy axes must be collinear. Therefore, ***f*****-P2-*****E*****·Dy**_**2**_ contains only one type of center, with all of the anisotropy axes exactly aligned. For the purposes of torque magnetometry, the crystal response is thus equivalent to a single Dy^III^-porphyrin building block. In contrast, the ***f*****-P2-*****Z*****·Dy**_**2**_ complex lacks the intramolecular inversion center, and the overall molecular anisotropy is thus the sum of the two noncollinear anisotropies at the Dy^III^ sites. This key difference causes a stark difference in the observed torque. While ***f*****-P2-*****E*****·Dy**_**2**_ displays a periodic torque signal, centered around ζ = 0, the torque of ***f*****-P2-*****Z*****·Dy**_**2**_ complex is substantially shifted toward positive values. Physically, the former behavior is typical of a paramagnetic system, while the latter is possible only for a blocked system that is allowed to reverse through QT at certain ϕ. This is indeed shown by the permanent magnetization exhibited by the complex, which shows QT effects at 20 and 200° (Figure 10). This indicates that the aromatic plane and the symmetry-breaking can influence dramatically the SMM behavior, leading to different quantum selection rules for QT.

**Figure 11:**
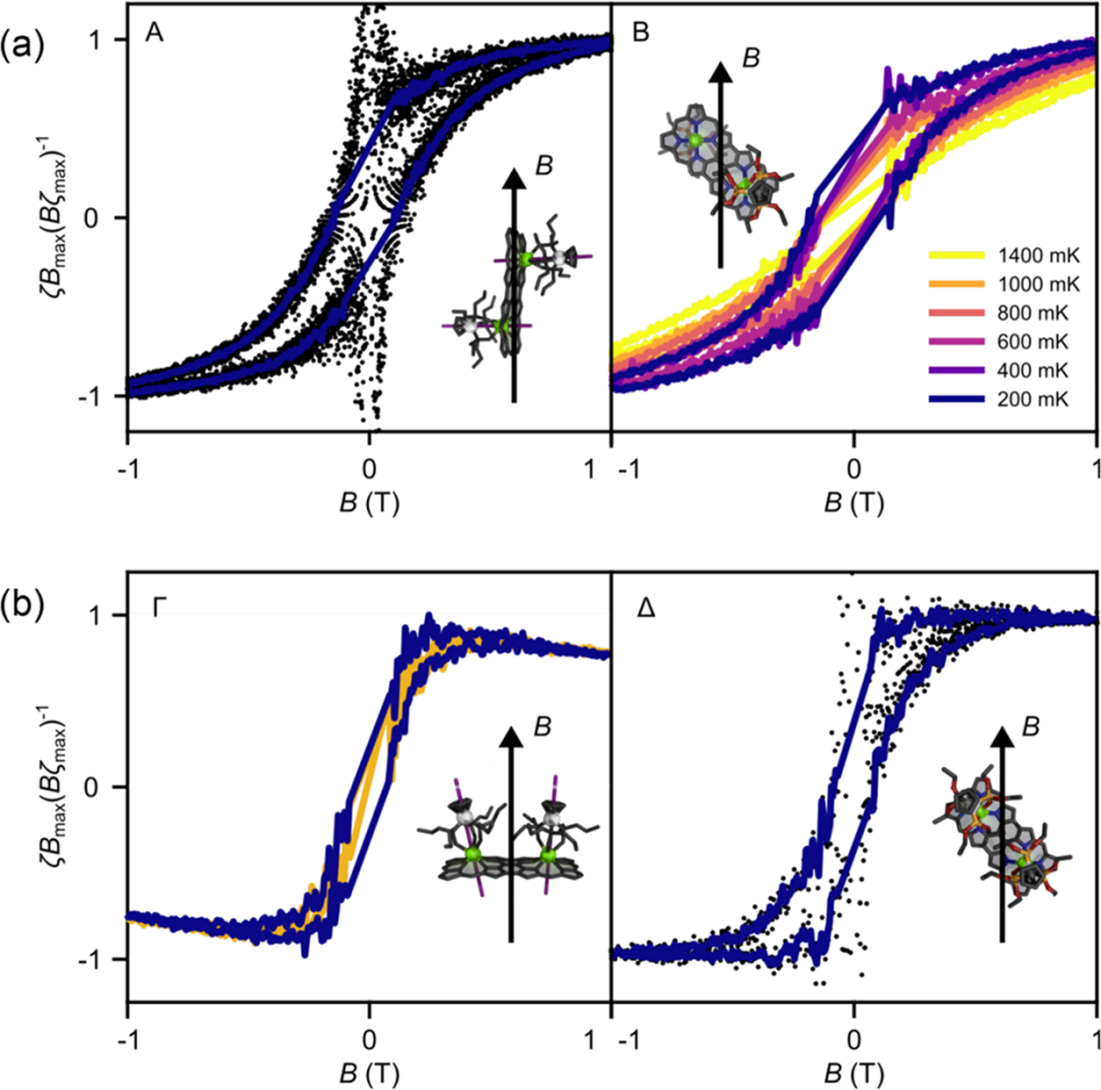
(a) Molecular hysteresis detected via torque magnetometry on single crystals of ***f*****-P2-*****E*****·Dy**_**2**_. Black dots are measured data, and solid lines are the averaging over multiple *B* sweeps. Symbols denote the orientations, as indicated in Figure 10. (b) Molecular hysteresis detected via torque magnetometry on single crystals of ***f*****-P2-*****Z*****·Dy**_**2**_. Black dots are measured data, and solid lines are the averaging over multiple *B* sweeps at different *T* where blue lines are acquired at 0.2 K. All measurements are acquired sweeping the external magnetic field *B* at 0.125 T/min. Temperature is indicated in the color scale, common to all panels. The direction of *B* with respect to the molecular orientation is depicted in the insets. Symbols denote the orientations, as indicated in Figure 10.

To investigate the SMM behavior, we measured the dependence of the torque while sweeping *B* for different orientations (Figure 11). Both compounds, ***f*****-P2-*****E*****·Dy**_**2**_ and ***f*****-P2-*****Z*****·Dy**_**2**_, show the opening of a hysteresis cycle below 500 mK, with the cycles becoming wider at lower *T*, as is typical of the slow magnetization dynamics resulting from SMM behavior (Figure 11a,b). The hysteresis loop is considerably wider for ***f-*****P2-*****E*****·Dy**_**2**_, although strong zero-field QT is still visible down to 50 mK. This is consistent with the presence of relatively strong Dy–Dy interactions in the compound, i.e., with a relaxation process that involves both Dy centers at the same time and no spin-exchange bias at low field. The collinear anisotropy of ***f*****-P2-*****E*****·Dy**_**2**_ quenches the QT of magnetic moment through transverse anisotropy terms that produces large hysteresis (Figure 11a). On the other hand, the noncollinear anisotropies of ***f*****-P2-*****Z*****·Dy**_**2**_ allow the overlapping of transverse anisotropy terms and thereby increase QT probability, which, in turn, decreases the observed hysteresis (Figure 11b).

## Conclusions

The synthetic chemistry presented here establishes fused porphyrin oligomers bearing paramagnetic metal centers as prime compounds for investigating spin effects in π-conjugated nanostructures. Our results demonstrate that it is possible to obtain a perfect definition of the π-plane–spin system: we reveal that lanthanide complexes of a singly linked porphyrin dimer, ***s*****-P2·Ln**_**2**_, can be resolved into enantiomers and that the corresponding complexes of an edge-fused porphyrin dimer can be separated into *syn* and *anti* diastereomers, ***f*****-P2-*****Z*****·Ln**_**2**_ and ***f*****-P2-*****E*****·Ln**_**2**_. In this way, the optical, electronic, electrochemical, and magnetic properties of all of the possible conformations of dinuclear Dy^III^ and Gd^III^ complexes can be compared and analyzed. The comparison between two types of dimers reveals that large differences in π-conjugation, which are strongly expressed in their absorption spectra and redox potentials, have less dramatic effects on the magnetic properties. Complete π-conjugation in the fused dimers results in comparatively strong magnetic exchange coupling between the metal centers. The static magnetic susceptibilities of the complexes at 300 K all match the expected values for isolated Gd^III^ and Dy^III^ ions, whereas the low-temperature magnetic susceptibility data reveal sizable differences in the exchange coupling of the dimers. The precise determination of interactions using EPR yields *syn* couplings twice as large as those for the *anti* isomer (*J* = −51 ± 2 MHz in ***f*****-P2-*****Z*****·Gd**_**2**_ and *J* = −19 ± 3 MHz in ***f*****-P2-*****E*****·Gd**_**2**_), with vanishingly small exchange transmitted when conjugation is blocked in ***s*****-P2·Gd**_**2**_.

The dynamic magnetic properties also reveal dramatic effects of the conformation around the π-conjugated plane. All of the Dy^III^ complexes show similar activation behavior characteristic of a single-molecule magnet. This confirms that the presence of a π-conjugated plane is not enough to perturb the large axial terms of the single-ion anisotropy of Dy^III^. On the other hand, quantum tunneling and hysteresis cycles are much more sensitive to small perturbations of the transverse terms, as revealed by the torque in magnetic anisotropy at 50 mK. The difference in symmetry between *syn* and *anti* isomers in ***f*****-P2-*****Z*****·Ln**_**2**_ and ***f*****-P2-*****E*****·Ln**_**2**_ leads to stark differences in the observed hysteresis, with narrower hysteresis and efficient tunneling in ***f*****-P2-*****E*****·Ln**_**2**_.

A delocalized π-conjugated pathway is a key component for electronic devices and quantum processing. Our results reveal that rare-earth spin systems can be made to interact via a π-conjugated backbone without detriment to their long spin coherence times. This is exactly what is required for quantum information processing with molecular electron-spin systems: a scaffold onto which quantum-coherent units can be assembled and through which interactions between spins are transmitted. The stronger the interaction, the longer the gating time usually necessary to perform operations, e.g., via the Hadamard transform.^44^ Edge-fused porphyrin dimers confer an appropriate level of interaction. The Gd^III^ complexes studied here have MHz-range interactions and phase-memory times up to 10 μs at low temperatures, which would allow several hundred operations within the coherence time of the Gd^III^ centers. Such values are long enough to test quantum computing schemes using microwave pulses and open up the path for information processing in single-molecule electronic devices.^45^ This is exciting because the porphyrin scaffolds allow extended multicenter systems to be constructed via controlled oligomerization,^12−14^ which provides an additional dimension compared with previous coordination dimers.^10^ This work illustrates the possibility of tuning the interaction synthetically, using a variety of fully delocalized or partially delocalized backbones.

Tunneling in ***f*****-P2-*****E*****·Dy**_**2**_ and ***f*****-P2-*****Z*****·Dy**_**2**_ is visible at mK temperatures and shows strong dependence on the *syn* vs *anti* stereochemistry. These results confirm the previous observation of strong environmental effects produced by delocalized π states, for example, in graphene,^46^ affording insights into SMMs on surfaces and carbon nanotubes, with geometrical discriminations that would otherwise not be available for spins grafted onto π-conjugated materials. This is a key step toward the creation of molecularly tailored magnetic materials that are based on an aromatic plane and metal centers. Metalloporphyrin oligomers^12−14^ are an appealing family of compounds for the creation of aromatic materials with magnetic properties because almost every metal in the periodic table can be inserted into a porphyrin. The current strategy can be extended to long multiple porphyrin chains, with up to 24 units for edge-fused tapes using published synthetic methods.^13^ There is also scope for extending this approach to nanorings that bear molecular magnets and support fully delocalized electronic states.^47^ The observed interactions thus indicate encouraging perspectives for the use of these materials for multicenter quantum units. The integration of more complex metalloporphyrin oligomers^12−14,47^ into polyfunctional electronic devices, where the spin properties can be followed using single-molecule transport tools, is now within reach.

## Supporting Information Available

Details of synthetic protocols, mass spectra and GPC traces, electrochemistry, magnetic characterization, EPR spectroscopy, and torque magnetometry (PDF)

## Accession Codes

CCDC 2154038–2154041 contain the supplementary crystallographic data for this paper. These data can be obtained free of charge via www.ccdc.cam.ac.uk/data_request/cif, or by emailing data_request@ccdc.cam.ac.uk, or by contacting The Cambridge Crystallographic Data Centre, 12 Union Road, Cambridge CB2 1EZ, UK; fax: +44 1223 336033.
