# Supplementary material for: Singly and Triply Linked Magnetic Porphyrin Lanthanide Arrays
Source: J Am Chem Soc. 2022 May 3;144(19):8693–706. doi: 10.1021/jacs.2c02084 (PMC9121389; doi:10.1021/jacs.2c02084)
Supplement: Supplementary file 1 — ja2c02084_si_001.pdf [file ja2c02084_si_001.pdf]

Supporting Information for

Singly- and Triply-Linked Magnetic Porphyrin Lanthanide Arrays

Jeff M. Van Raden,[a]† Dimitris I. Alexandropoulos,[b]† Michael Slota,[b]† Simen Sopp,[b] Taisuke Matsuno,[c] Amber L. Thompson,[a] Hiroyuki Isobe,[c] Harry L. Anderson,[a]* and Lapo Bogani[b]*

[a] Department of Chemistry, University of Oxford, Chemistry Research Laboratory, Oxford, OX1 3TA, UK

[b] Department of Materials, University of Oxford, OX1 3PH, Oxford, UK

[c] Department of Chemistry, The University of Tokyo, Tokyo 113-0033, Japan

† These authors contributed equally.

Table of Contents

1. General methods S2

2. Synthetic details for known compounds S2

3. Procedures for synthesis of Ln^{III} complexes S2

4. MALDI-TOF spectra and analytical GPC traces for all complexes S7

5. Summary of electrochemical data S9

6. Chiral HPLC analysis, optical resolution and circular dichroism spectroscopy of ***s*-P2·Dy₂** S11

7. Magnetic characterization: SQUID magnetometry S13

8. Electron spin resonance spectroscopy (EPR) S32

9. Torque magnetometry S39

10. References S42

1. General methods

All reagents were purchased from commercial sources and solvents were used as supplied unless otherwise noted. All solvents (except those used for workups and chromatography), were obtained by passing the solvents through columns of alumina, under nitrogen. Flash chromatography was carried out on silica gel 60 under positive pressure. Analytical thin-layer chromatography was carried out on aluminum-backed silica gel 60 F254 plates. Visualization was achieved using UV light when necessary. MALDI-ToF spectra were measured at the University of Oxford using Waters MALDI Micro MX spectrometer. UV-vis-NIR spectra were recorded in solution using a Perkin-Lambda 20 spectrometer (1 cm path length quartz cell). All measurements were carried out at 25 °C under ambient conditions.

2. Synthetic details for known compounds

Compounds **P1·H$_2$**,[1] ***s*-P2·Zn$_2$**,[2] ***f*-P2·Zn$_4$**,[2] and **NaL**[3] were prepared according to literature procedures.

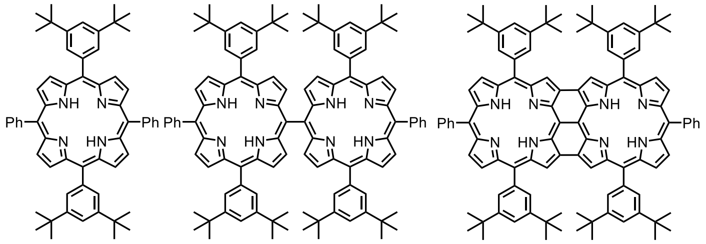

P1·H$_2$ ***s*-P2·H$_4$**   ***f*-P2·H$_4$**

Demetalation of *s*-P2·Zn$_2$. To a 250 mL round bottom flask equipped with a stir bar was added ***s*-P2·Zn$_2$** (0.132 g, 0.0806 mmol, 1.00 equiv.) followed by CH_2Cl_2 (125 mL). To this flask was then added trifluoroacetic acid (0.100 mL, 1.30 mmol, 16.1 equiv.) resulting in a color change from dark orange to brown. After stirring for 1 h, triethylamine was added (1.00 mL) and the solution was passed through a short silica plug (eluted with CH_2Cl_2). The brownish-red eluent was then condensed resulting in a dark brown solid. This solid was then washed with methanol (5.00 mL) and dried to give ***s*-P2·H$_4$** (0.122 g, 93%) as a dark red/brown solid. Characterization data were consistent with the literature.[4,5]

Demetalation of *f*-P2·Zn$_2$. To a 250 mL round bottom flask equipped with a stir bar was added ***f*-P2·Zn$_2$** (0.113 g, 0.0687 mmol, 1.00 equiv.) followed by CH_2Cl_2 (125 mL). To this flask was then added trifluoroacetic acid (0.100 mL, 1.30 mmol, 18.9 equiv.) resulting in a color change from dark purple to brown. After stirring for 1 h, triethylamine was added (1.00 mL) and the solution was passed through a short silica plug (eluted with CH_2Cl_2). The brownish-red eluent was then condensed resulting in a dark brown/black solid. This solid was then washed with methanol (5.00 mL) and dried to give ***f*-P2·H$_4$** (96.1 mg, 85%) as a brown/black solid. Characterization data were consistent with the literature.[6]

3. Procedures for synthesis of Ln^{III} complexes

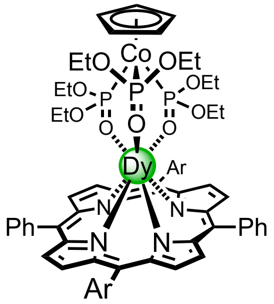

Synthesis of P1·Dy. To a flame-dried Schlenk flask equipped with a stir bar was added free-base porphyrin monomer **P1·H$_2$** (15.0 mg, 0.0179 mmol, 1.00 equiv.), $DyCl_3·6H_2O$ (67.3 mg, 0.179 mmol, 10.0 equiv.) followed by imidazole (1.0 g), diphenyl ether (1.0 g), and sulfolane (1.0 mL). The contents of the flask were evacuated and back-filled with argon 5 times. While under argon, the contents were then heated at 250 °C in a sand bath until complete consumption of **P1·H$_2$** was observed (monitored via TLC; typically 30–45 min). At this point, the flask was removed from the sand bath and allowed to cool to room temperature, then diluted with H_2O (10 mL) and extracted with dichloromethane (3 × 10 mL). The organic phase was then washed with H_2O (2 × 10 mL), followed by brine (1 × 10 mL), and then finally dried over sodium sulfate. The solvent was then removed to give a dark red/purple oil. A stir bar was added to the flask followed by dissolution of the oil in chloroform (10 mL). At this point, **NaL** (15.4 mg, 0.0268 mmol, 1.5 equiv.) was added as a solid. The contents were stirred for 30 min. The solvent was removed to give a dark red/purple oil which was chromatographed on SiO_2 (0 to 100%

petroleum ether/dichloromethane). The dark purple band was collected to give crude **P1·Dy**. Trituration of this residue with methanol yielding pure **P1·Dy** as a dark red/purple solid (22.9 mg, 83%).

MALDI-TOF P1·Dy: *m/z* = 1534.547 ($C_{77}H_{95}CoDyN_4O_9P_3$, calculated 1534.49)

λ_{max} (CHCl$_3$)/ nm log (ε) P1·Dy: 430 (5.66), 560 (4.21), 601 (3.72)

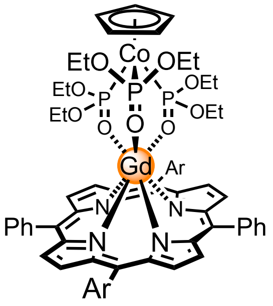

Synthesis of P1·Gd. To a flame-dried Schlenk flask equipped with a stir bar was added free-base porphyrin monomer **P1·H₂** (32.1 mg, 0.0383 mmol, 1.00 equiv.), $GdCl_3$ (50.3 mg, 0.191 mmol, 5.00 equiv.) followed by imidazole (1.0 g), diphenyl ether (1.0 g). The contents of the flask were evacuated and back-filled with argon 5 times. While under argon, the contents were then heated at 250 °C in a sand bath until complete consumption of **P1·H$_2$** was observed (monitored via TLC; typically 30–45 min). At this point, the flask was removed from the sand bath and allowed to cool to room temperature, then diluted with H_2O (10 mL) and extracted with dichloromethane (3 × 10 mL). The organic phase was washed with H_2O (2 × 10 mL), followed by brine (1 × 10 mL), and then dried over sodium sulfate. The solvent was removed to give a dark red/purple oil. A stir bar was added to the flask followed by dissolution of the oil in chloroform (10 mL). At this point, **NaL** (31.6 mg, 0.0575 mmol, 1.5 equiv.) was added as a solid. The contents were stirred for 30 min. The solvent was removed to give a dark red/purple oil which was then chromatographed on SiO_2 (0 to 100% petroleum ether/dichloromethane). The dark purple band was collected to give crude **P1·Gd**. Trituration of this residue with methanol yielding pure **P1·Gd** as a dark red/purple solid (51.1 mg, 86%).

MALDI-TOF P1·Dy: *m/z* = 1529.916 ($C_{77}H_{95}CoGdN_4O_9P_3$, calculated 1529.49)

λ_{max}(CHCl$_3$)/ nm log (ε) P1·Gd: 430 (5.66), 560 (4.21), 601 (3.72)

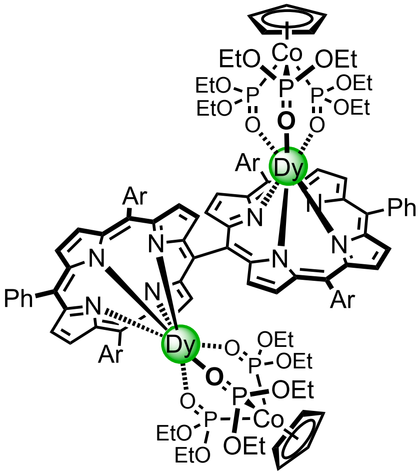

Synthesis of *s*-P2·Dy$_2$. To a flame-dried Schlenk flask equipped with a stir bar was added **sP2·H$_4$** (100 mg, 65.6 μmol, 1.00 equiv.), $DyCl_3{\cdot}6H_2O$ (176 mg, 0.656 mmol, 10.0 equiv.) followed by imidazole (1.0 g), diphenyl ether (1.0 g). The contents of the flask were evacuated and back-filled with argon 5 times. While under argon, the contents were then heated at 250 °C in a sand bath until complete consumption of porphyrin dimer was observed (monitored via TLC; typically 30–45 min). At this point, the flask was removed from the sand bath and allowed to cool to room temperature, then diluted with H_2O (10 mL) and extracted with dichloromethane (3 × 10 mL). The organic phase was then washed with H_2O (2 × 10 mL), followed by brine (1 × 10 mL), and then finally dried over sodium sulfate. The solvent was then removed to give a dark brown/yellow oil. A stir bar was added to the flask followed by dissolution of the oil in chloroform (10 mL). At this point, Klaüi ligand (79.4 mg, 0.144 mmol, 2.2 equiv.) was added as a solid. The contents were stirred for 30 min. The solvent was removed to give a dark brown/yellow oil which was chromatographed on SiO_2 (0 to 100% petroleum ether/dichloromethane). The dark brown band was collected to give crude ***s*-P2·Dy$_2$**. Trituration of this residue with methanol yielded pure ***s*-P2·Dy$_2$** as a dark brown solid (149.3 mg, 78%).

MALDI-TOF *s*-P2·Dy$_2$: *m/z* = 2916.207 ($C_{142}H_{180}Co_2Dy_2N_8O_{18}P_6$, calculated 2916.9089)

λ_{max}(CHCl$_3$)/ nm log (ε) *s*-P2·Dy$_2$: 431 (5.28), 467 (5.24), 576 (4.63)

Synthesis of *s*-P2·Gd$_2$. To a flame-dried Schlenk flask equipped with a stir bar was added ***s*-P2·H$_4$** (105 mg, 69.1 μmol, 1.00 equiv.), $GdCl_3$ (182 mg, 0.691 mmol, 10.0 equiv.) followed by imidazole (1.0 g), diphenyl ether (1.0 g). The contents of the flask were evacuated and back-filled with argon 5 times. While under argon, the contents were then heated at 250 °C in a sand bath until complete consumption of porphyrin dimer was observed (monitored via TLC; typically 30–45 min). At this point, the flask was removed from the sand bath and allowed to cool to room temperature, then diluted with H_2O (10 mL) and extracted with dichloromethane (3 × 10 mL). The organic phase was then washed with H_2O (2 × 10 mL), followed by brine (1 × 10 mL), and then finally dried over sodium sulfate. The solvent was then removed to give a dark brown/yellow oil. A stir bar was added to the flask followed by dissolution of the oil in chloroform (10 mL). At this point, Klaüi ligand (83.8 mg, 0.152 mmol, 2.2 equiv.) was added as a solid. The contents were stirred for 30 min. The solvent was removed to give a dark brown/yellow oil which was chromatographed on SiO_2 (0 to 100% petroleum ether/dichloromethane). The dark brown band was collected to give crude ***s*-P2·Gd$_2$**. Trituration of this residue with methanol yielded pure ***s*-P2·Gd$_2$** as a dark brown solid (162.5 mg, 78%).

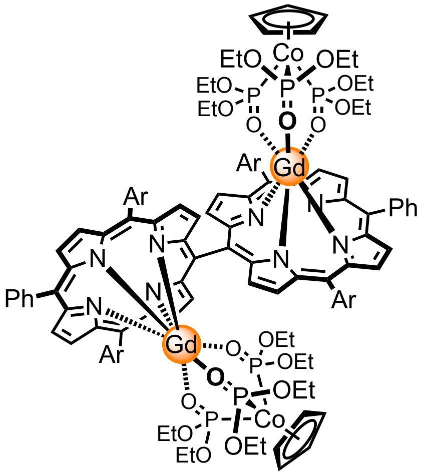

MALDI-TOF *s*-P2·Gd$_2$: m/z = 2904.141 ($C_{142}H_{180}Co_2Gd_2N_8O_{18}P_6$, calculated 2904.8987)

λ_{max}(CHCl$_3$)/ nm log (ε) *s*-P2·Gd$_2$: 431 (5.28), 467 (5.24), 576 (4.63)

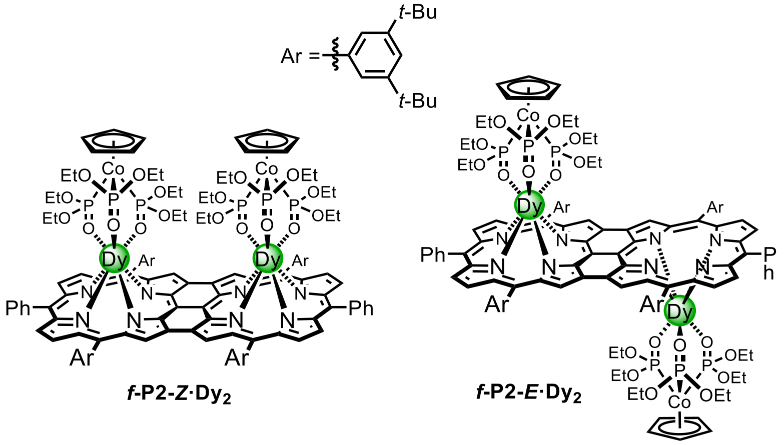

Synthesis of *f*-P2-*E*·Dy$_2$ and *f*-P2-*Z*·Dy$_2$. To a flame-dried Schlenk flask equipped with a stir bar was added free-base porphyrin dimer ***f*-P2·H$_4$** (71.2 mg, 0.0468 mmol), DyCl$_3$·6H$_2$O (0.176 g, 0.468 mmol) followed by imidazole (1.0 g) and diphenyl ether (1.0 g). The contents of the flask were evacuated and back-filled with argon 5 times. While under argon, the contents were heated at 250 °C in a sand bath until complete consumption of ***f*-P2·H$_4$** was observed (monitored via TLC; typically, 30–45 min). The flask was then removed from the sand bath and allowed to cool to room temperature at which point the reaction was diluted with H_2O (10 mL) and extracted with dichloromethane (3 × 10 mL). The organic phase was then washed with H_2O (2 × 10 mL), followed by brine (10 mL), and then finally dried over sodium sulfate. The solvent was removed to give a dark greenish/blue oil. In the same flask, a stir bar was added followed by dissolution of the oil in chloroform (10 mL). To this flask, **NaL** (64.4 mg, 0.117 mmol, 2.5 equiv.) was added as a solid and stirred for 30 min. The solvent was removed to give a dark green/blue oil which was then chromatographed on SiO_2 (0 to 100% petroleum ether/dichloromethane) to remove the majority of impurities. The dark green/blue band was collected to give a crude mixture of ***f*-P2-*E*·Dy$_2$** and ***f*-P2-*Z*·Dy$_2$** as a dark green/blue residue. Trituration of this residue with methanol yielding a mixture of ***f*-P2-*Z*·Dy$_2$** and ***f*-P2-*E*·Dy$_2$** as a dark green/blue solid. This mixture was then purified via chromatography (15% diethyl ether/petroleum ether), followed by recrystallization from $CHCl_3$/ethyl acetate to give ***f*-P2-*Z*·Dy$_2$** (49.3 mg, 51%) and ***f*-P2-*E*·Dy$_2$** (29.0 mg, 30%) as dark green/purple solids.

Selective synthesis of *f*-P2-*E*·Dy$_2$. To a 25-mL round bottom flask equipped with a stir bar was added ***s*-P2·Dy$_2$** (4.90 mg, 0.00167 mmol) followed by CH_2Cl_2 (5.00 mL). To this solution was added [bis(trifluoracetoxy)iodo]benzene (3.61 mg, 0.00840 mmol) as a solution in CH_2Cl_2 (2.00 mL). The reaction was then allowed to proceed for 3 h, turning from an orange/brown color to dark blue. The reaction was then quenched by adding $NaBH_4$ (2.90 mg, 0.0841 mmol) and methanol (1.00 mL). The contents were then passed through a short plug of SiO_2 ($CHCl_3$) and the dark green fraction was collected and the solvent was removed. The resulting solid was then washed with methanol and dried to give ***f*-P2-*E*·Dy$_2$** (4.18 mg, 86%). The analytical GPC trace and observed MALDI-TOF data were identical to that obtained above for ***f*-P2-*E*·Dy$_2$** and no traces of ***f*-P2-*Z*·Dy$_2$** were observed.

MALDI-TOF *f*-P2-*E*·Dy$_2$: *m/z* = 2911.866 ($C_{142}H_{176}Co_2Dy_2N_8O_{18}P_6$, calculated 2912.88)

MALDI-TOF *f*-P2-*Z*·Dy$_2$: *m/z* = 2911.904 ($C_{142}H_{176}Co_2Dy_2N_8O_{18}P_6$, calculated 2912.88)

λ_{max}(CHCl$_3$)/ nm log (*ε*) *f*-P2-*E*·Dy$_2$: 427 (5.15), 592 (5.06), 975 (4.26), 1119 (4.53)

λ_{max}(CHCl$_3$)/ nm log (*ε*) *f*-P2-*Z*·Dy$_2$: 426 (5.16), 571 (5.05), 956 (4.26), 1093 (4.51)

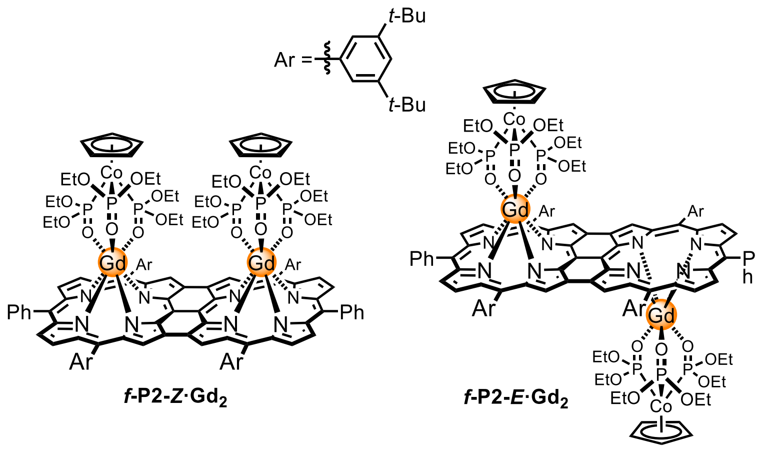

Synthesis of *f*-P2-*E*·Gd_2 and *f*-P2-*Z*·Gd_2. To a flame-dried Schlenk flask equipped with a stir bar was added free-base porphyrin dimer **P2·H_4** (52.1 mg, 0.0343 mmol), $GdCl_3$ (90.1 mg, 0.343 mmol) followed by imidazole (1.0 g), diphenyl ether (1.0 g). The contents of the flask were evacuated and back-filled with argon 5 times. While under argon, the contents were then heated at 250 °C in a sand bath until complete consumption of **P2·H_4** was observed (monitored via TLC; typically, 30–45 min). At this point, the flask was removed from the sand bath and allowed to cool to room temperature, then the reaction contents were filtered to remove insoluble impurities. Both the flask and filtered material were then washed with CH_2Cl_2 providing a teal/green filtrate. The filtrate was then collected, diluted with H_2O (10 mL) and finally extracted with dichloromethane (3 × 10 mL). The organic phase was then washed with H_2O (10 mL), followed by brine (10 mL), and then finally dried over sodium sulfate. The solvent was then removed to give a dark greenish/blue oil. A stir bar was added to the flask followed by dissolution of the oil in chloroform (10 mL). At this point, **NaL** (47.2 mg, 0.0858 mmol, 2.5 equiv.) was added as a solid and stirred for 30 min. The solvent was then removed to give a dark green/blue oil which was chromatographed SiO_2 (0 to 100% petroleum ether/dichloromethane) to remove the majority of impurities. The dark green/blue band was collected to give a crude mixture of ***f*-P2-*E*·Gd_2** and ***f*-P2-*Z*·Gd_2** as a dark green/blue residue. Trituration of this residue with methanol yielding a mixture of ***f*-P2-*Z*·Gd_2** and ***f*-P2-*E*·Gd_2** as a dark green/blue solid. This mixture was then purified via chromatography (15% diethyl ether/petroleum ether), followed by recrystallization from $CHCl_3$/ethyl acetate to give ***f*-P2-*Z*·Gd_2** (28.1 mg, 28%) and ***f*-P2-*E*·Gd_2** as (12.9 mg, 13%) as dark green/purple solids.

Selective synthesis of *f*-P2-*E*·Gd_2. To a 25-mL round bottom flask equipped with a stir bar was added ***s*-P2·Gd_2** (4.71 mg, 0.00162 mmol) followed by CH_2Cl_2 (5.00 mL). To this solution was added [bis(trifluoracetoxy)iodo]benzene (3.47 mg, 0.00811 mmol) as a solution in CH_2Cl_2 (2.00 mL). The reaction was then allowed to proceed for 3 h, turning from an orange/brown color to dark blue. The reaction was then quenched by adding $NaBH_4$ (3.01 mg, 0.0811 mmol) and methanol (1.00 mL). The contents were then passed through a short plug of SiO_2 ($CHCl_3$) and the dark green fraction was collected and the solvent was removed. The resulting solid was then washed with methanol and dried to give ***f*-P2-*E*·Gd_2** (3.11 mg, 66%). The analytical GPC trace and observed MALDI-TOF data were identical to that obtained above for ***f*-P2-*E*·Gd_2** and no traces of ***f*-P2-*Z*·Gd_2** were observed.

MALDI-TOF *f*-P2-*E*·Gd_2: m/z = 2900.881 ($C_{142}H_{176}Co_2Gd_2N_8O_{18}P_6$, calculated 2900.87)

MALDI-TOF *f*-P2-*Z*·Gd_2: m/z = 2900.711 ($C_{142}H_{176}Co_2Gd_2N_8O_{18}P_6$, calculated 2900.87)

λ_{max}($CHCl_3$)/ nm log (ε) *f*-P2-*E*·Gd_2: 427 (5.16), 593 (5.06), 977 (4.26), 1123 (4.55)

λ_{max}($CHCl_3$)/ nm log (ε) *f*-P2-*Z*·Gd_2: 426 (5.16), 575 (5.07), 958 (4.27), 1096 (4.54)

4. MALDI-MS spectra and analytical GPC traces for all complexes

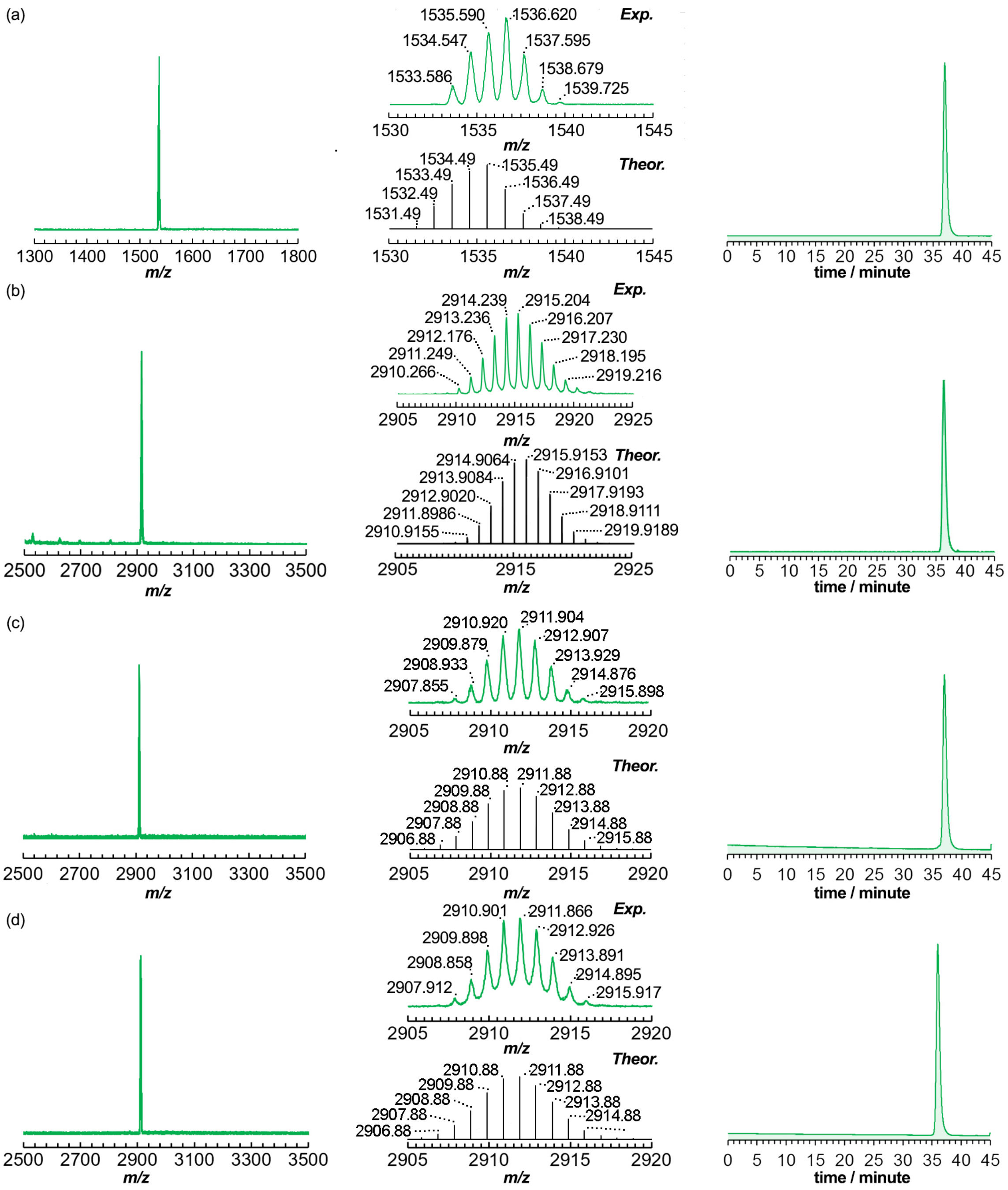

Figure S1. Experimental and theoretical MALDI-MS (full spectra, left and zoomed spectra, center) and analytical GPC traces (right) for complexes: (a) **P1·Dy**; (b) ***s*-P2·Dy$_2$**; (c) ***l*-P2-*Z*-2·Dy$_2$**; (d) ***l*-P2-*E*·Dy$_2$**. PLGel columns, THF + 1% pyridine, detection at 400 nm.

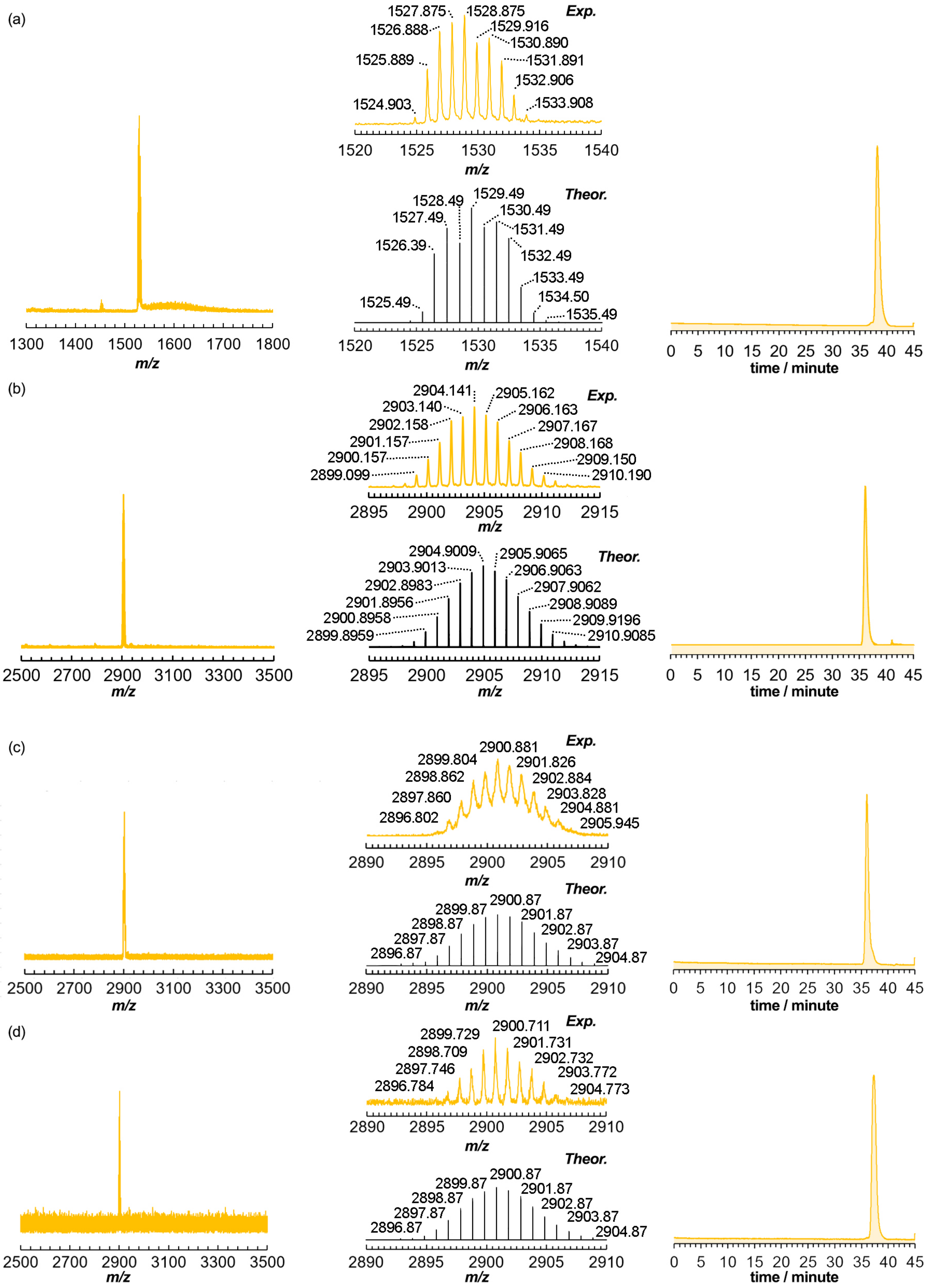

Figure S2. Experimental and theoretical MALDI-MS (full spectra, left and zoomed spectra, center) and analytical GPC traces (right) for complexes: (a) **P1·Gd**; (b) ***s*-P2·Gd$_2$**; (c) ***f*-P2-*E*·Gd$_2$**; (d) ***f*-P2-*Z*·Gd$_2$**. PLGel columns, THF + 1% pyridine, detection at 400 nm.

5. Summary of electrochemical data

Electrochemical experiments were carried out using an Autolab PGSTAT 12 with a 3 mm glass-carbon working electrode, platinum wire counter electrode and Ag/AgNO$_3$ (0.01 M in acetonitrile) reference electrode and tetra-*n*-butylammonium hexafluorophosphate as supporting electrolyte (0.1 M in CH$_2$Cl$_2$). Voltammograms were referenced to the Fc/Fc$^+$ couple (0.0 V) as an internal reference.

Table S1. Summary of redox potentials (all relative to internal Fc/Fc$^+$ couple at 0.0 V).

complex	E_{ox1}	E_{ox2}	E_{ox3}	E_{ox4}	E_{ox5}
***s*-P2-Zn$_2$**	0.36 V	0.47 V	0.71 V	0.80 V	1.09 V
***s*-P2-Dy$_2$**	0.12 V	0.44 V	0.86 V	0.96 V	1.26 V
***s*-P2-Gd$_2$**	0.11 V	0.42 V	0.87 V	0.97 V	1.24 V
***f*-P2-*E*-Dy$_2$**	–0.27 V	0.20 V	0.87 V	1.22 V	--
***f*-P2-*Z*-Dy$_2$**	–0.19 V	0.22 V	0.87 V	1.23 V	--
***f*-P2-*E*-Gd$_2$**	–0.29 V	0.22 V	0.90 V	1.22 V	--
***f*-P2-*Z*-Gd$_2$**	–0.20 V	0.20 V	0.87 V	1.23 V	--
***f*-P2-Zn$_2$**	0.04 V	0.27 V	0.76 V	1.15 V	--

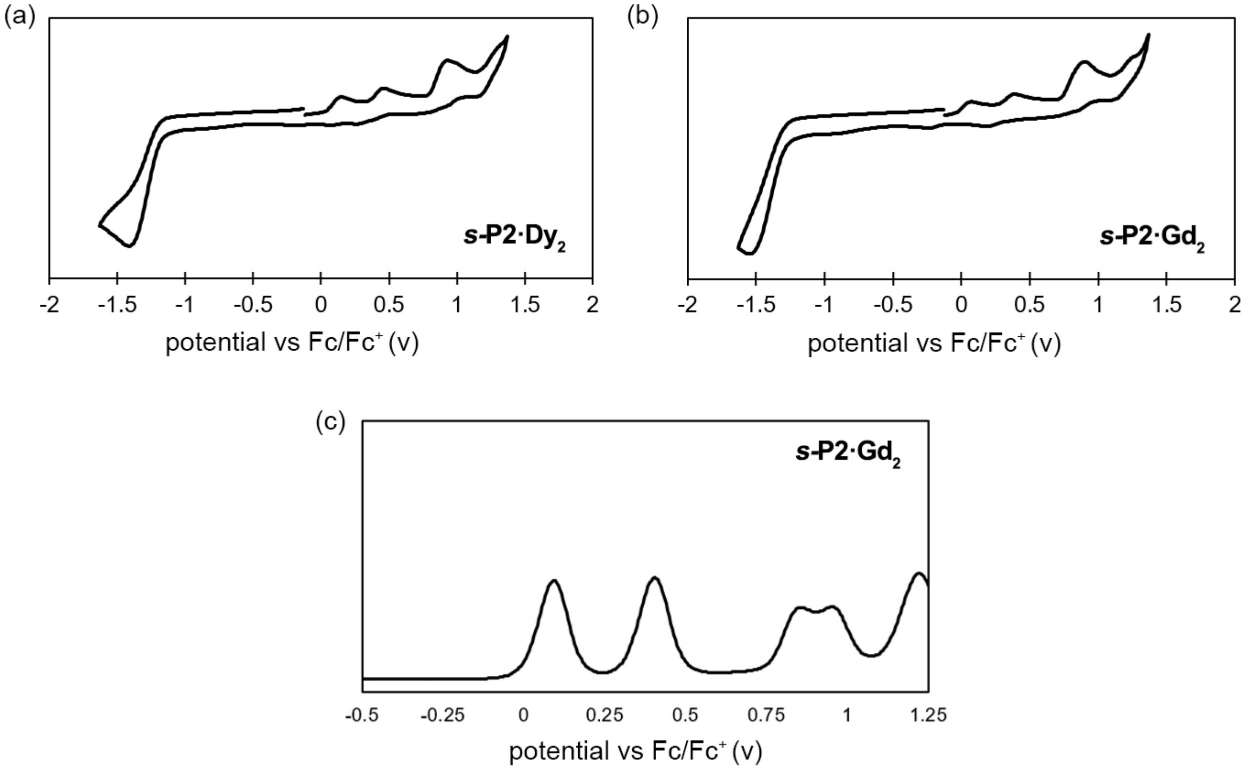

Figure S3. Cyclic voltammograms of (a) ***s*-P2·Dy$_2$** and (b) ***s*-P2·Gd$_2$** and differential pulse voltammograms of (c) ***s*-P2·Gd$_2$**.

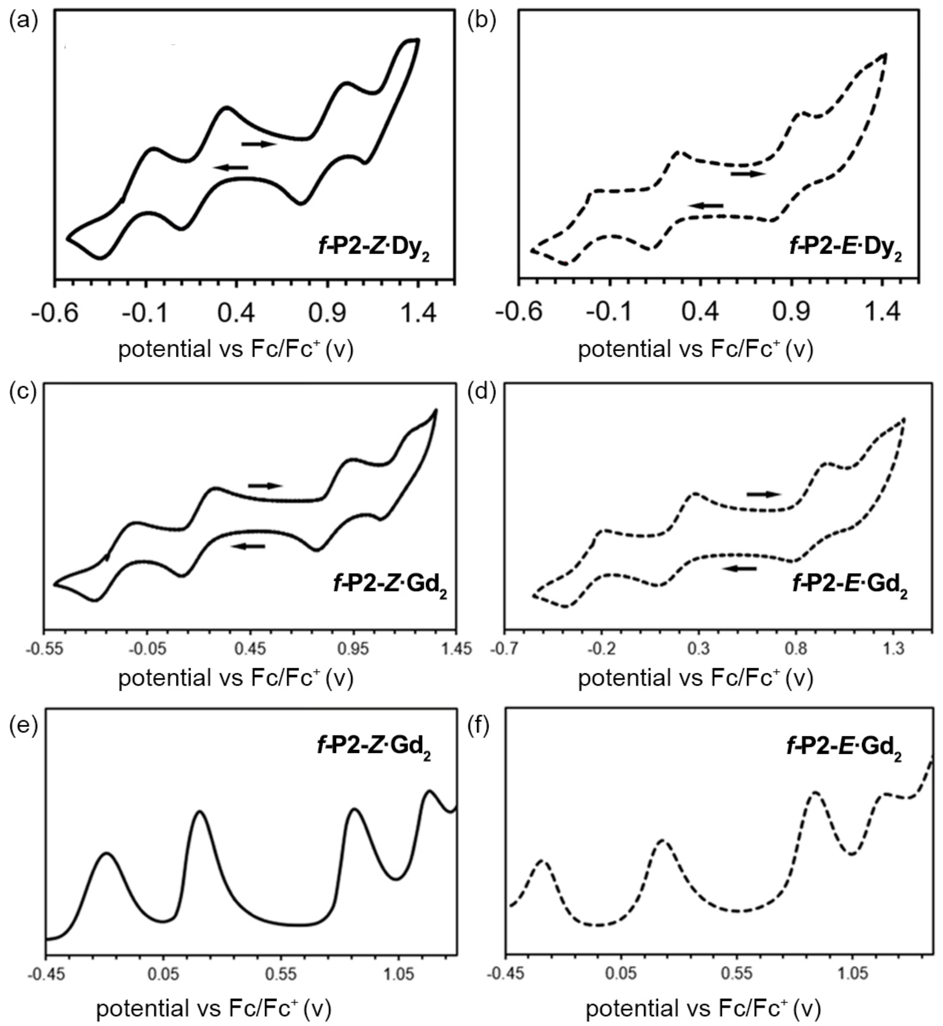

Figure S4. Cyclic voltammograms of (a) ***f*-P2-*Z*·Dy$_2$** and (b) ***f*-P2-*E*·Dy$_2$** (c) ***f*-P2-*Z*·Gd$_2$** and (d) ***f*-P2-*E*·Gd$_2$** and differential pulse voltammograms of (e) ***f*-P2-*Z*·Gd$_2$** and (f) ***f*-P2-*E*·Gd$_2$**.

6. Chiral HPLC Analysis, Optical Resolution and Circular Dichroism Spectroscopy of *s*-P2·Dy_2

(a) Chiral Resolution. All chiral HPLC analyses and the subsequent chiral resolution of ***s*-P2·Dy_2** were carried out using a HPLC system equipped with a SUMICHIRAL OA-2500 4.6φ × 250 mm column at 40 °C in a column oven (JASCO CO-2060PLUS) under the detection with UV-vis (JASCO UV-2075PLUS) and CD (JASCO CD-2095) detectors at flow rate of 1.0 mL min^{-1} (JASCO PU-2089PLUS). To obtain sufficient amounts of resolved ***s*-P2·Dy_2** for analysis, approximately 1 mg of material was first dissolved in hexane. Enantiomerically pure **$(+)_{400}$-*s*-P2·Dy_2** and **$(-)_{400}$-*s*-P2·Dy_2** were then obtained over 8 separate injections of 90 μL aliquot.

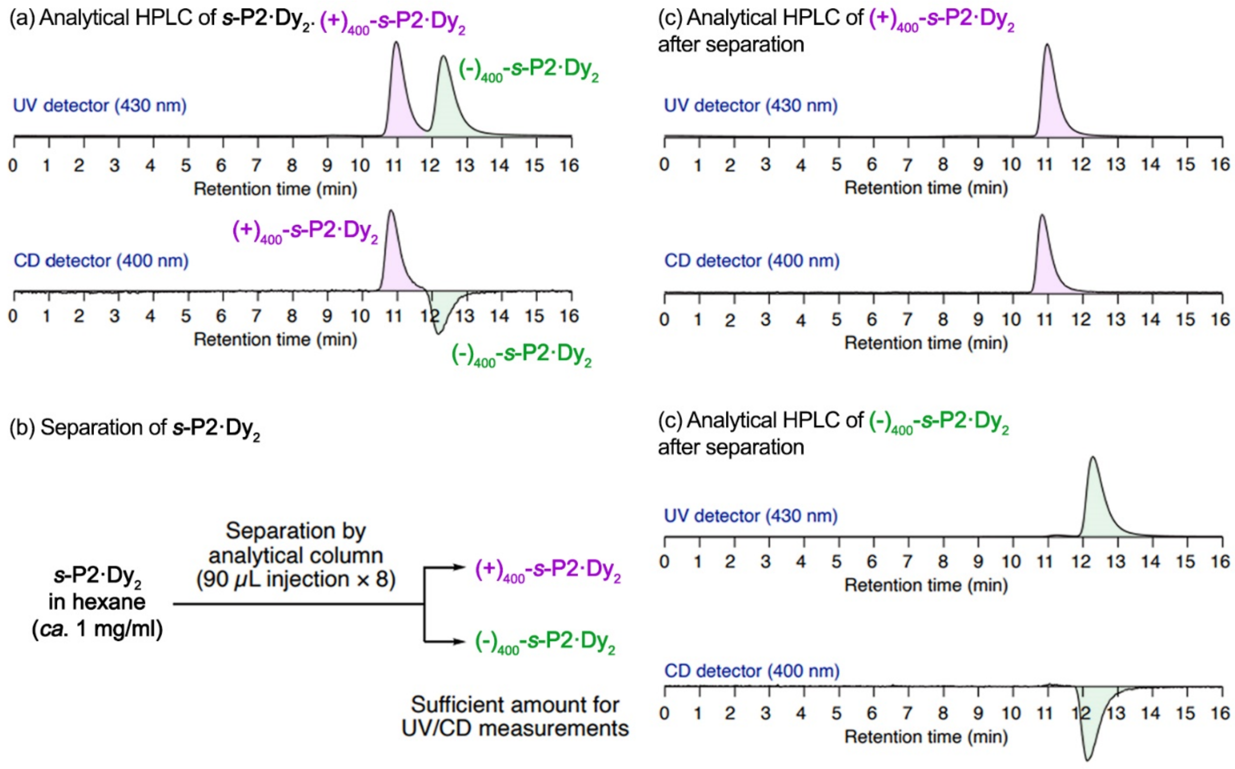

Figure S5. Chiral HPLC analyses and separation of ***s*-P2·Dy_2**. (a) chromatograms of ***s*-P2·Dy_2**. (b) Overview of separation of ***s*-P2·Dy_2** to **$(+)_{400}$-*s*-P2·Dy_2** and **$(-)_{400}$-*s*-P2·Dy_2**. (c) Chromatograms of **$(+)_{400}$-*s*-P2·Dy_2** after separation. (d) Chromatograms of **$(-)_{400}$-*s*-P2·Dy_2** after separation. Column: SUMICHIRAL OA-2500 4.6φ × 250 mm; column oven: 40 °C; UV detector: 430 nm; CD detector: 400 nm; eluent: hexane/*i*PrOH (99:1 *v*/*v*); flow rate: 1.0 mL min^{-1}.

(b) Attempted Racemization of *s*-P2·Dy$_2$

To dry a 10 mL Schlenk tube equipped with a high-vacuum greaseless valve was added a diluted solution of **(+)$_{400}$-*s*-P2·Dy$_2$** in toluene (1 mL). The solution was degassed three times by freeze-pump-thaw cycles and then sealed. The contents were protected from light, then heated at 200 °C for 24 h. The reaction outcome was analyzed via HPLC and the results are shown in Figure S6. As can be seen, after 24 h, **(–)$_{400}$-*s*-P2·Dy$_2$** was not detected (Figure S6b) nor did we find any signs of decomposition. This indicates that **(+)$_{400}$-*s*-P2·Dy$_2$** has a high barrier to racemization.

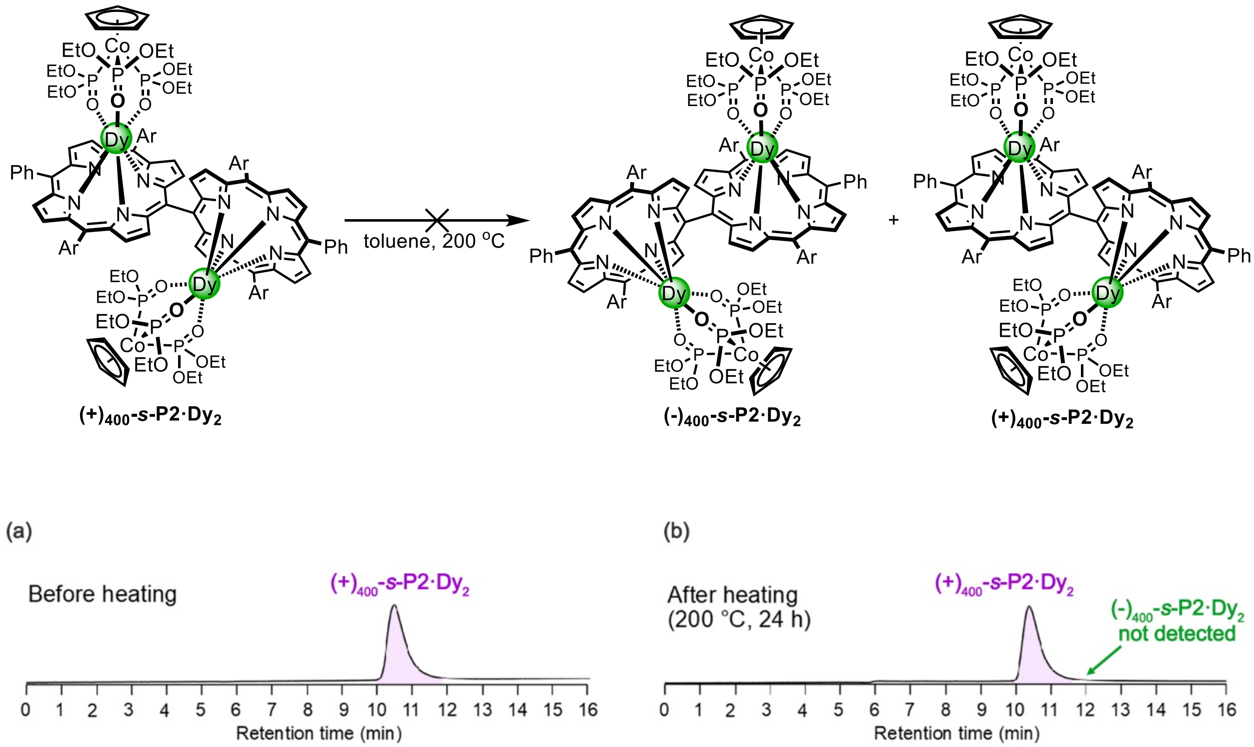

Figure S6. Attempted racemization of **(+)$_{400}$-*s*-P2·Dy$_2$**. Chromatograms before (a) and after (b) heating **(+)$_{400}$-*s*-P2·Dy$_2$** in toluene for 200 °C for 24 h. Column: SUMICHIRAL OA-2500 4.6φ × 250 mm; column oven: 40 °C; UV detector: 430 nm; CD detector: 400 nm; eluent: hexane/*i*PrOH (99:1 v/v); flow rate: 1.0 mL min^{-1}.

(c) Circular Dichroism Spectroscopy of *s*-P2·Dy$_2$

CD spectra were measured by using a spectropolarimeter (JASCO J-1500) at 25 °C equipped with a Peltier cell holder (JASCO MPTC-511) for temperature controls. The concentration of sample solutions was determined by comparing the absorbance at 430 nm (JASCO V-670) with $\varepsilon = 1.9 \times 10^5$ M^{-1} cm^{-1}. A quartz cell with an optical path length of 1 cm was used for measurements.

7. Magnetic characterization: SQUID magnetometry

Variable-temperature direct current (dc) magnetic susceptibility data were collected on a Quantum Design MPMS-XL SQUID magnetometer equipped with a 7 T magnet and operating in the 2–300 K range. Pascal's constants were used to estimate the diamagnetic corrections, which were subtracted from the experimental susceptibilities to give the molar paramagnetic susceptibilities (χM). Magnetization data were collected at 2 K, 5 K and 7 K at magnetic fields ranging from 1–7 T. Alternating current (ac) susceptibility data were collected in a 0.2 mT field oscillating at 1–1000 Hz frequencies.

Fitting of the dc susceptibility and magnetization data

Magnetic susceptibility data of the **1·Gd**, ***Z*-2·Gd₂** and ***E*-2·Gd₂**, and their Dy counterparts, were fitted using EasySpin.[7] The Spin Hamiltonian is defined as follows:

$$\mathrm{H} = \mathrm{H}_Z + H_{\mathrm{ZFS}} + H_{\mathrm{ee}},$$

Where H_Z accounts for the Zeeman effect, H_{ZFS} the zero-field splitting and H_{ee} the electron-electron interaction Hamiltonian. Here, only second order zero-field splitting parameters were considered, i. e. the axial and transverse anisotropy terms D and E, as higher order terms lead to over-parameterization. In the case of Gd, these values were directly obtained from the EPR spectra (see below) and kept fixed in the fitting of the susceptibility data. For electron-electron interactions in the dimers, we only considered isotropic exchange coupling. Due to over-parameterization, electron-electron interactions were omitted in the case of Dy. The fit results are shown in Fig. 8 in the main text, whereas Fig. S7–S14 show calculations of the M versus B behavior based on the fit parameters. Table S2 shows the best fit parameters.

Table S2. Obtained fit parameters from the magnetic susceptibility data. [Note that in the case of Gd, D and E were taken from EPR data and kept fixed (see Table S7). No J was considered in the case of Dy to avoid over-parameterization.]

	g	J (MHz)	D (GHz)	E (GHz)
**1·Gd**	1.9942(55)	n/a	[−3.5 ± 0.1]	[0.30 ± 0.05]
***s*-P2·Gd₂**	1.9980(201)	0	[−3.5 ± 0.1]	[0.30 ± 0.05]
***f*-P2-*Z*·Gd₂**	1.9919(127)	−51 ± 2	[−3.6 ± 0.1]	[0.25 ± 0.05]
***f*-P2-*E*·Gd₂**	2.0082(382)	−19 ± 3	[−3.6 ± 0.1]	[0.25 ± 0.05]
**1·Dy**	1.3449(63)	n/a	−510.0 ± 0.4	603.9 ± 0.5
***f*-P2-*Z*·Dy₂**	1.3457(233)	n/a	−531.2 ± 156.2	449.5 ± 30.7
***f*-P2-*E*·Dy₂**	1.3445(83)	n/a	−870.1 ± 145.4	779.7 ± 36.7

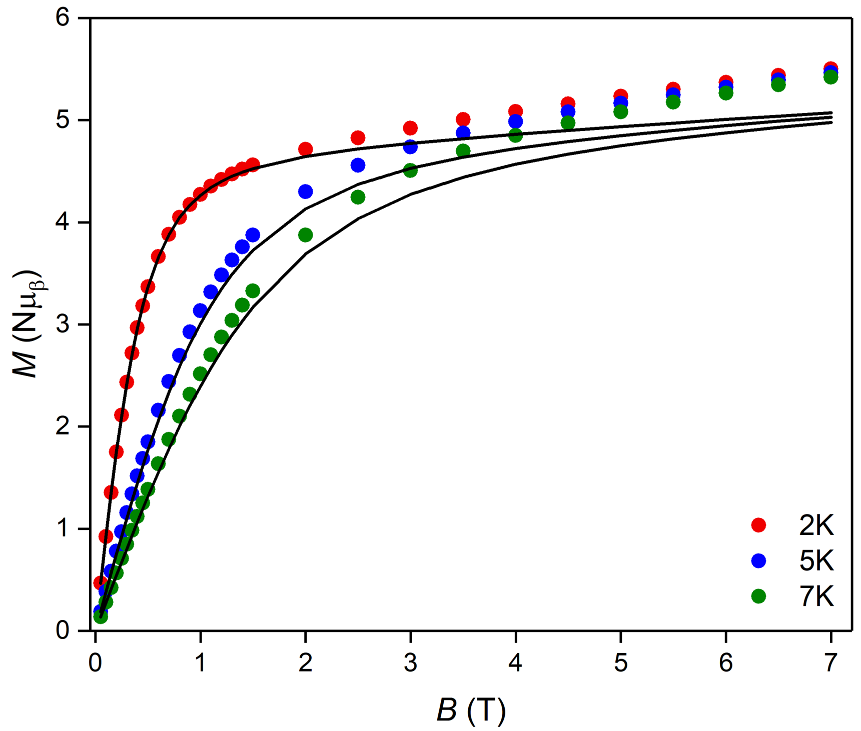

Figure S7. Magnetization (*M*) vs. magnetic field (*B*) plot for complex **P1·Dy** at 2, 5 and 7 K. Solid lines are fits.

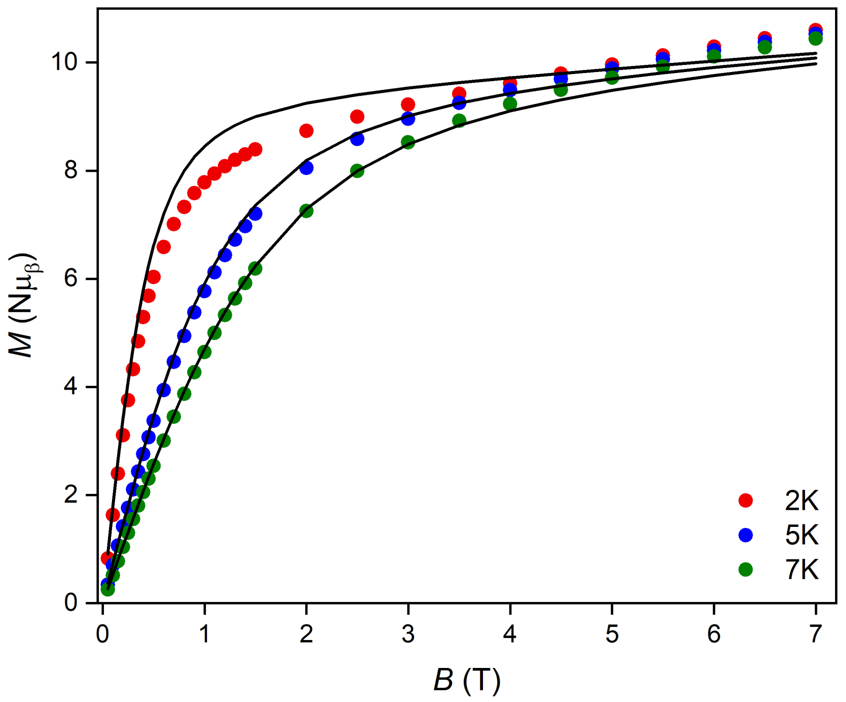

Figure S8. Magnetization (*M*) vs. magnetic field (*B*) plot for complex ***s*-P2·Dy$_2$** at 2, 5 and 7 K. Solid lines are fits.

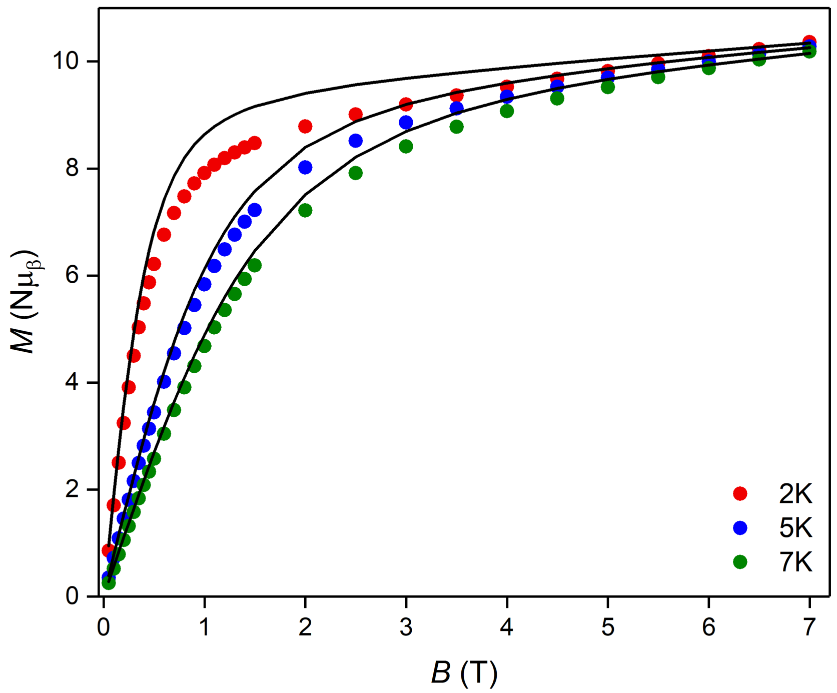

Figure S9. Magnetization (M) vs. magnetic field (B) plot for complex ***f*-P2-*Z*-Dy₂** at 2, 5 and 7 K. Solid lines are fits.

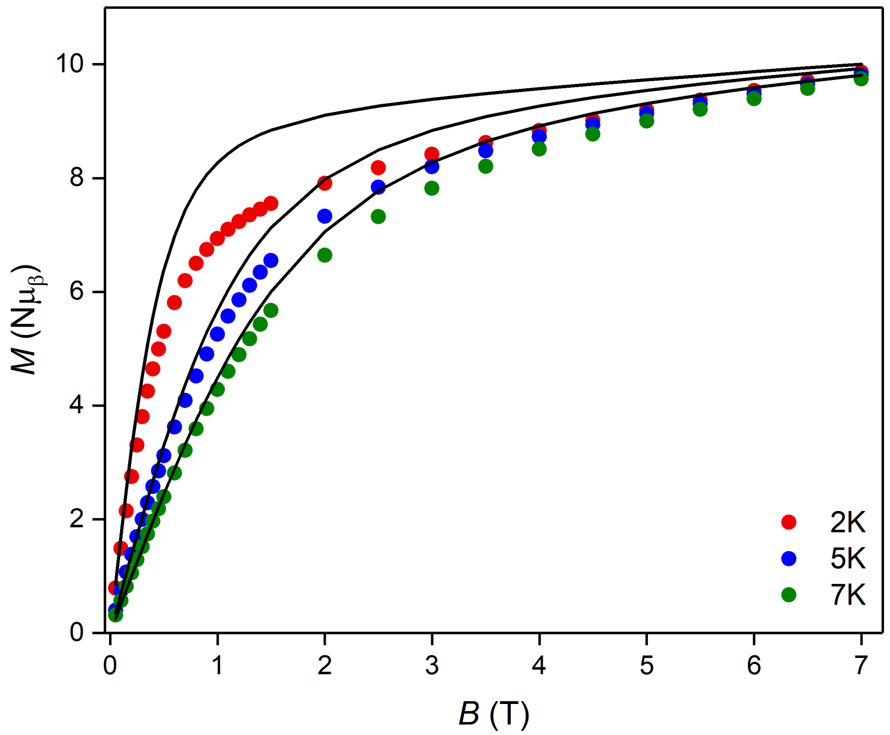

Figure S10. Magnetization (M) vs. magnetic field (B) plot for complex ***f*-P2-*E*-Dy₂** at 2, 5 and 7K. Solid lines are fits.

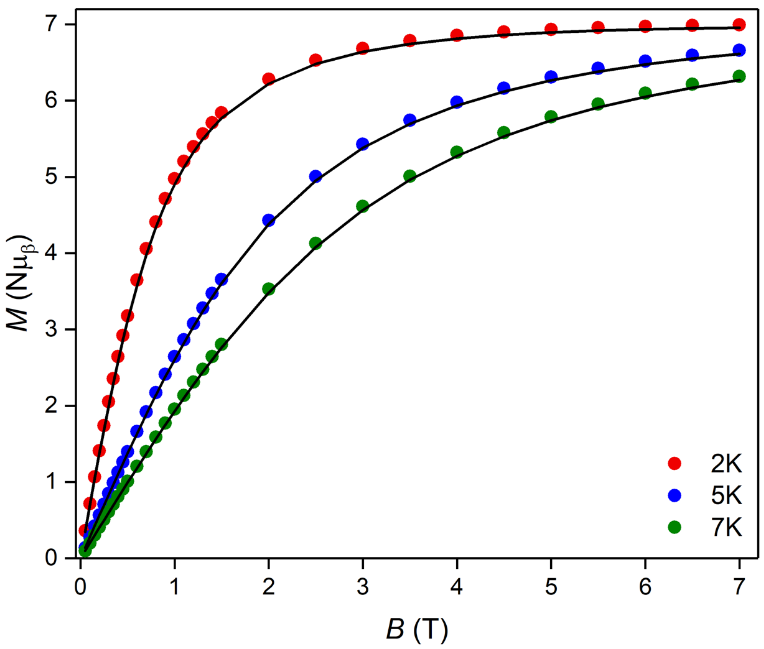

Figure S11. Magnetization (*M*) vs. magnetic field (*B*) plot for complex **P1·Gd** at 2, 5 and 7 K. Solid lines are fits.

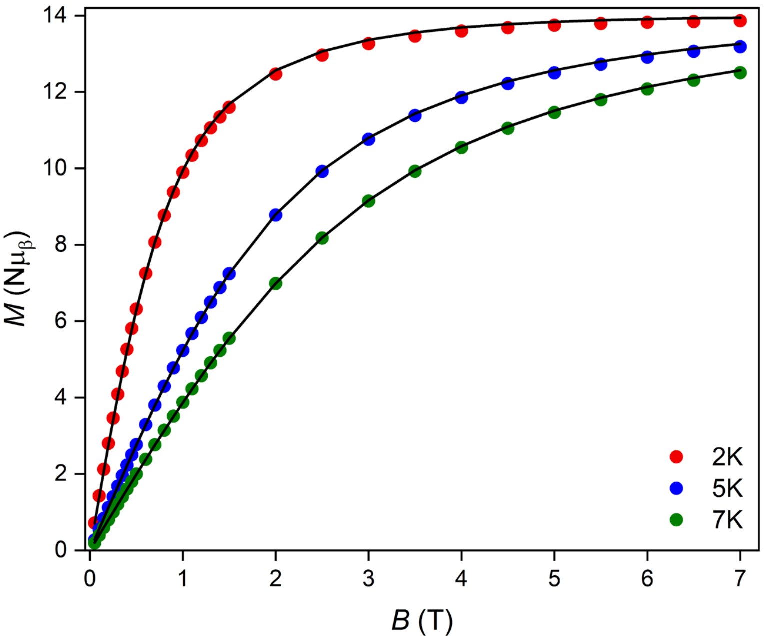

Figure S12. Magnetization (*M*) vs. magnetic field (*B*) plot for complex ***s*-P2·Gd$_2$** at 2, 5 and 7 K. Solid lines are fits.

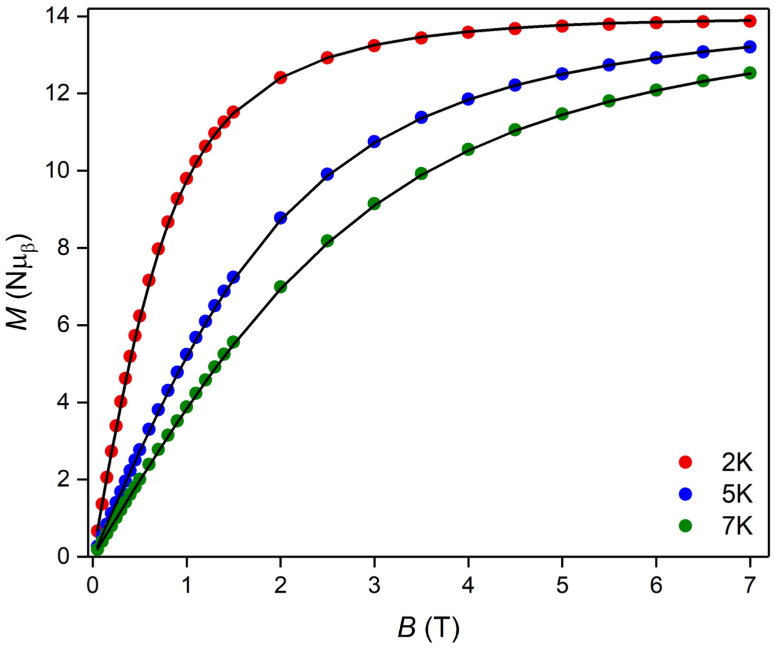

Figure S13. Magnetization (*M*) vs. magnetic field (*B*) plot for complex ***f*-P2-*Z*-Gd$_2$** at 2, 5 and 7 K. Solid lines are fits.

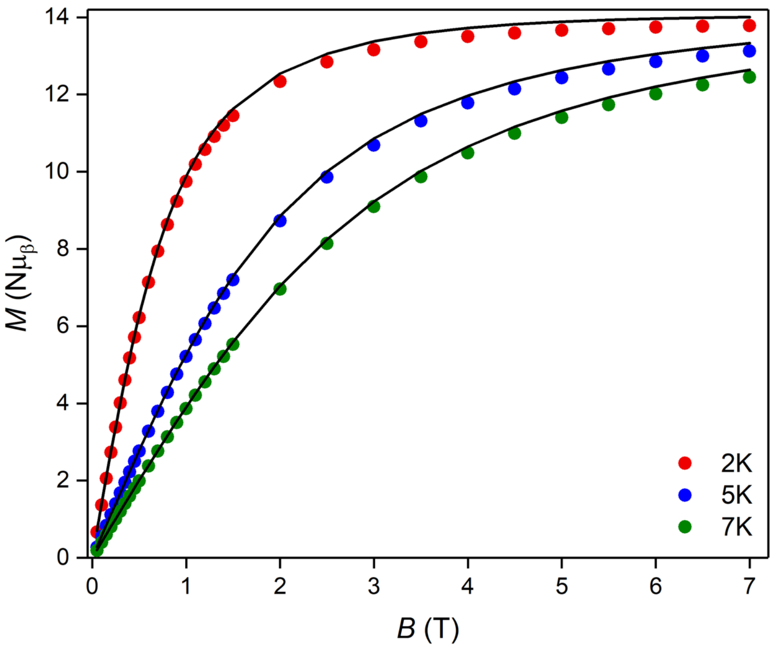

Figure S14. Magnetization (*M*) vs. magnetic field (*B*) plot for complex ***f*-P2-*E*-Gd$_2$** at 2, 5 and 7 K. Solid lines are fits.

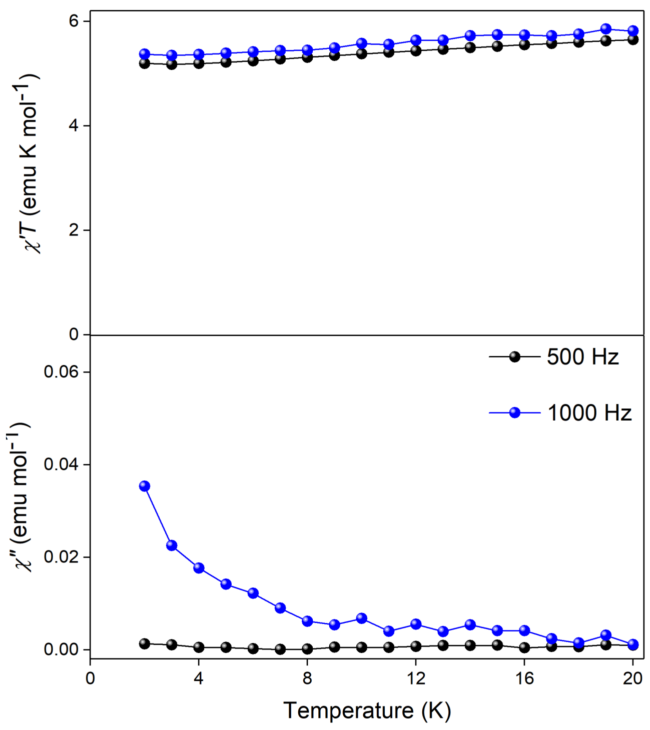

Figure S15. Temperature dependence of the in-phase $\chi' T$ product (top) and out-of-phase χ'' (bottom) *ac* susceptibility signals of **P1·Dy$_2$** in a 0.2 mT field oscillating at the indicated frequencies.

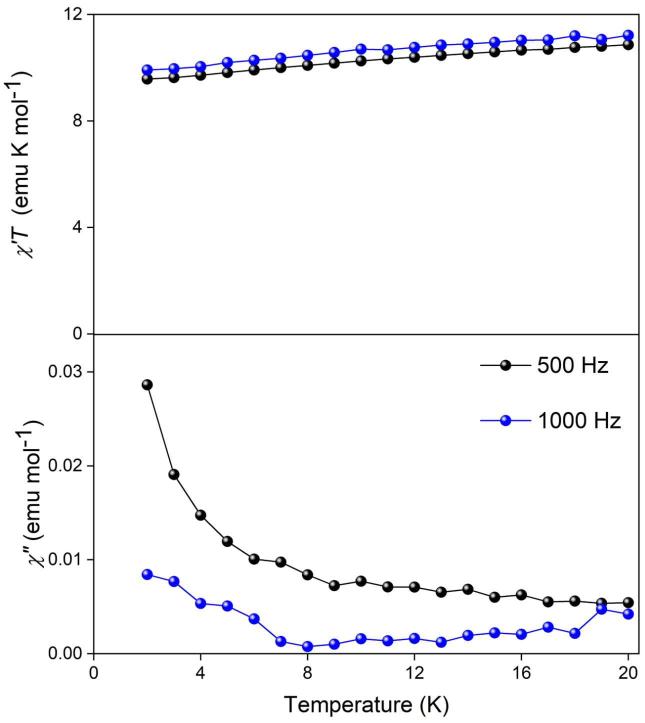

Figure S16. Temperature dependence of the in-phase $\chi' T$ product (top) and out-of-phase χ'' (bottom) *ac* susceptibility signals of ***s*-P2·Dy$_2$** in a 0.2 mT field oscillating at the indicated frequencies.

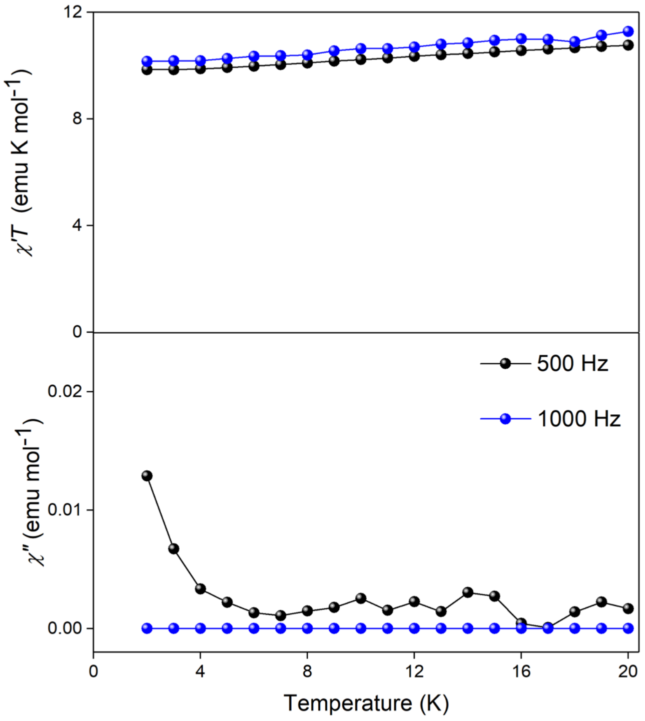

Figure S17. Temperature dependence of the in-phase $\chi' T$ product (top) and out-of-phase χ'' (bottom) *ac* susceptibility signals of ***f*-P2-*Z*-Dy₂** in a 0.2 mT field oscillating at the indicated frequencies.

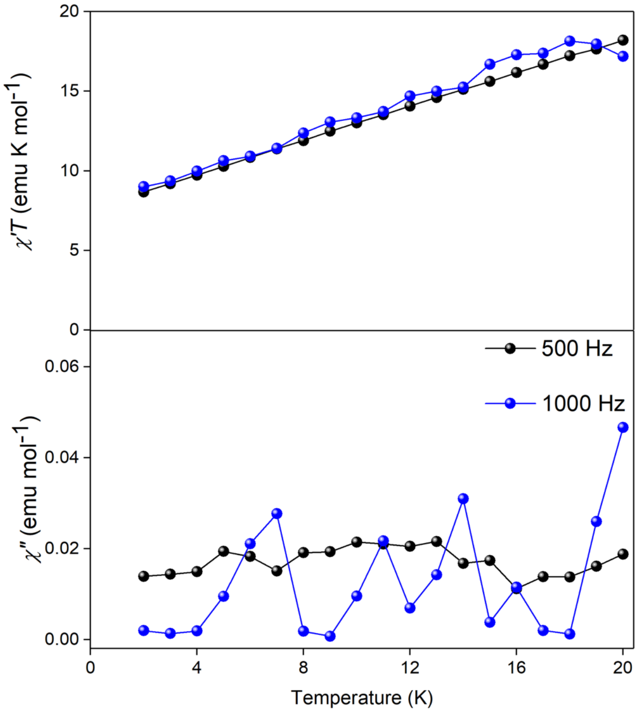

Figure S18. Temperature dependence of the in-phase $\chi' T$ product (top) and out-of-phase χ'' (bottom) *ac* susceptibility signals of ***f*-P2-*E*-Dy₂** in a 0.2 mT field oscillating at the indicated frequencies.

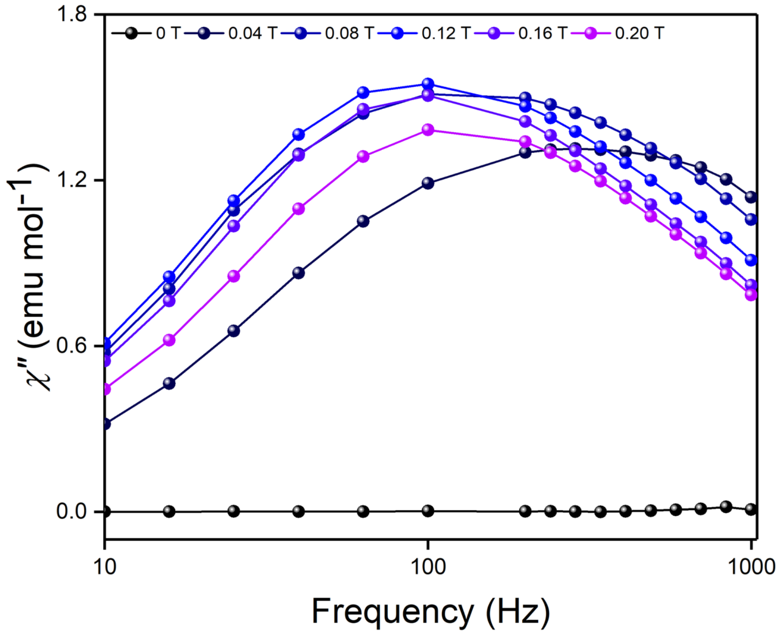

Figure S19. Out-of-phase susceptibility for **P1·Dy** in various applied *dc* fields at 2 K. Solid lines are guide for the eye.

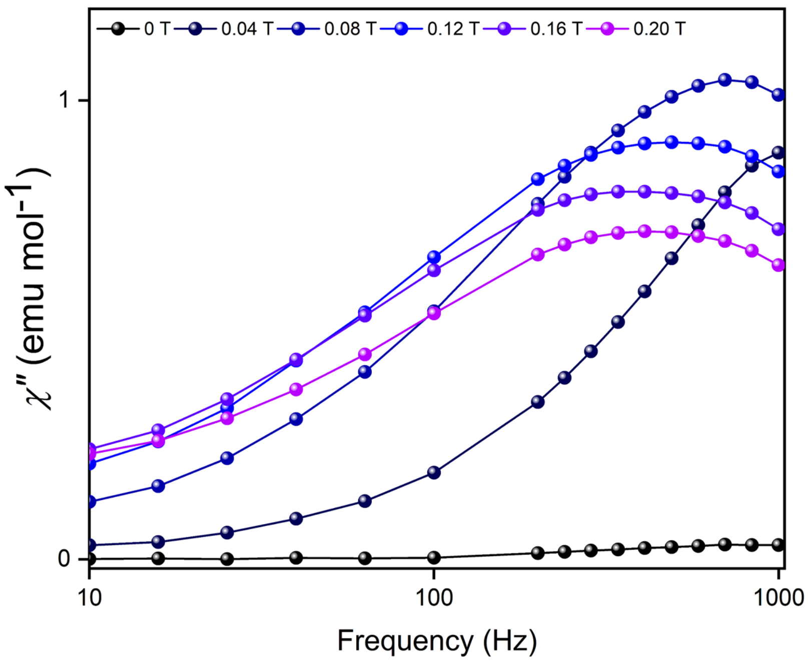

Figure S20. Out-of-phase susceptibility for ***s*-P2·Dy$_2$** in various applied *dc* fields at 2 K. Solid lines are guides for the eye.

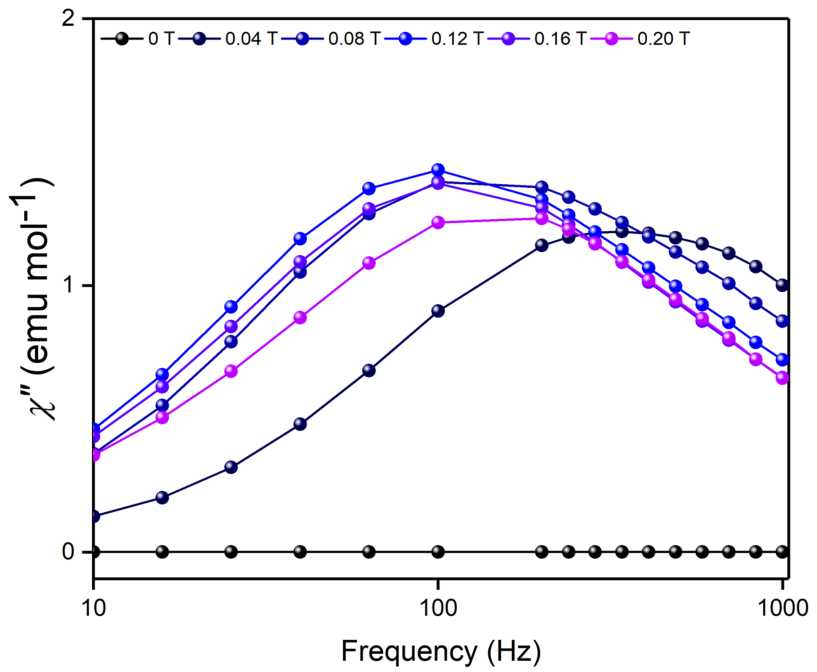

Figure S21. Out-of-phase susceptibility for ***f*-P2-*Z*-Dy$_2$** in various applied *dc* fields at 2 K. Solid lines are guides for the eye.

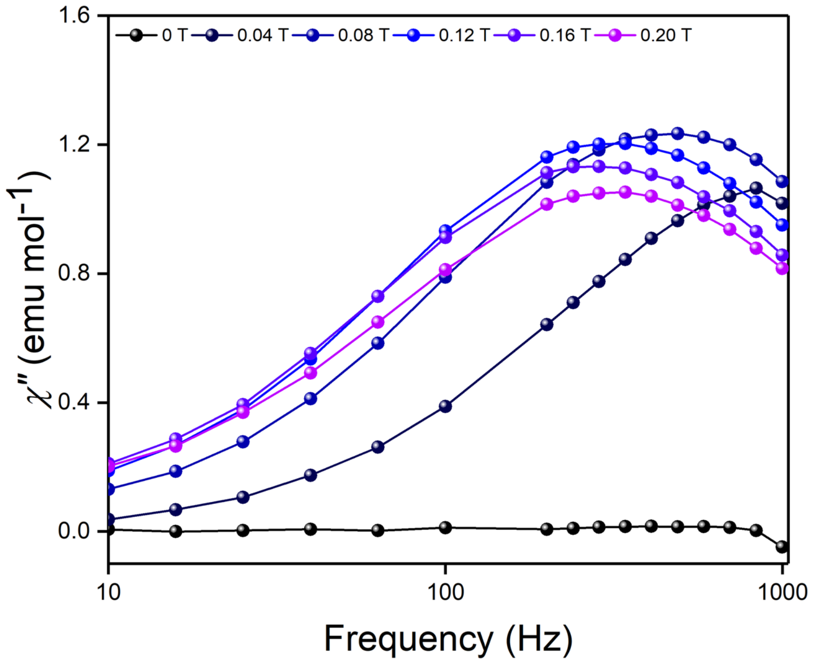

Figure S22. Out-of-phase susceptibility for ***f*-P2-*E*-Dy$_2$** in various applied *dc* fields at 2 K. Solid lines are guide for the eye.

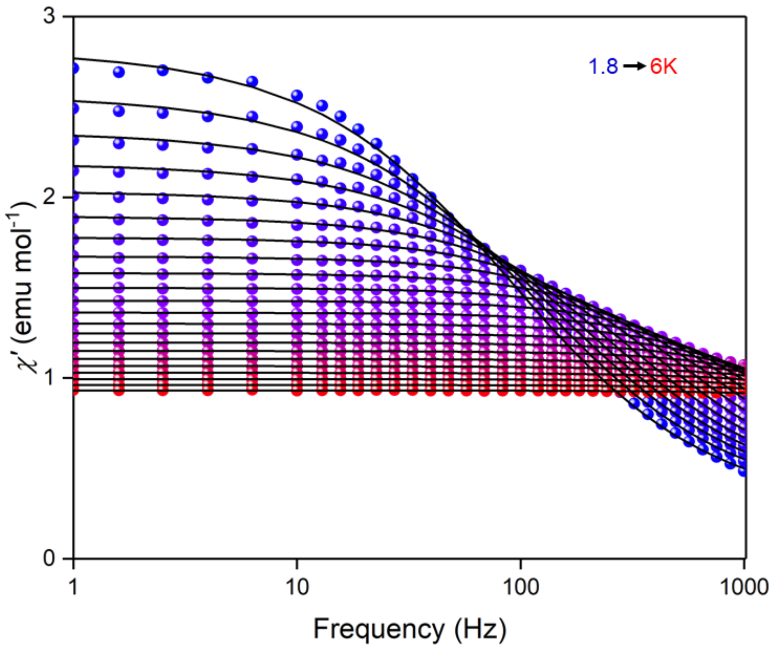

Figure S23. In-phase (χ') component of the magnetic susceptibility, under a 0.12 T *dc* field, for complex **P1·Dy**. Solid lines correspond to the best fit obtained with a generalized Debye model.

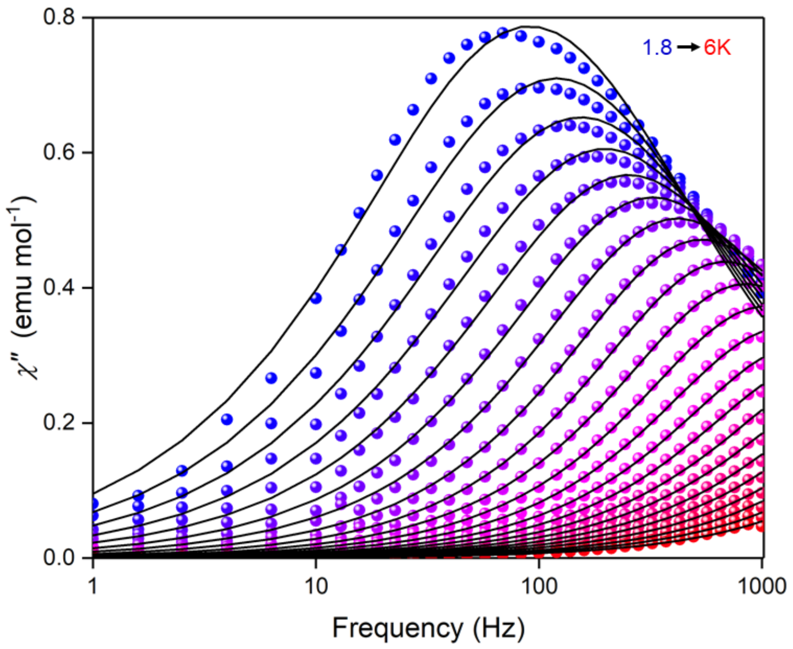

Figure S24. Out-of-phase (χ'') component of the magnetic susceptibility, under a 0.12 T *dc* field, for complex **P1·Dy**. Solid lines correspond to the best fit obtained with a generalized Debye model.

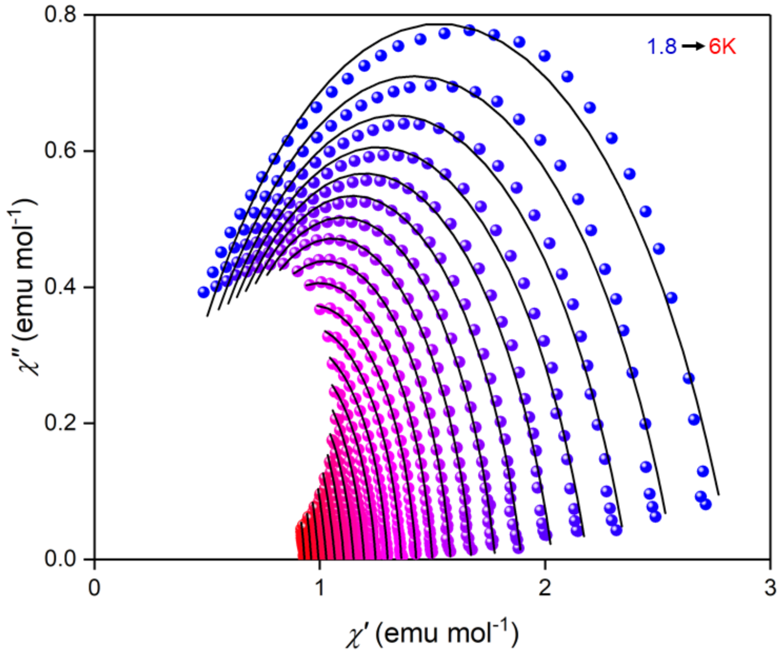

Figure S25. Argand plot for **P1·Dy** obtained using the *ac* susceptibility data in 0.12 T applied *dc* field. The solid lines correspond to the best fit obtained with a generalized Debye model.

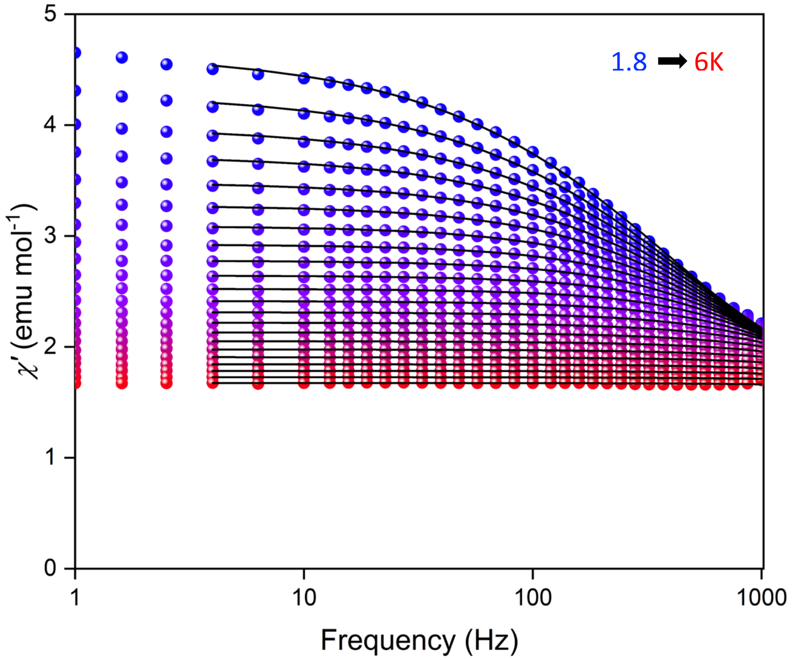

Figure S26. In-phase (χ') component of the magnetic susceptibility, under a 0.12 T *dc* field, for complex ***s*-P2·Dy$_2$**. Solid lines correspond to the best fit obtained with a generalized Debye model.

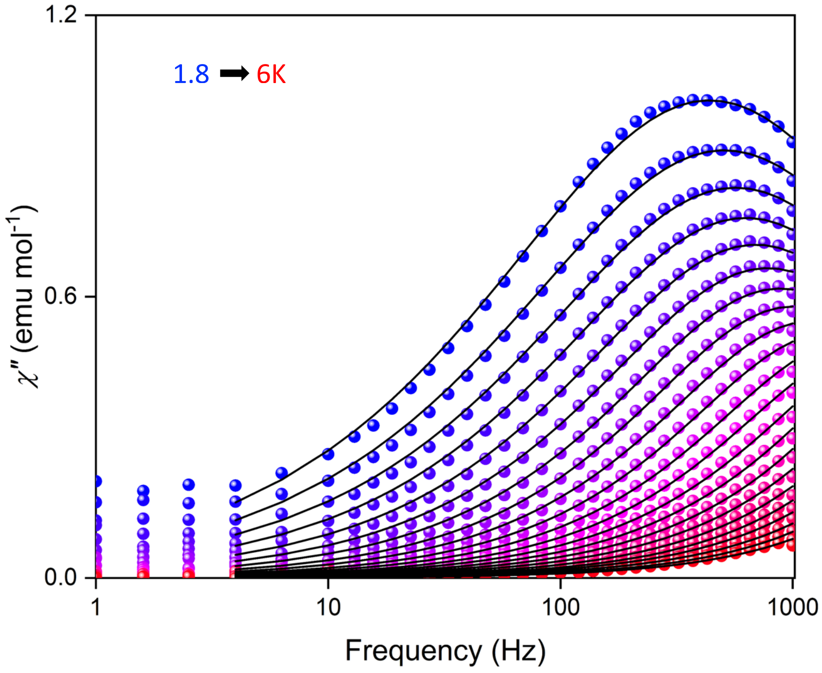

Figure S27. Out-of-phase (χ'') component of the magnetic susceptibility, under a 0.12 T *dc* field, for complex ***s*-P2·Dy$_2$**. Solid lines correspond to the best fit obtained with a generalized Debye model.

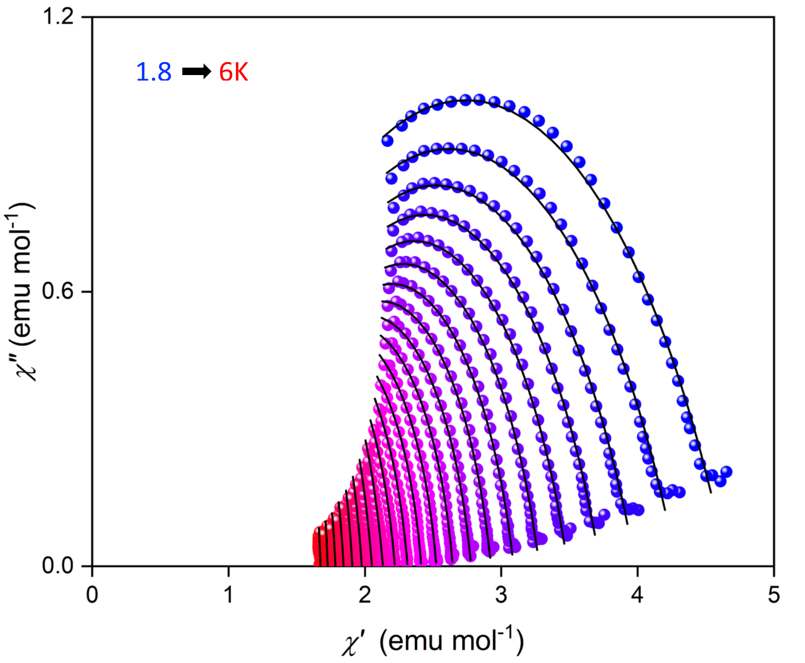

Figure S28. Argand plot for ***s*-P2·Dy$_2$** obtained using the *ac* susceptibility data in 0.12 T applied *dc* field. The solid lines correspond to the best fit obtained with a generalized Debye model.

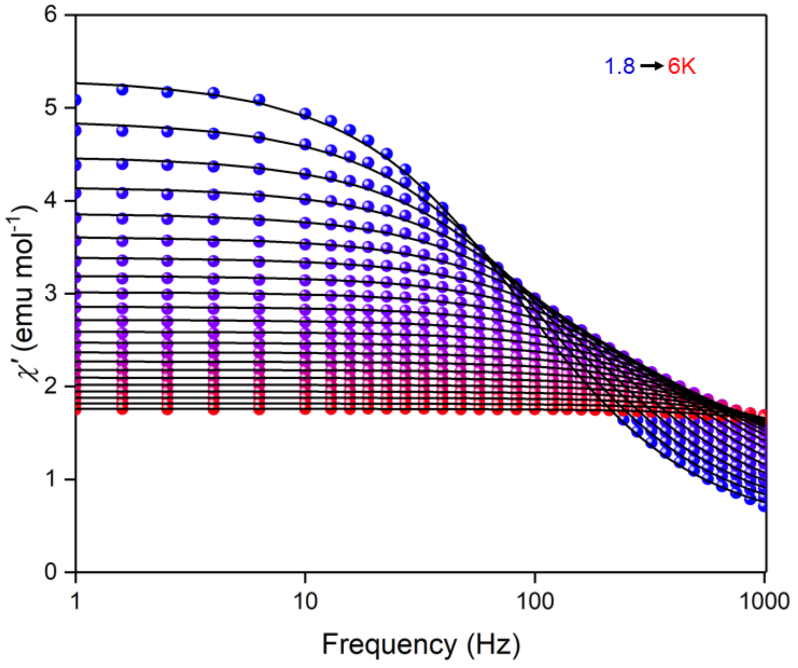

Figure S29. In-phase (χ') component of the magnetic susceptibility, under a 0.12 T *dc* field, for complex ***t*-P2-*Z*-Dy$_2$**. Solid lines correspond to the best fit obtained with a generalized Debye model.

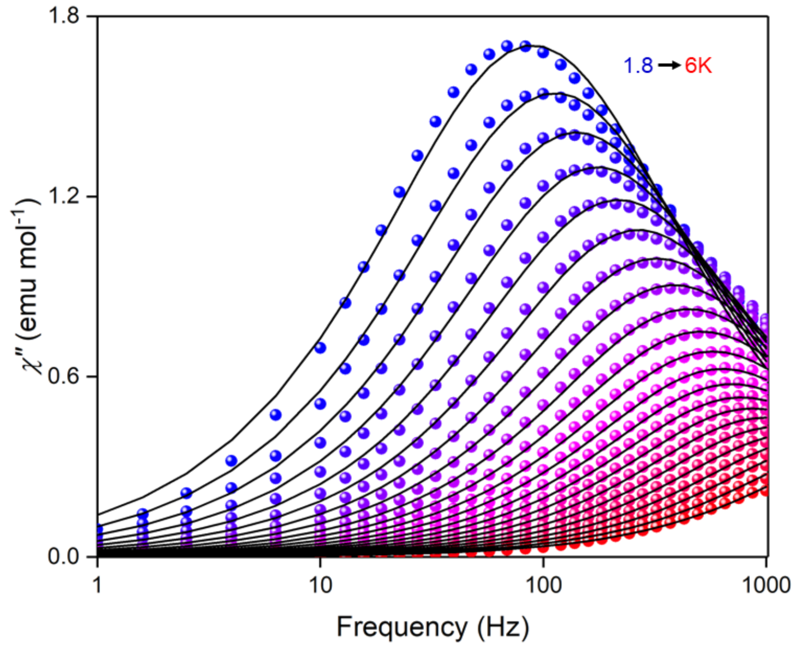

Figure S30. Out-of-phase (χ'') component of the magnetic susceptibility, under a 0.12 T *dc* field, for complex ***t*-P2-*Z*-Dy$_2$**. Solid lines correspond to the best fit obtained with a generalized Debye model.

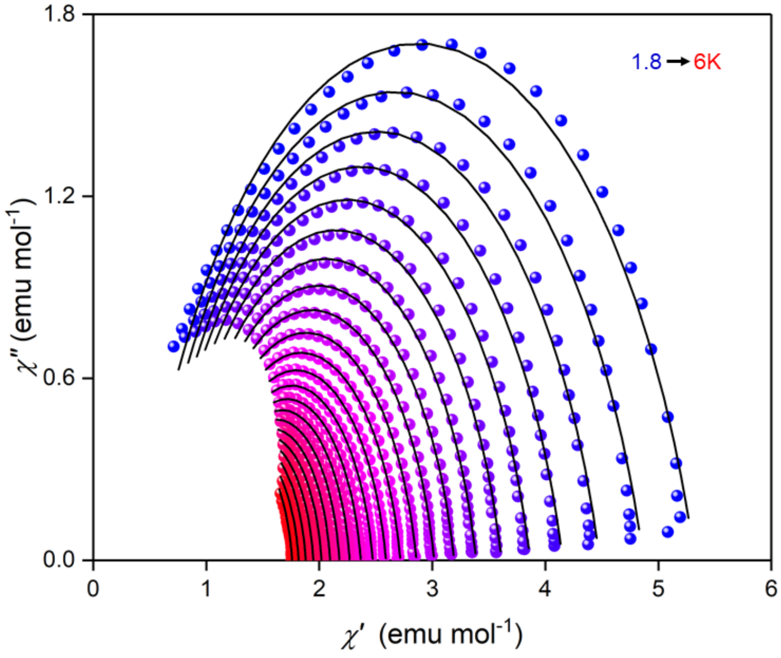

Figure S31. Argand plot for ***f*-P2-*Z*-Dy$_2$** obtained using the *ac* susceptibility data in 0.12 T applied *dc* field. The solid lines correspond to the best fit obtained with a generalized Debye model.

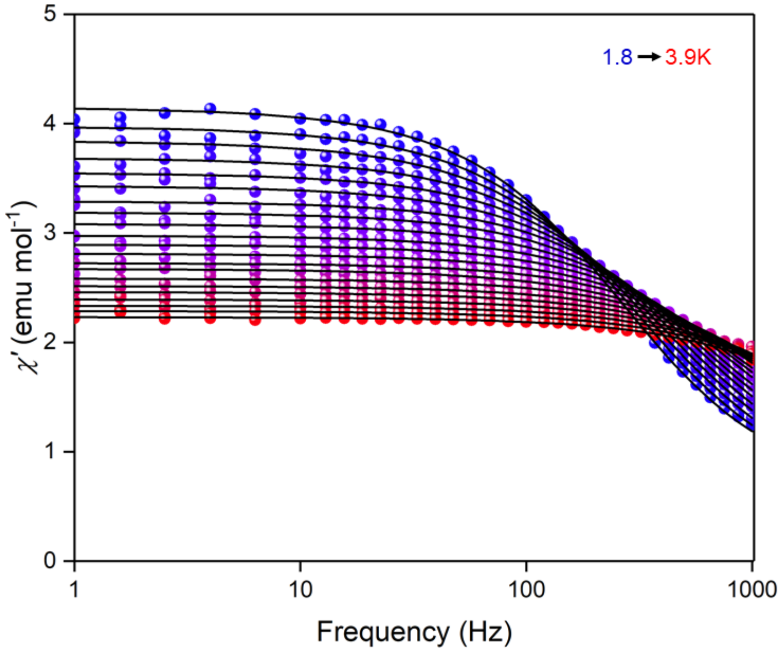

Figure S32. In-phase (χ′) component of the magnetic susceptibility, under a 0.12 T *dc* field, for complex ***f*-P2-*E*-Dy$_2$**. Solid lines correspond to the best fit obtained with a generalized Debye model.

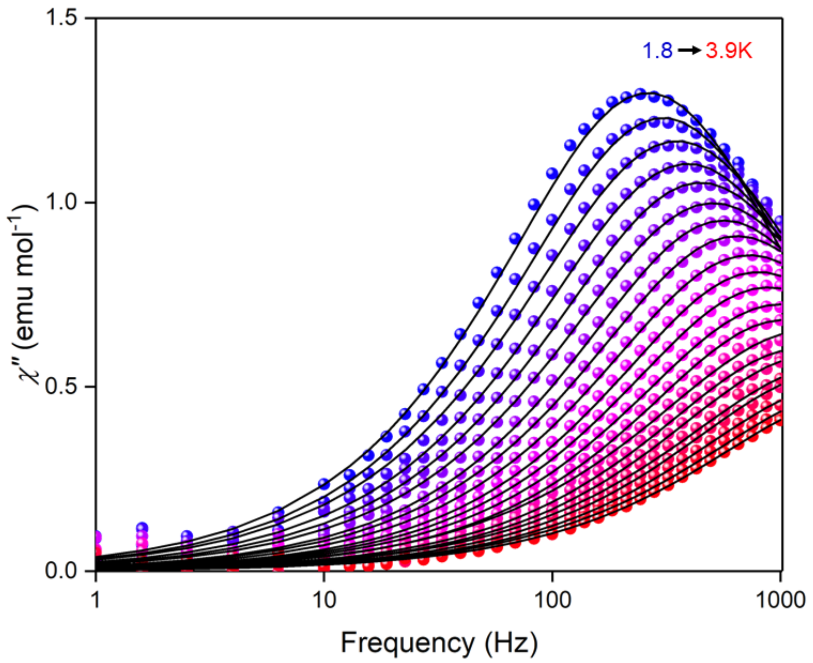

Figure S33. Out-of-phase (χ'') component of the magnetic susceptibility, under a 0.12 T *dc* field, for complex ***f*-P2-*E*-Dy₂**. Solid lines correspond to the best fit obtained with a generalized Debye model.

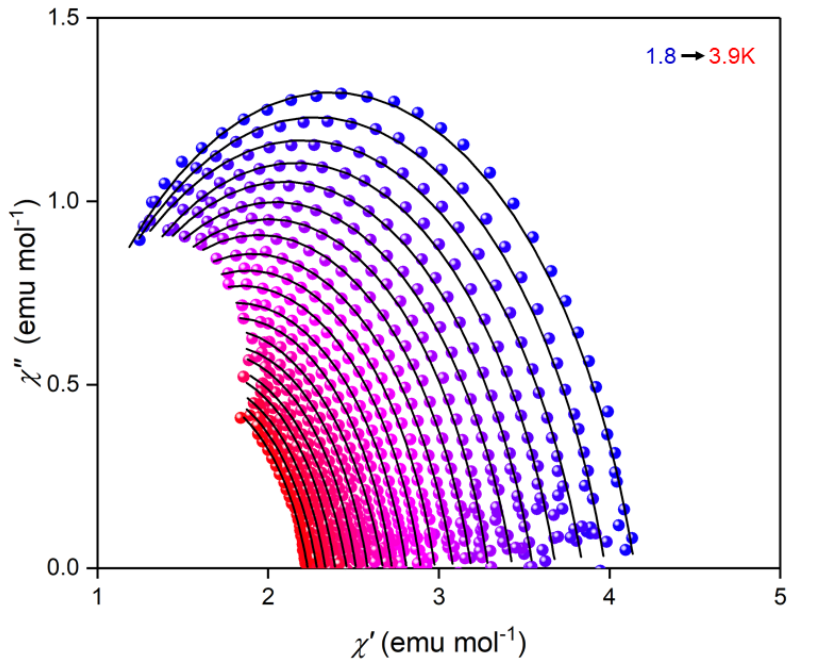

Figure S34. Argand plot for ***f*-P2-*E*-Dy$_2$** obtained using the *ac* susceptibility data in 0.12 T applied *dc* field. The solid lines correspond to the best fit obtained with a generalized Debye model.

Table S3. Argand fit values of **P1·Dy** between 1.8–6.0 K under an applied *dc* field of 0.12 T and an *ac* field of 0.2 mT.

T / K	χ_s / emu mol^{-1}	χ_t / cm^3 mol^{-1}	τ / s	α	Residual
1.9	5.89E-01	4.15E+00	6.05E-04	1.98E-01	5.54E-02
2.0	5.81E-01	3.98E+00	5.21E-04	2.02E-01	3.40E-02
2.1	5.62E-01	3.84E+00	4.53E-04	2.13E-01	3.74E-02
2.2	6.31E-01	3.69E+00	4.06E-04	2.03E-01	2.61E-02
2.3	6.40E-01	3.55E+00	3.57E-04	2.02E-01	1.52E-02
2.4	6.52E-01	3.43E+00	3.14E-04	2.07E-01	2.30E-02
2.5	7.21E-01	3.29E+00	2.83E-04	1.88E-01	2.46E-02
2.6	6.97E-01	3.19E+00	2.46E-04	1.98E-01	2.47E-02
2.7	7.28E-01	3.08E+00	2.18E-04	1.99E-01	2.49E-02
2.8	7.88E-01	2.98E+00	2.00E-04	1.88E-01	1.96E-02
2.9	8.02E-01	2.89E+00	1.80E-04	1.92E-01	1.15E-02
3.0	8.15E-01	2.81E+00	1.60E-04	2.01E-01	2.25E-02
3.1	8.84E-01	2.72E+00	1.51E-04	1.88E-01	2.60E-02
3.2	7.72E-01	2.67E+00	1.20E-04	2.33E-01	1.43E-02
3.3	9.18E-01	2.58E+00	1.25E-04	1.99E-01	1.74E-02
3.4	9.03E-01	2.51E+00	1.12E-04	1.99E-01	1.16E-02
3.5	8.57E-01	2.46E+00	9.35E-05	2.25E-01	1.35E-02
3.6	9.15E-01	2.39E+00	9.62E-05	2.00E-01	6.90E-03
3.7	9.31E-01	2.34E+00	8.66E-05	2.06E-01	2.47E-02
3.8	8.85E-01	2.29E+00	7.45E-05	2.21E-01	1.02E-02
3.9	9.03E-01	2.23E+00	7.21E-05	2.12E-01	8.68E-03

Table S4. Argand fit values of ***s*-P2·Dy$_2$** between 1.8–6.0 K under an applied *dc* field of 0.12 T and an *ac* field of 0.2 mT.

T / K	χ_s / emu mol^{-1}	χ_t / emu mol^{-1}	τ / s	α	Residual
1.8	8.22E-01	4.66E+00	3.63E-04	2.38E-01	5.01E-02
2.0	9.42E-01	4.29E+00	3.14E-04	2.36E-01	4.35E-02
2.2	1.06E+00	3.98E+00	2.80E-04	2.34E-01	2.86E-02
2.4	1.15E+00	3.73E+00	2.53E-04	2.31E-01	2.37E-02
2.6	1.24E+00	3.49E+00	2.30E-04	2.82E-01	1.66E-02
2.8	1.28E+00	3.28E+00	2.05E-04	2.55E-01	1.39E-02
3.0	1.31E+00	3.09E+00	1.81E-04	2.28E-01	1.08E-02
3.2	1.33E+00	2.92E+00	1.60E-04	2.00E-01	9.40E-03
3.4	1.30E+00	2.78E+00	1.33E-04	1.87E-01	8.80E-03
3.6	1.28E+00	2.64E+00	1.12E-04	1.70E-01	5.97E-03
3.8	1.20E+00	2.52E+00	8.85E-05	1.66E-01	4.97E-03
4.0	1.15E+00	2.41E+00	7.21E-05	1.57E-01	3.56E-03
4.2	9.67E-01	2.31E+00	4.94E-05	1.67E-01	3.74E-03
4.4	7.85E-01	2.22E+00	3.49E-05	1.67E-01	3.29E-03
4.6	4.02E-01	2.13E+00	2.12E-05	1.68E-01	2.42E-03
4.8	1.28E-10	2.05E+00	1.35E-05	1.62E-01	2.23E-03
5.0	2.59E-10	1.98E+00	1.19E-05	1.40E-01	3.23E-03
5.2	3.46E-10	1.91E+00	1.07E-05	1.17E-01	3.18E-03
5.4	4.31E-10	1.84E+00	9.28E-06	1.11E-01	2.52E-03
5.6	6.04E-10	1.78E+00	8.17E-06	9.17E-02	3.15E-03
5.8	8.61E-10	1.73E+00	7.68E-06	6.14E-02	3.11E-03
6.0	2.40E-09	1.67E+00	6.74E-06	5.57E-02	2.52E-03

Table S5. Argand fit values of ***f*-P2-*Z*-Dy$_2$** between 1.8–6.0 K under an applied *dc* field of 0.12 T and an *ac* field of 0.2 mT.

T / K	χ_s / emu mol^{-1}	χ_t / emu mol^{-1}	τ / s	α	Residual
1.8	4.18E-01	5.32E+00	1.78E-03	2.27E-01	1.54E-02
2.0	4.81E-01	4.87E+00	1.41E-03	2.19E-01	1.09E-02
2.2	5.38E-01	4.48E+00	1.13E-03	2.08E-01	8.81E-02
2.4	5.86E-01	4.15E+00	9.08E-04	1.99E-01	6.71E-02
2.6	6.33E-01	3.87E+00	7.33E-04	1.92E-01	5.17E-02
2.8	6.82E-01	3.61E+00	5.96E-04	1.86E-01	3.80E-02
3.0	7.37E-01	3.39E+00	4.92E-04	1.81E-01	2.82E-02
3.2	7.94E-01	3.19E+00	4.13E-04	1.77E-01	2.11E-02
3.4	8.52E-01	3.02E+00	3.54E-04	1.71E-01	1.65E-02
3.6	9.08E-01	2.86E+00	3.10E-04	1.65E-01	1.40E-02
3.8	9.63E-01	2.72E+00	2.77E-04	1.57E-01	1.13E-02
4.0	1.01E+00	2.59E+00	2.50E-04	1.48E-01	9.24E-03
4.2	1.04E+00	2.47E+00	2.27E-04	1.37E-01	8.16E-03
4.4	1.07E+00	2.37E+00	2.06E-04	1.27E-01	6.56E-03
4.6	1.08E+00	2.27E+00	1.83E-04	1.17E-01	5.82E-03
4.8	1.08E+00	2.18E+00	1.62E-04	1.08E-01	3.87E-03
5.0	1.08E+00	2.10E+00	1.41E-04	1.01E-01	3.92E-03
5.2	1.07E+00	2.02E+00	1.19E-04	9.71E-02	3.17E-03
5.4	1.03E+00	1.95E+00	9.59E-05	9.91E-02	2.98E-03
5.6	9.82E-01	1.88E+00	7.64E-05	9.94E-02	2.04E-03
5.8	9.42E-01	1.82E+00	6.01E-05	1.02E-01	1.50E-03
6.0	8.21E-01	1.76E+00	4.15E-05	1.13E-01	1.17E-03

Table S6. Argand fit values of ***f*-P2-*E*-Dy₂** between 1.8–3.9 K under an applied *dc* field of 0.12 T and an *ac* field of 0.2 mT.

T / K	χ_s / emu mol^{-1}	χ_t / emu mol^{-1}	τ / s	α	Residual
1.9	5.89E-01	4.15E+00	6.05E-04	1.98E-01	5.54E-02
2.0	5.81E-01	3.98E+00	5.21E-04	2.02E-01	3.40E-02
2.1	5.62E-01	3.84E+00	4.53E-04	2.13E-01	3.74E-02
2.2	6.31E-01	3.69E+00	4.06E-04	2.03E-01	2.61E-02
2.3	6.40E-01	3.55E+00	3.57E-04	2.02E-01	1.52E-02
2.4	6.52E-01	3.43E+00	3.14E-04	2.07E-01	2.30E-02
2.5	7.21E-01	3.29E+00	2.83E-04	1.88E-01	2.46E-02
2.6	6.97E-01	3.19E+00	2.46E-04	1.98E-01	2.47E-02
2.7	7.28E-01	3.08E+00	2.18E-04	1.99E-01	2.49E-02
2.8	7.88E-01	2.98E+00	2.00E-04	1.88E-01	1.96E-02
2.9	8.02E-01	2.89E+00	1.80E-04	1.92E-01	1.15E-02
3.0	8.15E-01	2.81E+00	1.60E-04	2.01E-01	2.25E-02
3.1	8.84E-01	2.72E+00	1.51E-04	1.88E-01	2.60E-02
3.2	7.72E-01	2.67E+00	1.20E-04	2.33E-01	1.43E-02
3.3	9.18E-01	2.58E+00	1.25E-04	1.99E-01	1.74E-02
3.4	9.03E-01	2.51E+00	1.12E-04	1.99E-01	1.16E-02
3.5	8.57E-01	2.46E+00	9.35E-05	2.25E-01	1.35E-02
3.6	9.15E-01	2.39E+00	9.62E-05	2.00E-01	6.90E-03
3.7	9.31E-01	2.34E+00	8.66E-05	2.06E-01	2.47E-02
3.8	8.85E-01	2.29E+00	7.45E-05	2.21E-01	1.02E-02
3.9	9.03E-01	2.23E+00	7.21E-05	2.12E-01	8.68E-03

8. Electron Spin Resonance Spectroscopy (EPR) data

EPR measurements were carried out on a Bruker ElexSys E580 system, operating at Q-band frequency (~33.5–34.0 GHz). The sample temperature was maintained with an Oxford Instruments CF9350 cryostat and controlled with an Oxford Instruments MercuryiTC. The sample was a 1 mM solution of the Gd porphyrin dissolved in CS_2 in a 1.6 mm OD EPR glass tube with very low background, and inserted into a Bruker EN510702 resonator.

Hahn-echo sequence to determine phase-memory times T_m

Phase-memory times, T_m, were obtained using a Hahn-echo sequence, depicted in Fig. S35. In the rotating frame approximation, a $\pi/2$-pulse brings spins, originally aligned in *z*-direction, into the *xy*-plane, where they freely precess. A π-pulse after a time delay τ adds a phase, so that spins recombine after a time-delay of 2τ (Fig. S35a). The overall echo is, however, smaller due to dephasing. Dephasing occurs via interaction of the spins with their local environment, e.g. with the nuclear spin bath or other spin centers. By varying the time delay τ, the echo signal $Y(\tau)$ decays, which is phenomenologically described with a stretched exponential (Fig. S35b):

$$Y(\tau) = Y_0 \exp\left(-\frac{2\tau}{T_m}\right)^x, \qquad (1)$$

where Y_0 corresponds to the echo amplitude at $\tau = 0$ and x is the scaling exponent. For the samples of study, the decay follows a monoexponential law ($x = 1$), as shown in Fig. S35c. The phase-memory time corresponds to the decay constant and can thus be extracted from the fit. In our experiments, we set the duration of the $\pi/2$ and π pulse to 16 ns and 32 ns, respectively. A 2-step phase cycling scheme was employed. Fig. S36 shows the temperature dependence of T_m for the three Gd dimers taken at different peaks of the spectrum (Fig. S37).

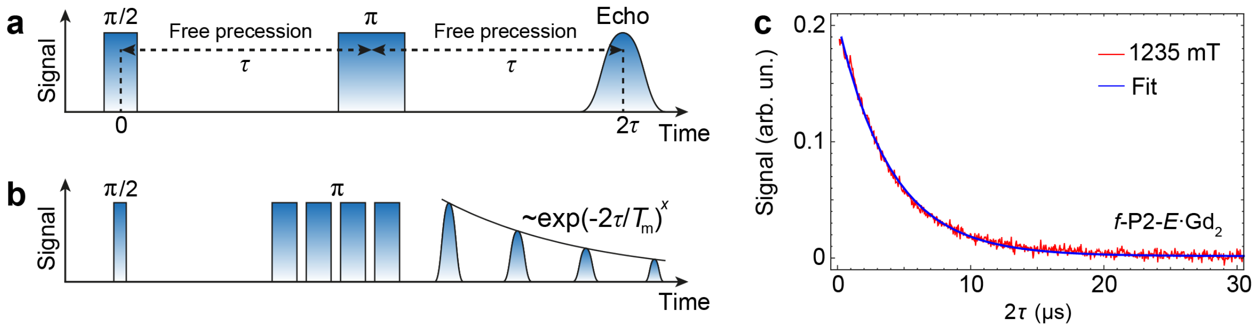

Figure S35. a) Hahn-echo pulse sequence. b) The echo signal decreases exponentially when increasing the time delay τ. c) Experimental data taken at a field of 1235 mT on ***f*-P2-*E*·Gd$_2$**. The data were fitted using a monoexponential ($x = 1$) decay function.

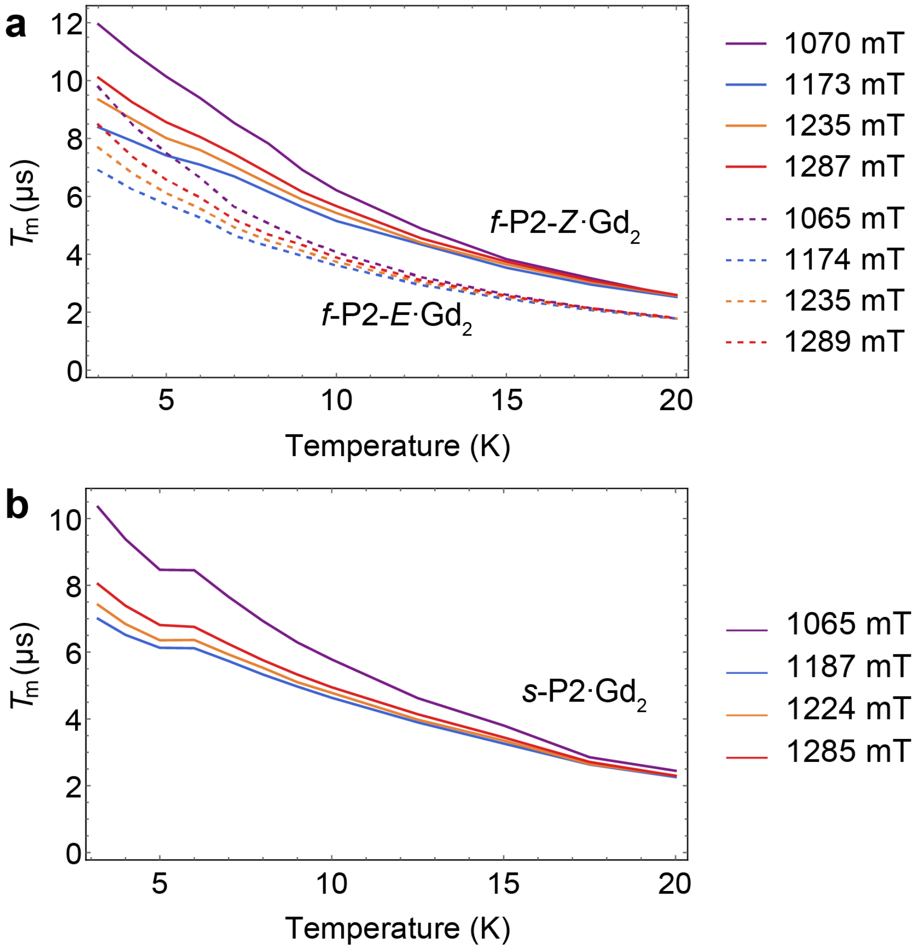

Figure S36. Temperature dependence of T_m of ***f*-P2-*Z*·Gd₂** (solid lines) and ***f*-P2-*E*·Gd₂** (dashed lines) in (a), and of ***s*-P2·Gd₂** in (b), obtained at fields corresponding to the four largest peaks of the corresponding EDFS spectrum (see Fig. S37).

Echo-detected field-swept EPR (EDFS)

The base of the EDFS technique is the Hahn-echo sequence. For each field, we measure the Hahn-echo signal for a fixed time-delay τ. The result is a microwave absorption spectrum. The field was swept between 0 T and 1.45 T. The 5 K Q-band EDFS spectra for ***f*-P2-*Z*·Gd₂**, ***f*-P2-*E*·Gd₂** and ***s*-P2·Gd₂** are shown in Fig. S37.

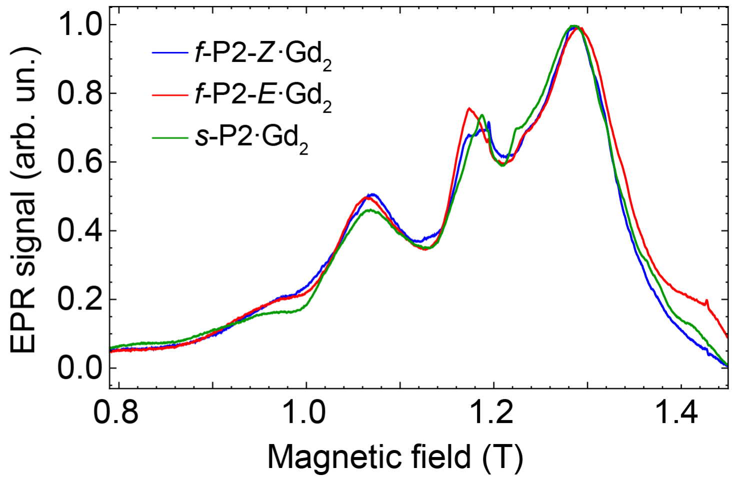

Figure S37. EDFS spectra of ***f*-P2-*Z*·Gd_2**, ***f*-P2-*E*·Gd_2** and ***s*-P2·Gd_2** obtained at 5 K and Q-band frequency. No major spectral features were observed below 0.8 T and this region is therefore omitted.

Fitting of the EDFS spectra

Spectral fits were obtained via EasySpin [4]. In our model, we account for the zero-field splitting, ZFS, using axial and transverse parameters *D* and *E*, respectively, an isotropic *g*-factor, and an isotropic exchange coupling *J* where appropriate:

$$H = \mu_B \boldsymbol{B} \cdot g \cdot \boldsymbol{S} + D\left(\boldsymbol{S}_z^2 - \frac{S(S+1)}{3}\right) + E\left(\boldsymbol{S}_x^2 - \boldsymbol{S}_y^2\right) - 2J(\boldsymbol{S}_1 \cdot \boldsymbol{S}_2), \qquad (2)$$

where μ_B corresponds to the Bohr magneton, $\boldsymbol{S}_{(x,y,z)}$ are the spin operators, and $\boldsymbol{S}_1 \cdot \boldsymbol{S}_2$ corresponds to the spin operator product of the two spin centers of the dimer. The linewidth is generated by a strain of the ZFS parameters. Fig. S38 shows the spectrum and the corresponding fit for **P1·Gd**, Fig. S39–S41 for ***f*-P2-*Z*·Gd_2**, ***f*-P2-*E*·Gd_2** and ***s*-P2·Gd_2**, respectively. The fit parameters are given in Table S7. In the fit, we assumed the background to be zero, meaning that all values are larger than 0. We note that we used an average lineshape given by the ZFS strains for all the transitions, giving rise to some lineshape deviations from the real data, but this should not significantly affect the fitted ZFS parameters. The couplings in both dimers are weak and antiferromagnetic ($J < 0$). Figure S38 shows the spectral decomposition of the single transitions in **P1·Gd**, using the fitted parameters. Orientational averaging of the different transitions (with their corresponding absorption strengths) lead to the powder spectrum observed in Figure S38. It can be directly seen that the peaks in Figure S38 cannot be directly linked to one particular transition, except for the sharp peak around 1.235 T.

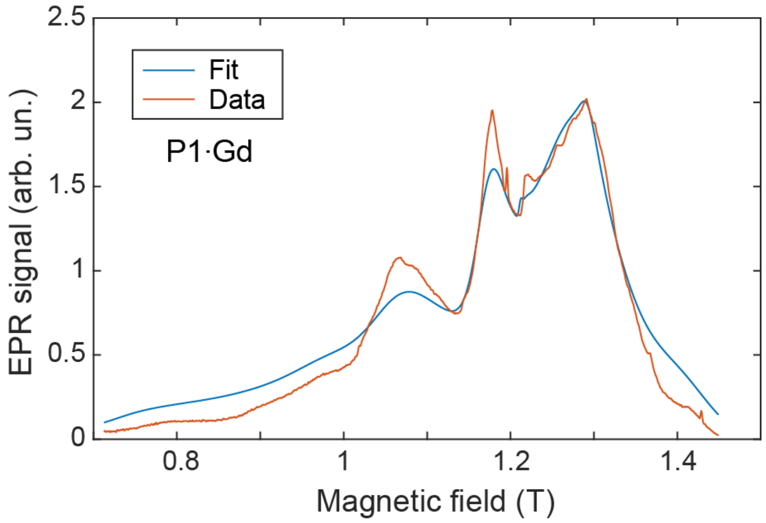

Figure S38. EDFS spectrum of the **P1·Gd** (orange) and its best fit (blue).

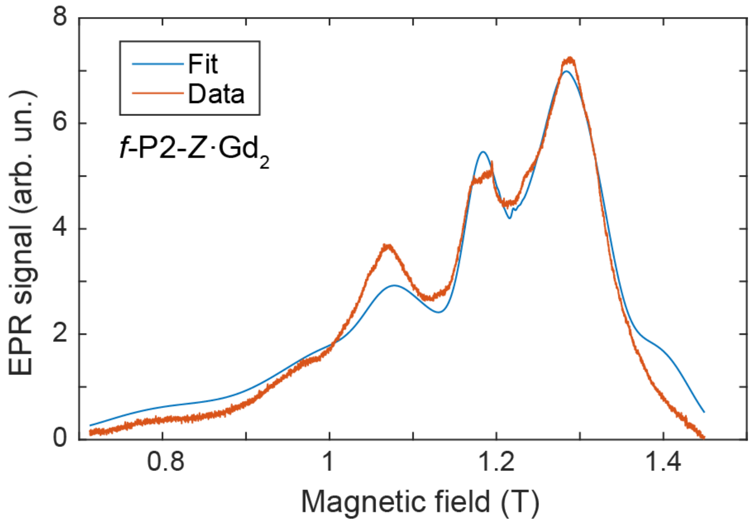

Figure S39. EDFS spectrum of ***f*-P2-*Z*·Gd$_2$** (orange) and its best fit (blue).

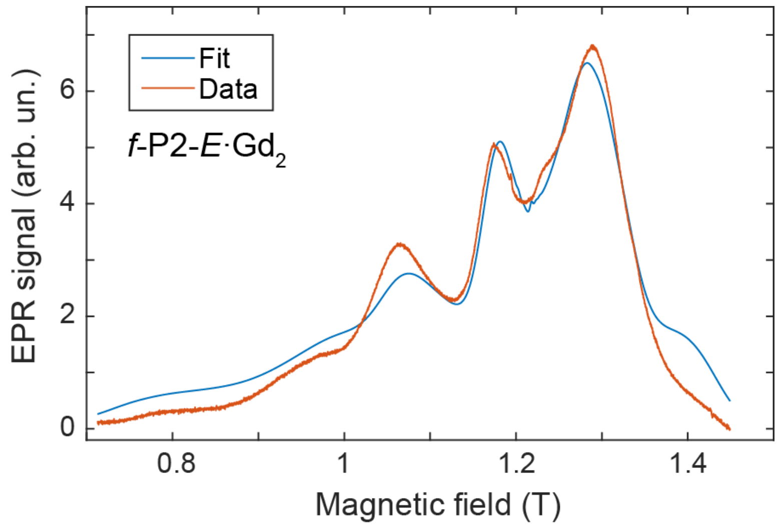

Figure S40. EDFS spectrum of ***f*-P2-*E*·Gd₂** (orange) and its best fit (blue).

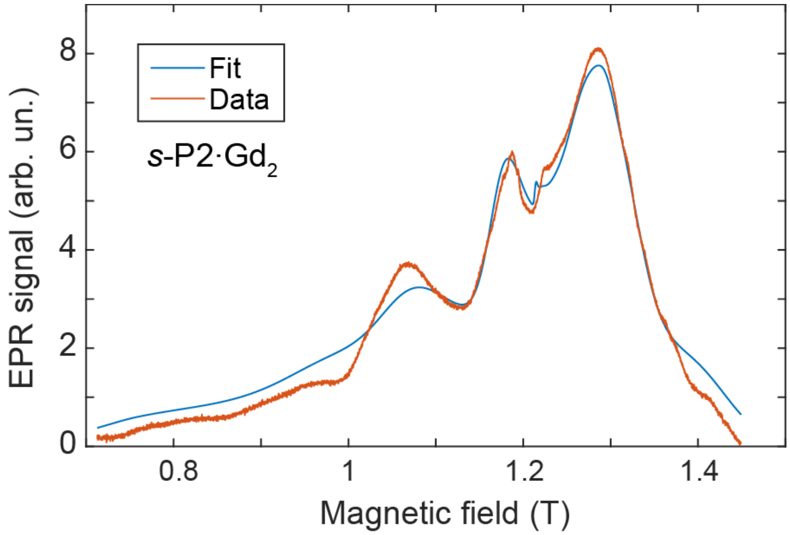

Figure S41. EDFS spectrum of ***s*-P2·Gd₂** (orange) and its best fit (blue).

Table S7. Fitted parameters of the curves in Fig. S38–S41.

	g	*D* (MHz)	*E* (MHz)	*D* strain (MHz)	*E* strain (MHz)	*J* (MHz)
**P1·Gd**	1.9993(10)	–3518±100	304±50	1166±100	468±50	n/a
***f*-P2-*Z*·Gd₂**	1.9938(10)	–3590±100	253±50	1498±100	348±50	–23.8±10
***f*-P2-*E*·Gd₂**	1.9954(10)	–3579±100	251±50	1416±100	367±50	–21.2±10
***s*-P2·Gd₂**	1.9930(10)	–3521±100	249±50	1427±100	290±50	0

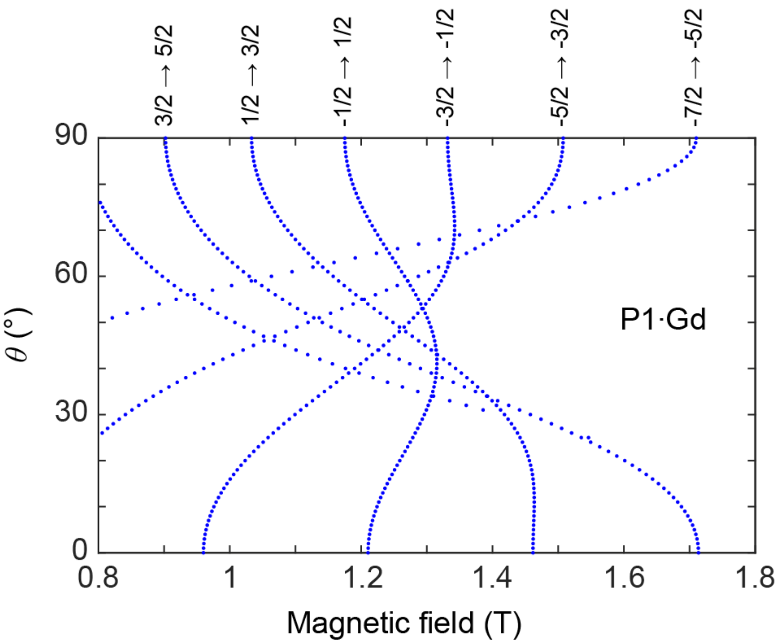

Figure S42. Orientation dependence of the single transitions of **P1·Gd** at Q-band frequency. The 0° orientation corresponds to the direction of the unique *z* quantization axis of the monomer. Averaging over all orientations produces the spectrum shown in Figure S38.

Picket-fence pulse sequence to determine spin-spin relaxation times T_1

In the picket-fence pulse sequence, a train of π-pulses (Fig. S43a) leads to a randomization of the spin orientations. From the randomized state, we then measure the signal recovery via echo-detection. The acquired data was then fitted with a biexponential function, where the first exponential accounts for the spin-spin relaxation time, and the second one with a typically faster time constant for spectral diffusion. Spectral diffusion typically arises from electron-electron or electron-nuclear interactions. The picket-fence technique helps to minimize their contributions. The pulse durations were set to 16 ns and 32 ns for the π/2- and π -pulses, respectively, and a 4-step phase-cycling procedure was carried out.

The data in Figure 7b of the main text were fitted using a combination of a linear term, typical for direct relaxation processes, and a term scaling with T^9, typical for Raman processes[8]:

$$T_1^{-1} = a_0 T + a_1 \left(\frac{T}{\theta_D}\right)^9 \int_0^{\theta_D/T} \frac{x^8 e^x}{(e^x - 1)^2} dx \,. \qquad (3)$$

Here, a_0 corresponds to the direct relaxation rate constant, a_1 to the Raman relaxation rate constant, θ_D the Debye temperature and T the temperature. We found a_0 = 1.90(27) K^{-1} s^{-1}, a_1 = 1503(834) s^{-1}, θ_D = 22.5(55) K for ***f*-P2-*Z*·Gd$_2$**, a_0 = 2.07(19) s^{-1}, a_1 = 1118(353) s^{-1}, θ_D = 18.0(27) K for ***f*-P2-*E*·Gd$_2$** and a_0 = 2.01(17) s^{-1}, a_1 = 2627(1423) s^{-1}, θ_D = 33.9(71) K for ***s*-P2·Gd$_2$**. Below 6 K a slight deviation is observed, stronger visible in ***f*-P2-*E*·Gd$_2$**, as typical of direct processes, in agreement with the processes limiting the ac spin dynamics of the Dy^{III} analogues.

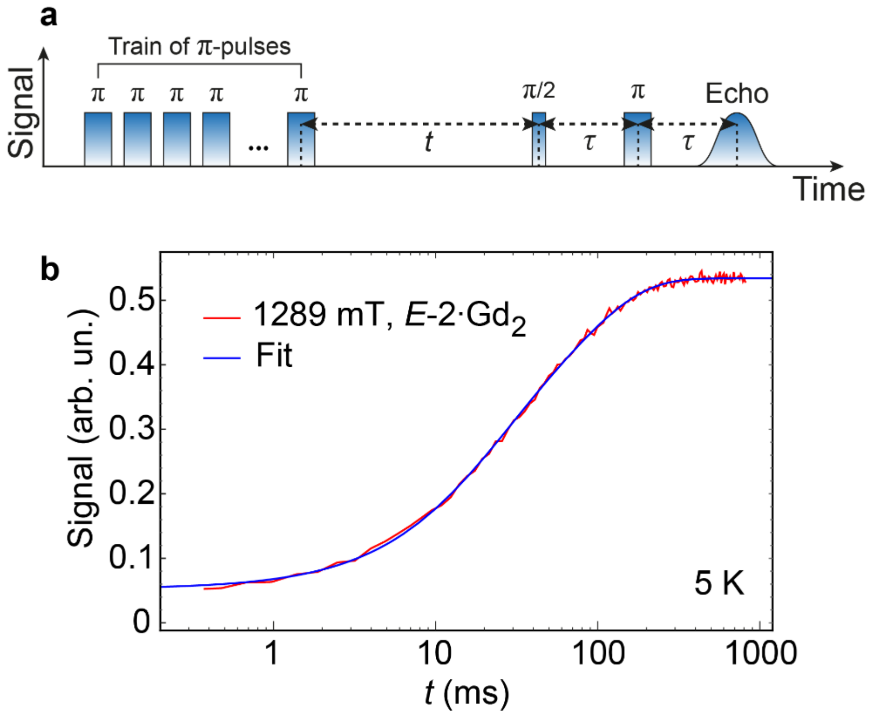

Figure S43. a) Picket-fence pulse sequence. b) Experimental data taken at a field of 1289 mT and 5 K for ***E*-P2-*E*·Gd$_2$**. The echo signal increases biexponentially when increasing the time delay *t*. The blue line represents the fit to the data (red).

9. Torque Magnetometry

Cantilever torque magnetometry (CTM) measures the magnetic torque,

$$\xi = M \times B, \tag{S1}$$

that arises when a magnetically anisotropic sample is placed in a homogenous magnetic field, *B*, that does not align with its magnetization, *M*. The torque is a consequence of the system seeking to minimize the free energy, *E*, through: $\xi = \partial E/\epsilon$, where ϵ is the angle between *M* and *B*. For paramagnetic systems τ can be expressed through orthogonal (*x*,*y*,*x*) components:[9]

$$\xi_y = B^2(M_x/B_x - M_z/B_z)\sin\epsilon\cos\epsilon\,. \tag{S2}$$

Equation S2 implies that $\xi \neq 0$ only if $M_x/B_x - M_z/B_z \neq 0$, i.e. only if the system is magnetically anisotropic. Therefore, CTM offers the convenient intrinsic property of being insensitive to isotropic contributions to *M* and only sensitive to anisotropic contribution. Moreover, the anisotropic component of *M* can be accessed through Equation S1 through,

$$M \propto \xi/B\,. \tag{S3}$$

We have used capacitance-detected CTM by map the magnetic anisotropy of the two isomers. The working principle of our method is to place a single crystal of the compound on a conducting diamagnetic copper beryllium cantilever that is separated by vacuum by a distance, *d*, from an underlying parallel CuBe plate. The two plates are connected to an AH2550 capacitance bridge that is highly sensitive to *d* through the capacitance, $C \propto 1/d$, and can detect the small torque-induced deflections. It can be shown that, $\xi_y = C_{B=0}/C(B) - 1$, which yields the qualitative τ-B behavior.[9]

We have used a home-built CTM device to investigate the magnetic anisotropy and magnetic hysteresis of single crystals of the two isomers. The single crystals were face indexed using single-crystal XRD to relate the orientation of the unit cell with the crystal faces. When the orientation was identified, the crystal could be placed on the cantilever in an orientation of choice. Subsequently, the CTM device was loaded into an Oxford Instruments Triton dilution refrigerator with a base temperature of 25 mK. The refrigerator is equipped with a superconducting 3D vector magnet capable of generating magnetic fields in all direction. Thereby, the device can be held fixed while the field direction is changed. The torque responses of selected planes were detected by rotating *B* with ϕ-increments of 2.5°, at *T* = 0.2 K unless otherwise specified. The crystal planes containing *ab* and *bc* were scanned for both isomers, ***f*-P2-*Z*-Dy$_2$** and ***f*-P2-*E*-Dy$_2$**, and the results are shown in Figure S44.

Moreover, magnetic hysteresis was measured by fixing the field direction and sweeping *B* back and forth between positive and negative magnitudes while continuously measuring the torque. The sweep rate was 0.125 T/min and *T* was 0.2 K unless otherwise specified. The magnetization was accessed through Equation S3 and normalized to yield unity at the maximum value calculated (from normalizing Equation S3) through $M \propto \xi B_{max}(B\xi_{max})^{-1}$. A limitation of this approach is the divergence of *M* values close to *B* = 0 because the torque vanishes at zero field (Eq. S1). Therefore, these artifacts around zero field are removed from the averaged curves in the hysteresis plots of Figure S44.

***f*-P2-*E*-Dy$_2$**. First we scanned the *ab* plane of the *anti* complex. Figure S44b reveals an 'easy' axis of magnetization around $\phi = 90°$, where ξ crosses zero, pointing along the porphyrin plane; a 'hard' axis around $\phi = 15°$ at a small tilt away from the Dy-Co bond direction; and an 'easy' plateau between $\phi = -5°$ and $10°$ pointing closely along the Dy-Co bond direction. Large magnetic hysteresis was observed close to the 'easy' axis at $275°/95°$ (A, Fig. S44c).

After reorienting the crystal on the cantilever to a different face, we scanned the *bc* plane. Figure S44d shows that this plane contains an 'easy' axis around $\phi = 112°$ that points along the porphyrin plane and close to the *b* axis; and a 'hard' axis at $\phi = 22°$ also in the porphyrin plane but with a $90°$ angle relative to the 'easy' axis. Common for both rotation planes is that they produce torque with similar magnitudes for positive and negative torque, as discussed in the main text. Magnetic

hysteresis was observed close to the 'easy' axis at 117°/297° (B, Fig. S44e). The hysteresis loop narrowed with temperature upon increasing T from 0.2 K to 1.4 K.

***t*-P2-*Z*-Dy$_2$.** First, we scanned the *ab* plane of the ***t*-P2-*Z*-Dy$_2$**. For this isomer we utilized that the monoclinic symmetry requires a unique magnetic axis to point along the *b* axis. Figure S44f reveals that a 'hard' axis coincides with *b* at $\phi = 107°$, which points along the short edge of the porphyrin plane. Around $\phi = -10°$ there is an 'easy' axis that points around 25° away from the Dy-Co axis. Between $\phi = 70°$ and $\phi = 90°$ lies an 'easy' plateau, where ξ is almost insensitive to ϕ, that points out from the Dy ion to between the porphyrin plane and the capping ligand. The torque curves are significantly shifted towards positive values and can be attributed to intramolecular interactions between the Dy ions, which causes strong permanent magnetism in the system and allows for tunneling of magnetization at certain fields. The hysteresis in this orientation, measured along 17°/197° (Γ, Fig. S44g) and along 80°/260° (E, Fig. S44h), is present but smaller than that of the ***t*-P2-*E*-Dy$_2$**.

Second, we reoriented the crystal on the cantilever and scanned the *bc* plane. Striking features are the 180°-wide torque peaks and a shift to almost completely positive torque values. These features indicate that this crystal plane is characterized by permanent magnetization that can flip through tunneling at certain fields. The crystallographic *b* axis is found close to the angle ($\phi = 90°$) that produces maximum torque. An 'easy' axis around $\phi = 0°$ is present where B aligns with *c*. However, the torque quickly grows to positive values already at $\phi = 20°$ that indicates that the magnetisation has flipped close to the 'easy' axis only to produce the same torque curves over again. Likely this reversal of magnetization is facilitated by transverse anisotropy terms that are symmetry-allowed because the two inequivalent Dy anisotropies are non-collinear. This phenomenon is in direct contrast to ***t*-P2-*E*-Dy$_2$** where such tunneling is quenched due to collinearity of the Dy anisotropies caused by the intramolecular inversion center. The hysteresis measured at 42°/222° (Δ, Fig. S44j) is slightly smaller than what was measured in ***t*-P2-*E*-Dy$_2$**.

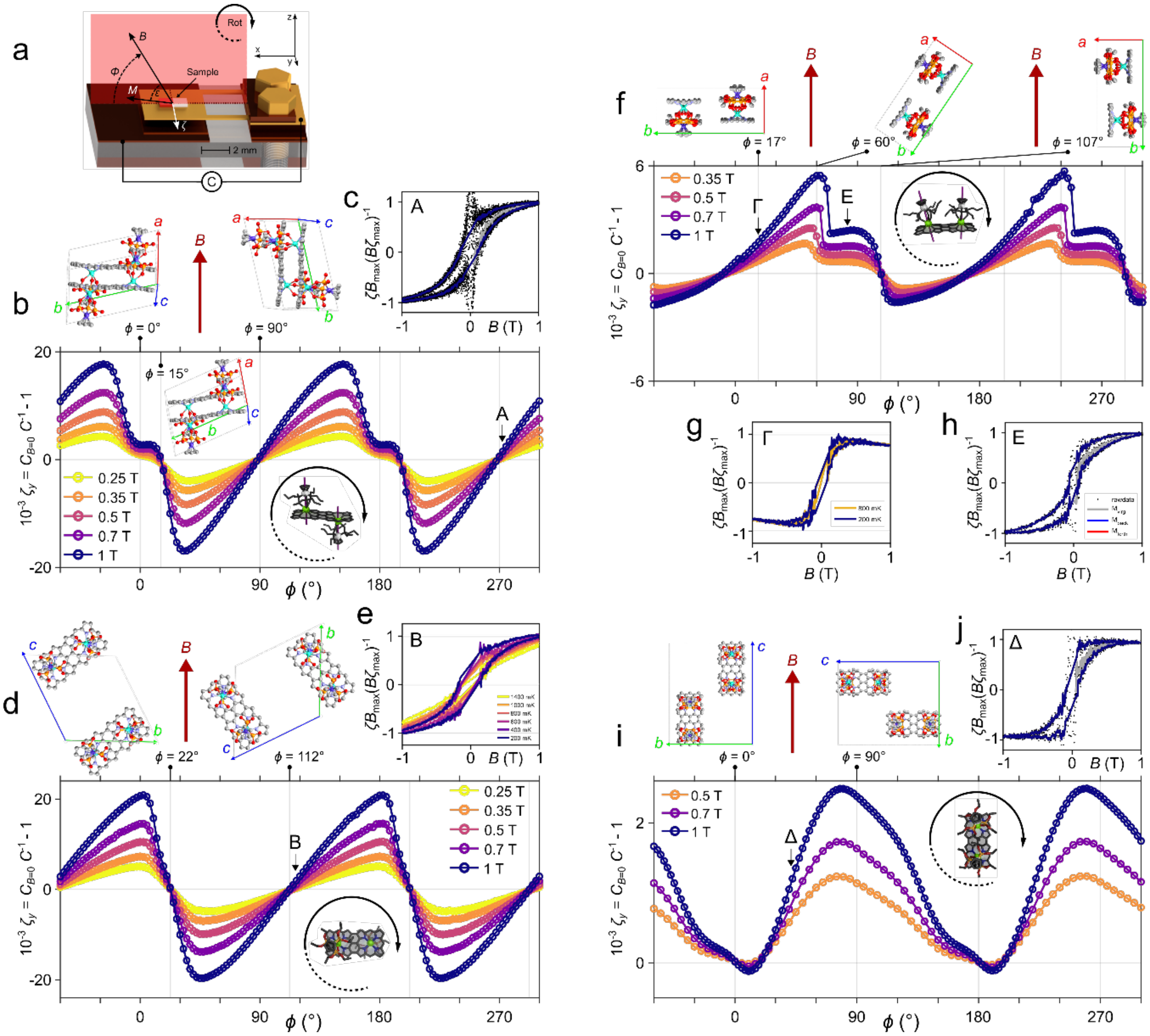

Figure S44: CTM schematics and measurements of *f*-P2-*E*-Dy_2 and *f*-P2-*Z*-Dy_2 single crystals at different orientations. T=0.2 K unless otherwise specified. (a) Schematics of the CTM setup for the rotation plane and capacitive detection. τ-ϕ curves obtained at different field magnitudes for the *f*-P2-*E*-Dy_2 (*f*-P2-*Z*-Dy_2) complex are shown in panels (b) and (d) ((f) and (i)). The four τ-ϕ plots result from rotating B in different rotation planes, as depicted above the panels in the graphical representations of the unit cells. The unit cells' orientations are relative to the field direction at indicated values of ϕ. The following rotation planes are probed: ~*ab* (b), ~*bc* (d), *ab* (f), *bc* (i). The hysteresis of the magnetization is captured in panels (c,e) for *f*-P2-*E*-Dy_2 and in (g,h,j) for *f*-P2-*Z*-Dy_2. The field directions in their respective planes are indicated with symbols corresponding to arrow labels in the τ-ϕ plots. The solid-colored lines are averages of the acquired data at different T (blue always 0.2 K) and the grey lines are virginal curves.

10. References

[1] Alberti, M. N.; Nowakowska, S.; Tzirakis, M. D.; Nowakowski, J.; Fesser, P.; Schweizer, W. B.; Shchyrba, A.; Thilgen, C.; Jung, T. A.; Diederich, F. Syntheses of *trans*-A_2B_2- and *trans*-A_2BC-porphyrins with polar 4'-(dimethylamino)tolan-4-yl substituents, and a screening protocol for vapor-phase deposition on metal surfaces. *Eur. J. Org. Chem.* **2014**, 5705–5719.

[2] Ouyang, Q.; Zhu, Y. Z.; Zhang, C. H.; Yan, K. Q.; Li, Y. C.; Zheng, J. Y. An efficient PIFA-mediated synthesis of fused diporphyrin and triply-singly interlacedly linked porphyrin array. *Org. Lett.* **2009**, *11*, 5266–5269.

[3] Kläui, W. The coordination chemistry and organometallic chemistry of tridentate oxygen ligands with π-donor properties. *Angew. Chem. Int. Ed.* **1990**, *29*, 627–637.

[4] Murakami, K.; Yamamoto, Y.; Yorimitsu, H.; Osuka, A. Demetalation of Metal Porphyrins via Magnesium Porphyrins by Reaction with Grignard Reagents. *Chem. Eur. J.* **2013**, *9*, 9123–9126.

[5] Ryan, A. A.; Senge, M. A. Synthesis and Functionalization of Triply Fused Porphyrin Dimers. *Eur. J. Org. Chem.* **2013**, 3700–3711.

[6] Tsuda, A.; Furuta, H.; Osuka, A. *J. Am. Chem. Soc.* **2001**, *123*, 10304–10321.

[7] Stoll, S.; Schweiger, A. EasySpin, a comprehensive software package for spectral simulation and analysis in EPR. *J. Magn. Reson.* **2006**, *178*, 42–55.

[8] Eaton, G. R.; Eaton, S. S. in Multifrequency Electron Paramagnetic Resonance: Theory and Applications (ed. Misra, S. K.) Ch. 17, Wiley, Weinheim, 2011.

[9] Cornia, A.; Affronte, M.; Jansen, A. G. M.; Gatteschi, D.; Caneschi, A.; Sessoli, R. Magnetic anisotropy of Mn_{12}-*acetate* nanomagnets from high-field torque magnetometry. *Chem. Phys. Lett.* **2000**, *322*, 477–482.
